# Asymmetric Organocatalysis in the Remote (3 + 2)-Cycloaddition to 4-(Alk-1-en-1-yl)-3-cyanocoumarins

**DOI:** 10.1021/acs.orglett.3c01189

**Published:** 2023-05-15

**Authors:** Beata Łukasik, Marta Romaniszyn, Nathan Kłoszewski, Łukasz Albrecht

**Affiliations:** † Institute of Organic Chemistry, Lodz University of Technology, Żeromskiego 116, 90-924 Łódź, Poland

## Abstract

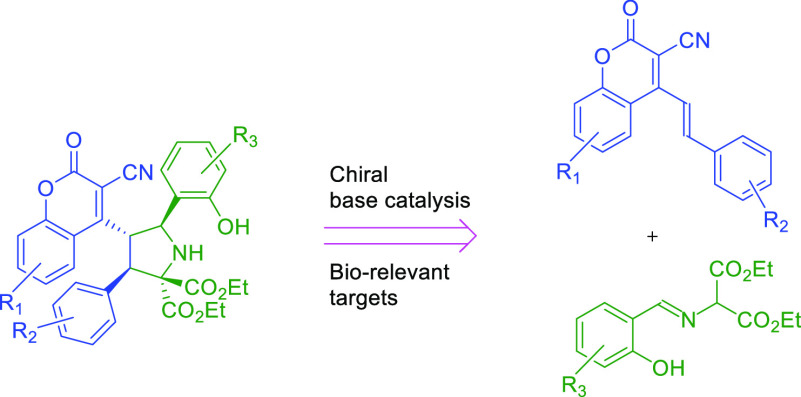

The application of organocatalytic bifunctional activation in the remote (3 + 2)-cycloaddition between 4-(alk-1-en-1-yl)-3-cyanocoumarins and imines derived from salicylaldehyde is demonstrated. Products, bearing two biologically relevant units, have been obtained with good chemical and stereochemical efficiency. The stereochemical outcome of the process results from the application of a quinine-derived catalyst. Selected transformations of the cycloadducts leading to further chemical diversity have been demonstrated.

The high demand of the life-science industry for enantiomerically pure molecules with defined absolute configurations resulted in the development of reliable methods for their preparation, with catalytic approaches being the most desired.^1^ Intensive studies in the area of asymmetric synthesis has led to the design of many attractive chiral catalysts for the conversion of pro-chiral substrates into enantiomerically enriched products. Until 2000, mainly transition metal complexes and enzymes were used as promoters of enantio-differentiating reactions.^2^ They offer many possibilities in the synthesis of nonracemic compounds, but at the same time they are not free from disadvantages related to catalyst availability and stability, toxicity, and the possibility to contaminate the reaction product with residues of the metal catalyst. Since the turn of the millennium, organocatalysis has emerged as an important alternative to these methods, providing many valuable solutions.^3,4^ Among the available methods, the application of organocatalysts derived from cinchona alkaloids has received great attention.^4^ This group of reaction promoters acts as bifunctional systems because they simultaneously activate both substrates, leading to highly efficient enantio- and diastereoselective transformations.^5^

Many natural products as well as synthetic analogues with significant biological activity contain the coumarin moiety and/or the pyrrolidine ring in their structure. The pharmacological properties of coumarins depend on their chemical scaffolds (core structure and substitution pattern) and the physicochemical properties of the oxaheterocyclic ring. The presence of a conjugated double bond system ensures the desired electronic properties and is crucial for the interaction with other molecules, receptors, and ions. Moreover, the planar, aromatic, and lipophilic nature of 2*H*-chromen-2-one results in a preference for hydrophobic interactions with aromatic amino acids, which allows for binding with the target protein.

Coumarins and their derivatives exhibit diverse bioactivities^6^ including antibacterial, antitubercular, antifungal, antiviral, antimutagenic, anti-inflammatory, anticancer, antioxidant, anticoagulant, and antithrombotic properties. They are also inhibitors of monoamine oxidase (MAO), cholinesterase (ChE), cyclooxygenase, and lipooxygenase and stimulants of the central nervous system (CNS) (Scheme 1).^7^ On the other hand, the five-membered pyrrolidine ring is a privileged structural motif in drug design.^8^ This structure tops the ranking of the most popular nonaromatic, five-membered nitrogen heterocycles and is present in 37 medications approved by the United States Food and Drug Administration.^9^ The pyrrolidine derivatives are widely distributed in alkaloids isolated from plant extracts or microorganisms^10^ that exhibit a broad spectrum of various biological properties, including anticancer, antimicrobial, antioxidant, antihyperglycemic, antifungal, or anti-inflammatory activities (Scheme 1). In recent years, stereocontrolled organocatalytic approaches for the preparation of both coumarin and pyrrolidine derivatives have gained increased importance.^11^

**Scheme 1:**
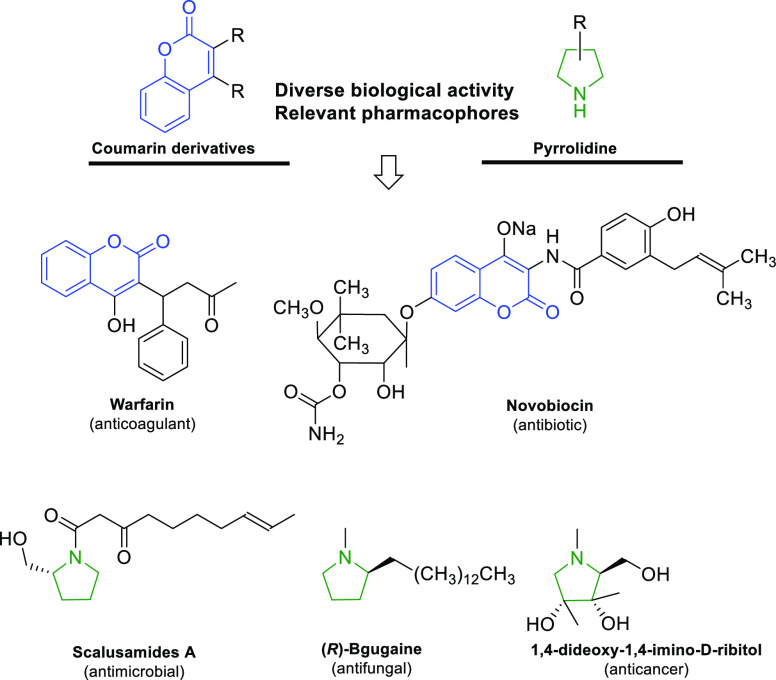
Representative Bioactive Compounds Containing a Coumarin or Pyrrolidine Skeleton

Given both the biological relevance of pyrrolidine or coumarin units and the synthetic potential of 4-(alk-1-en-1-yl)-3-cyanocoumarins, the task of the design and synthesis of hybrid molecules containing both biorelevant structural motifs was undertaken. The developed synthetic strategy relies on the application of the vinylogous reactivity of 4-(alk-1-en-1-yl)-3-cyanocoumarins as dienophiles in the 1,3-dipolar cycloaddition with imines (derivatives of salicyl aldehydes and appropriate amines, Scheme 2). At the outset of our studies, it was anticipated that the use of the cinchona-derived squaramide bifunctional catalyst would enable the control of both the chemical and stereochemical efficiency of the devised reactivity.

**Scheme 2:**
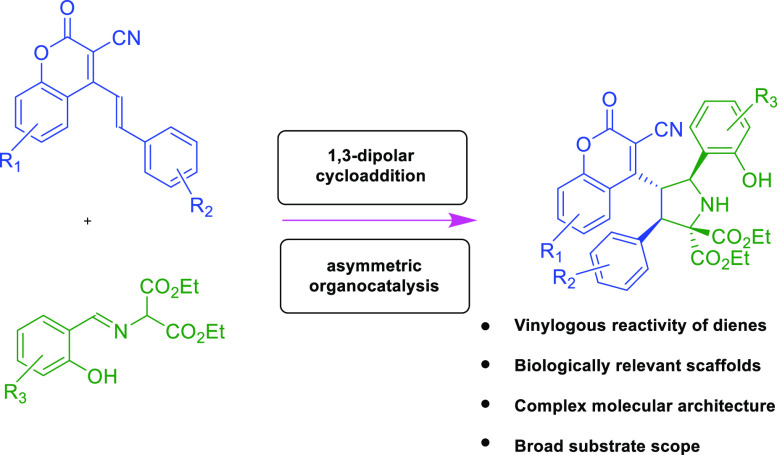
Synthetic Goals of Our Research

Coumarin **1a** and aldimine **2a** were employed as model reactants for the optimization studies (Table 1). Initial experiments were accomplished in the presence of catalysts **4a**–**f** in 1,2-dichloroethane as a solvent at 5 °C. Most of used catalysts **4a**–**e** gave access to product **3a** with high conversion after 72 h at 5 °C; however, **3a** was obtained in its racemic or nearly racemic form (Table 1, entries 1–5, respectively). Interestingly, when the reaction was conducted using the catalyst **4f** bearing a squaramide moiety and electron-withdrawing trifluoromethyl groups in the *meta*-positions of the aromatic substituent, a significant increase in enantioselectivity was observed without the loss of reactivity (Table 1, entry 6). The next step of our studies was devoted to the screening of solvents (Table 1, entries 7–12). A slight improvement of the conversion with a decrease of the enantiomeric ratio was observed for the reaction carried out in chloroform (Table 1, entry 7). The reaction performed in CH_2_Cl_2_ also yielded a product **3a** with excellent diastereo- and enantioselectivity (Table 1, entry 8). Utilization of either acetonitrile or toluene improved the reactivity, but the stereocontrol of the process was insufficient (Table 1, entries 9 and 10). The high conversion and diastereomeric ratio were obtained in α,α,α-trifluorotoluene and 4-fluorotoluene at 5 °C, but the enantioselectivity was lower (Table 1, entries 11 and 12, respectively). Finally, the influence of concentration and temperature on the process was investigated in dichloromethane (Table 1, entries 13–15). Additionally, it was demonstrated the reaction does not take place in the absence of a hydroxyl group in the benzaldimine derivative **2** (Table 1, entry 16).

**Table 1:**
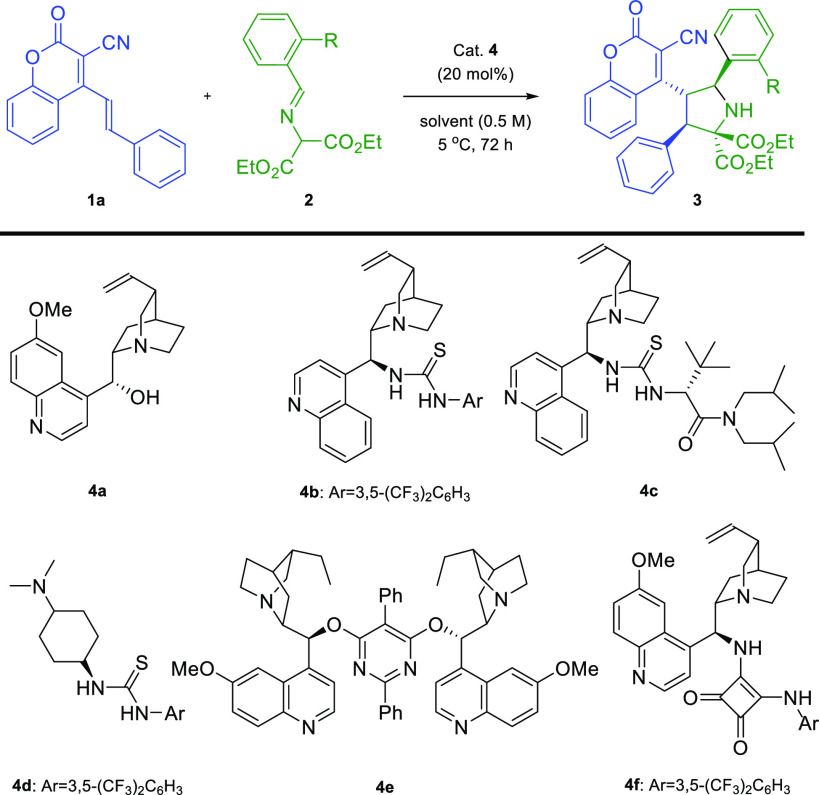
Asymmetric Remote (3 + 2)-Cycloaddition to 4-(Alk-1-en-1-yl)-3-cyanocoumarins **1**: Optimization Studies^a^

	cat.	**R**	solvent	conv [%]^b^	dr^c^	er^d^
1	**4a**	OH	ClCH_2_CH_2_Cl	>95 (80)	12:1	60:40
2	**4b**	OH	ClCH_2_CH_2_Cl	19 (10)	13:1	rac
3	**4c**	OH	ClCH_2_CH_2_Cl	>95 (68)	14:1	55:45
4	**4d**	OH	ClCH_2_CH_2_Cl	82 (42)	n.d.	65:35
5	**4e**	OH	ClCH_2_CH_2_Cl	62 (60)	11:1	rac
6	**4f**	OH	ClCH_2_CH_2_Cl	81 (73)	12:1	87:13
7	**4f**	OH	CHCl_3_	>95 (93)	13:1	66:34
8	**4f**	OH	CH_2_Cl_2_	75 (70)	>20:1	90:10
9	**4f**	OH	CH_3_CN	>95 (93)	12:1	80:20
10	**4f**	OH	PhCH_3_	>95 (65)	17:1	92:8
11	**4f**	OH	PhCF_3_	>95 (88)	>20:1	89:11
12	**4f**	OH	4-F-PhCH_3_	93 (81)	>20:1	87:13
13^e^	**4f**	OH	CH_2_Cl_2_	65 (60)	>20:1	93:7
14^e^^,^^f^	**4f**	OH	CH_2_Cl_2_	75 (70)	>20:1	92:7
15^g^	**4f**	OH	CH_2_Cl_2_	76 (70)	>20:1	93:7
16^g^	**4f**	H	CH_2_Cl_2_	<5	n.d.	n.d.

a Reactions were accomplished on a 0.05 mmol scale using **1a** (1 equiv) and **2** (1.2 equiv) in 0.2 mL of the solvent for 72 h.

b Conversion was evaluated by ^1^H NMR analysis of the crude reaction mixture. The isolated yield is given in parentheses.

c Determined by ^1^H NMR analysis of the crude reaction mixture.

d Determined by a chiral stationary phase UPC^2^.

e The reaction was carried out at −25 °C.

f The reaction was carried out in CH_2_Cl_2_ (0.1 mL).

g The reaction was carried out at −18 °C.

With optimization studies accomplished, the scope studies were initiated (Table 2, Scheme 3). Initially, the significance of various substituents in coumarins **1a**–**l** on the reaction outcome was tested (Table 2). To our delight, all reactions exhibited excellent diastereooselectivity. The application of halogen substituents in either *meta*- or *para*-positions of the styryl moiety in **1** resulted in high enantioselectivity and yields for the process (Table 2, entries 2–4). The compound **1d** with fluorine in the *meta*-position provided **3d** with a decreased yield (Table 2, entry 4). While the substrate **1e** bearing a trifluoromethyl group gave an excellent result in terms of the efficiency and stereoselectivity of the process (Table 2, entries 5), the presence of the nitro group in the *para*-position of the styryl moiety in **1f** led to deteriorated enantiomeric enrichment (Table 2, entry 6). Electron-donating substituents were well-tolerated, and cycloadditions with the substrates **1g**–**i** proceeded in good yields and excellent diastereo- and enantioselectivities (Table 2, entries 7–9, respectively). The incorporation of substituents on the aromatic ring of the coumarin unit turned out also possible giving satisfactory results for both electron-withdrawing (Table 2, entry 10) and electron-donating groups (Table 2, entry 11). Then, the influence of the electron-withdrawing cyano group in the 3-position on the outcome of the reaction was tested, and it was found that the cycloaddition with **1l** bearing a carboxylate moiety did not take place (Table 2, entry 12).

**Table 2:**
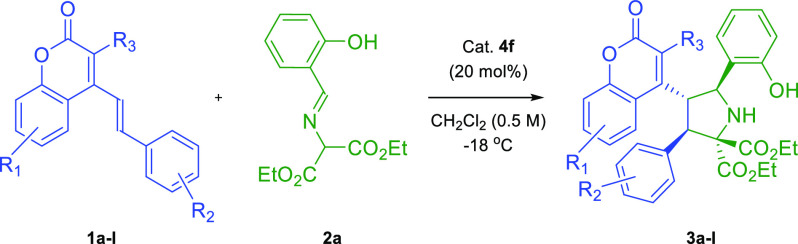
Asymmetric Remote (3 + 2)-Cycloaddition to 4-(Alk-1-en-1-yl)-3-cyanocoumarins **1a**–**k**: Scope Studies^a^

	R^1^	R^2^	R^3^	yield [%]^b^	er^c^
1	H	H	CN	73	93:7
2	H	4-Cl	CN	85	94:6
3	H	3-Cl	CN	71	93:7
4^d^	H	3-F	CN	58	93.5:6.5
5	H	4-CF_3_	CN	71	96:4
6	H	4-NO_2_	CN	78	87:13
7	H	4-Me	CN	73	95:5
8	H	3-Me	CN	56	90:10
9	H	4-OMe	CN	74	92:8
10	6-Br	H	CN	74	94:6
11	7-OMe	H	CN	40	96:4
12	H	H	CO_2_Et		

a Reactions were accomplished on a 0.05 mmol scale using **1a**–**k** (1 equiv), **2a** (1.2 equiv), and catalyst **4f** (20% mol) in CH_2_Cl_2_ (0.2 mL) at −18 °C for 72 h.

b The isolated yield is given.

c Determined by chiral stationary phase UPC^2^.

d The reaction was carried out for 5 days.

**Scheme 3:**
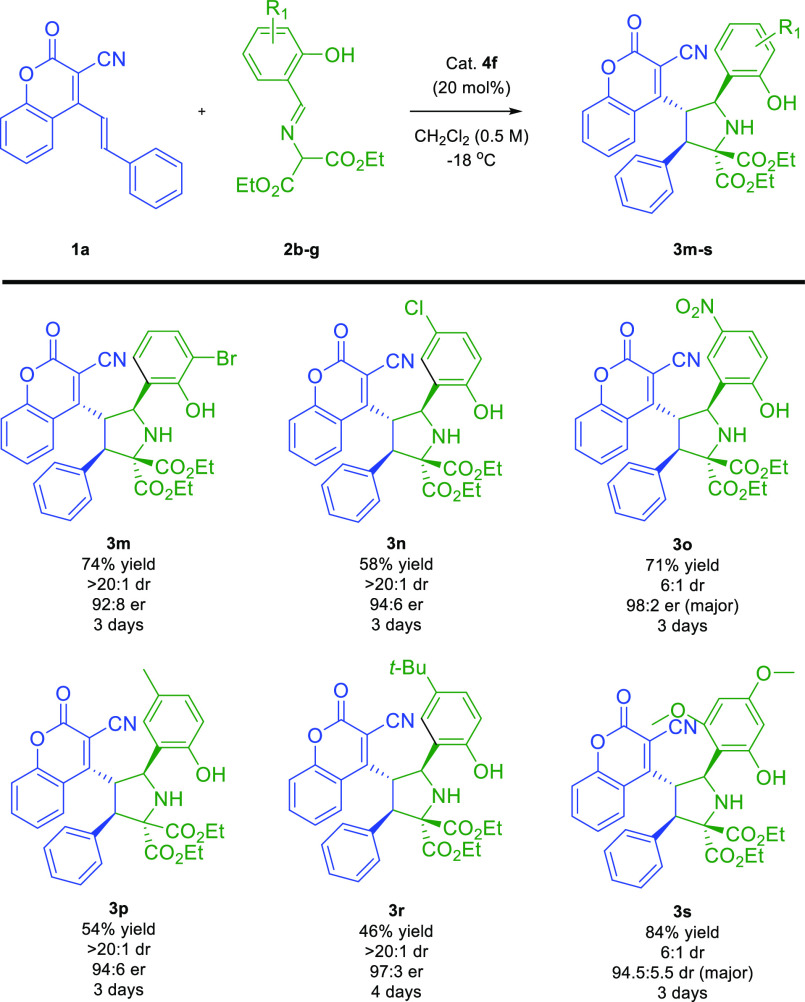
Asymmetric Remote (3 + 2)-Cycloaddition of *o*-Hydroxy Aromatic Aldimines **2b**–**g** to 4-(Alk-1-en-1-yl)-3-cyanocoumarin **1a**: Scope Studies

The possibility of employing various aldimines **2** in the investigated reaction was subsequently examined (Scheme 3). The cycloaddition of **2b**–**g** bearing different electron-withdrawing or electron-donating groups on the aromatic ring in **2** proceeded efficiently, providing the desired products in a highly enantioselective fashion (Scheme 3, compounds **3m**–**s**, respectively). Additionally, for **2b**, **2c**, **2e**, and **2f**, excellent diastereomeric ratios were observed (Scheme 3, compounds **3m**, **3n**, **3p**, and **3r**, respectively). Interestingly, a worsening of the diastereoselectivity of the process was observed for **2d** and **2g**, while its high efficiency and enantioselectivity were maintained (Scheme 3, compounds **3o** and **3s**, respectively).

Subsequently, the transformation of **3a**, **3b**, and **3m** into **5a**, **5b**, and **5c**, respectively, was demonstrated (Scheme 4). Therefore, the above substrates were subjected to the reaction with 1,1′-carbonyldiimidazole. The reaction was performed in dichloromethane at room temperature for 14 h, giving compounds **5**. Notably, the stereomeric composition of the starting material was not maintained under the employed conditions, resulting in the formation of **5** with slightly reduced diastereo- and enantioselectivity.

**Scheme 4:**
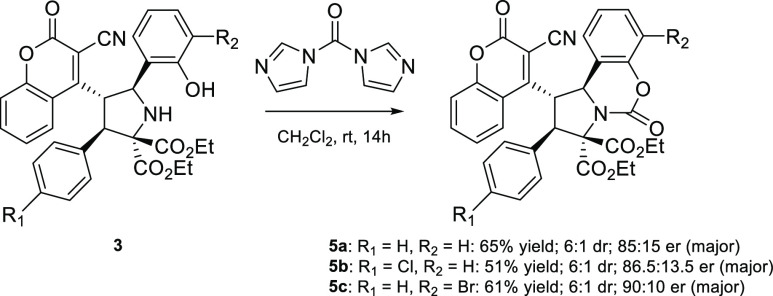
Condensation of **3** with 1,1′-Carbonyldiimidazole

The absolute configuration of product **5a** was unambiguously confirmed by the X-ray structural analysis (for details, see the SI).^12^ The stereochemistry of compounds **3b**–**s** has been assigned by analogy assuming the same mechanism for all cycloadditions performed. Given the configurational assignments, the mechanism and stereochemical model of the studied cycloaddition were proposed (Scheme 5). The process is initiated by the deprotonation of the corresponding aldimine **2a**, thus generating an ylide that forms a chiral ion pair with a catalyst **4f**. Simultaneously, 4-(alk-1-en-1-yl)-3-cyanocoumarin **1** is activated by the H-bond interactions with the squaramide moiety of the catalyst **4f**.^13^ This leads to a completely stereoselective generation of a five-membered heterocyclic ring by 1,3-dipolar cycloaddition, giving access to a wide range of derivatives **3**.

**Scheme 5:**
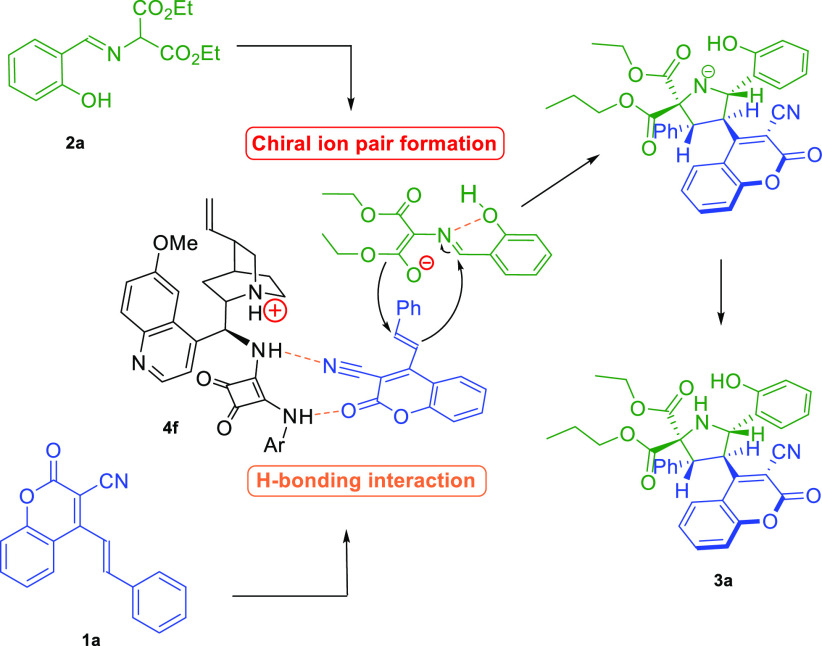
Asymmetric Remote (3 + 2)-Cycloaddition to 4-(Alk-1-en-1-yl)-3-cyanocoumarins **1**: Mechanistic Considerations

In conclusion, we have elaborated a new highly efficient enantio- and diastereoselective method for the preparation of pyrrolidine derivatives **3**. Our approach utilized organocatalytic (3 + 2)-cycloaddition between 4-(alk-1-en-1-yl)-3-cyanocoumarins **1** and imines **2** (derived from salicylaldehydes and diethyl aminomalonates), which proceeded selectively at the remote double bond in **1**. Target products bearing three adjacent stereogenic centers and a new heterocyclic pyrrolidine moiety were obtained with high chemical and stereochemical efficiency.

## Data Availability

The data underlying this study are available in the published article and its Supporting Information.

## Supporting Information Available

Experimental procedures, characterization of the products, crystal and X-ray data for **5a**, NMR data, and UPC^2^ traces (PDF)
