# Supplementary material for: Asymmetric Organocatalysis in the Remote (3 + 2)-Cycloaddition to 4-(Alk-1-en-1-yl)-3-cyanocoumarins
Source: Org Lett. 2023 May 15;25(20):3728–32. doi: 10.1021/acs.orglett.3c01189 (PMC10226170; doi:10.1021/acs.orglett.3c01189)
Supplement: Supplementary file 1 — ol3c01189_si_001.pdf [file ol3c01189_si_001.pdf]

Asymmetric organocatalysis in the remote (3+2)-cycloaddition to 4-(alk-1-en-1-yl)-3-cyanocoumarins

Beata Łukasik[a], Marta Romaniszyn[a], Nathan Kłoszewski[a] and Łukasz Albrecht[a*]

[a] Institute of Organic Chemistry, Department of Chemistry

Lodz University of Technology

Zeromskiego 116, 90-924 Lodz, Poland

E-mail: lukasz.albrecht@p.lodz.pl

Contents

1. General methods S2
2. Asymmetric remote (3+2)-cycloaddition with 4-(alk-1-en-1-yl)-3-cyanocoumarins **1** – general procedure S3
3. Enatioselective synthesis of (3*R*,4*R*,5*S*)-Diethyl 4-(3-cyano-2-oxo-2*H*-chromen-4-yl)-5-(2-hydroxyphenyl)-3-phenyl-pyrrolidine-2,2-dicarboxylate **3a** on a 1 mmol scale S11
4. Synthesis of compounds **5** – general procedure S12
5. Crystal and X-ray data for (1*R*,2*R*,10b*S*)-diethyl 1-(3-cyano-2-oxo-2*H*-chromen-4-yl)-5-oxo-2-phenyl- 5,10*b*-dihydro-1*H*-benzo[*e*]pyrrolo[1,2*c*][1,3]oxazine-3,3(2*H*)-dicarboxy-late **5a** S15
6. NMR data S17
7. UPC^2 traces S37

1. General methods

Unless otherwise noted, all reagents were purchased from commercial suppliers and used without purification. Thin layer chromatography was performed using pre-coated aluminum-backed plates (Merck Kieselgel 60 F254) and visualized by ultraviolet irradiation. Silica gel (Silica gel 60, 230-400 mesh, Fluka) was used for column chromatography. NMR spectra were acquired on a Bruker Ultra Shield 700 instrument, running at 700 MHz for ^{1}H and 176 MHz for ^{13}C or on a Jeol 400YH instrument, running at 400 MHz for ^{1}H and 101 MHz for ^{13}C, respectively. Chemical shifts (δ) were reported as part per million (ppm) in δ scale relative to residual solvent signals ($CDCl_3$: 7.26 ppm for ^{1}H NMR, 77.00 ppm for ^{13}C NMR). Multiplicities were given as: s (singlet); br s (broaded singlet); d (doublet); dd (doublet of doublets); ddd (doublet of doublet of doublets). Coupling constants (*J*) were reported in Hertz (Hz). High-resolution mass spectra (HRMS) were obtained on Bruker ESI-Q-TOF Impact II spectrometer using electrospray (ESI+) ionization. Optical rotations were measured on a Perkin-Elmer 241 polarimeter and $[\alpha]_D$ values are given in deg•cm•g^{-1}•dm^{-1}; concentration *c* is listed in g•(100 mL)$^{-1}$. The enantiomeric ratio (er) of the products were determined by Ultra Performance Convergence Chromatography (UPCC) using Daicel Chiralpak IA, IB and IC columns as chiral stationary phases. Melting points were uncorrected. 4-(Alk-1-en-1-yl)-3-cyanocoumarins **1**[1] and aldimines **2**[2] were synthesized according to the literature procedures and compounds described in the literature were characterized by comparison of their ^{1}H and ^{13}C NMR spectra to the previously reported data. Cinchona-alkaloid-derived catalyst **4f** was synthesized following the literature procedure.[3]

---

1. Romaniszyn, M.; Gronowska, K.; Albrecht, Ł. Remote Functionalization of 4-(Alk-1-en- 1-yl)-3-Cyanocoumarins via the Asymmetric Organocatalytic 1,6-Addition, *Adv. Synth. Catal.* **2021**, *363*, 1-7.
2. Cao, J.; Fang, R.; Liu, J.-Y.; Lu, H.; Luo, Y.-C.; Xu, P.-F. Organocatalytic Regiodivergent C−C Bond Cleavage of Cyclopropenones: A Highly Efficient Cascade Approach to Enantiopure Heterocyclic Frameworks, *Chem. Eur. J.* **2018**, *24*, 18863-18867.
3. Yang, W.; Du, D.-M. Highly Enantioselective Michael Addition of Nitroalkanes to Chalcones Using Chiral Squaramides as Hydrogen Bonding Organocatalysts, *Org. Lett.*,**2010**, *12*, 5450-5453.

2. **Asymmetric remote (3+2)-cycloaddition with 4-(alk-1-en-1-yl)-3-cyano-coumarins 1 – general procedure**

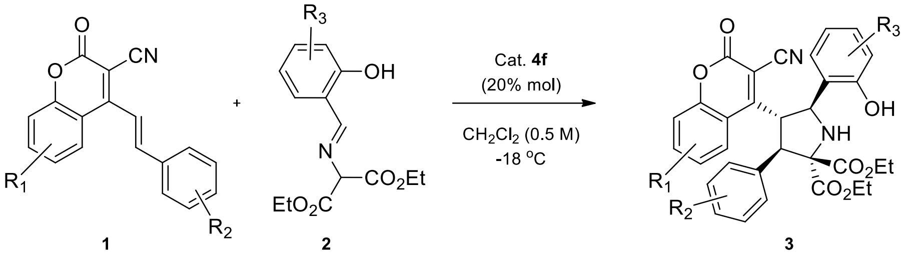

Scheme 1.

In an ordinary 4 mL glass vial, equipped with a magnetic stirring bar and a screw cap, the corresponding 4-(alk-1-en-1-yl)-3-cyanocoumarin **1** (1 equiv. 0.1 mmol) and appropriate aldimine **2** (1.2 equiv., 0.12 mmol) were dissolved in dichloromethane (0.2 mL). After cooling to –18 °C catalyst **4f** (20 mol%, 12.6 mg) was added and reaction was stirred for 3-5 days. Crude products **3** were purified by the column chromatography on silica gel using dichloromethane/acetone 100:1 as an eluent.

(3*R*,4*R*,5*S*)-Diethyl 4-(3-cyano-2-oxo-2*H*-chromen-4-yl)-5-(2-hydroxyphenyl)-3-phenyl-pyrrolidine-2,2-dicarboxylate **3a**

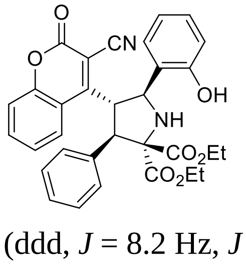

Following the general procedure, compound **3a** was isolated in 73% yield (40.1 mg) after 3 days as a yellowish solid**.** Mp = 91-92 °C; R_f = 0.42 (dichloromethane/acetone 100:1); $[\alpha]_D^{20}$= -6.1 (*c* 1, CH_2Cl_2); ^{1}H NMR (700 MHz, CDCl3) δ 10.77 (s, 1H), 7.62 (dd, *J* = 8.4 Hz, *J* = 1.3 Hz, 1H), 7.50 – 7.47 (m, 1H), 7.44 – 7.41 (m, 2H), 7.23 – 7.17 (m, 3H), 7.16 (dd, *J* = 8.3 Hz, *J* = 1.1 Hz, 1H), 7.14 (ddd, *J* = 8.5 Hz, *J* = 7.4 Hz, *J* = 1.2 Hz, 1H), 7.05 (ddd, *J* = 8.2 Hz, *J* = 7.4 Hz, *J* = 1.6 Hz, 1H), 6.94 (dd, *J* = 8.2 Hz, *J* = 1.1 Hz, 1H), 6.68 (dd, *J* = 7.5 Hz, *J* = 1.6 Hz, 1H), 6.51 (td, *J* = 7.4 Hz, *J* = 1.2 Hz, 1H), 5.54 (d, *J* = 12.4 Hz, 1H), 5.12 (dd, *J* = 10.6 Hz, *J* = 4.7 Hz, 1H), 5.06 (dd, *J* = 12.4 Hz, *J* = 10.6 Hz, 1H), 4.48 (dq, *J* = 10.7 Hz, *J* = 7.2 Hz, 1H), 4.40 (dq, *J* = 10.7 Hz, *J* = 7.1 Hz, 1H), 3.88 – 3.82 (m, 2H), 3.62 (dq, *J* = 10.7 Hz, *J* = 7.2 Hz, 1H), 1.41 (t, *J* = 7.2 Hz, 3H), 0.82 (t, *J* = 7.2 Hz, 3H); ^{13}C NMR (176 MHz, $CDCl_3$) *δ* (ppm) 170.0, 169.8, 161.8, 157.0, 156.9, 152.9, 135.3, 134.7, 130.0, 128.8 (2C), 128.6 (2C), 128.4, 128.3, 125.4, 125.1, 120.8, 119.7, 118.5, 118.0, 117.5, 113.8, 101.0, 75.1, 65.8, 63.3, 62.5, 52.8, 46.8, 14.0, 13.3; HRMS (ESI) m/z: $[M+H]^+$ Calcd for $C_{32}H_{29}N_2O_7$ 553.1969; Found 553.1971; UPC^2 (Chiralpack IC, CO_2/*i*-PrOH gradient from 100% up to 40%, flow rate = 2.2 mL/min, λ = 225.4 nm) tR = 4.20 min (major), 4.56 min (minor), 93:7 er.

(3*R*,4*R*,5*S*)-Diethyl 3-(4-chlorophenyl)-4-(3-cyano-2-oxo-2*H*-chromen-4-yl)-5-(2-hydroxy-phenyl)-pyrrolidine-2,2-dicarboxylate **3b**

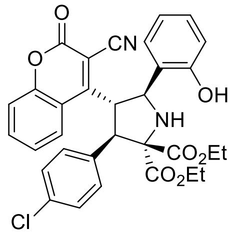

Following the general procedure, compound **3b** was isolated in 85% yield (49.8 mg) after 3 days as a colorless crystals**.** Mp = 98-100 °C; R*f* = 0.33 (dichloromethane/acetone 100:1); $[\alpha]_D^{20}$= -4.8 (*c* 1, CH_2Cl_2); ^{1}H NMR (700 MHz, $CDCl_3$) *δ* (ppm) 10.65 (s, 1H), 7.59 (d, *J* = 7.7 Hz, 1H), 7.53 – 7.49 (m, 1H), 7.38 – 7.36 (m, 2H), 7.21 – 7.19 (m, 2H), 7.18 (dd, *J* = 8.3 Hz, 1.1 Hz, 1H), 7.15 (ddd, *J* = 8.4 Hz, *J* = 7.4Hz, *J* = 1.2 Hz, 1H), 7.05 (ddd, *J* = 8.2 Hz, *J* = 7.4 Hz, *J* = 1.7 Hz, 1H), 6.94 (dd, *J* = 8.2 Hz, *J* = 1.1 Hz, 1H),

6.68 (dd, *J* = 7.5 Hz, *J* = 1.6 Hz, 1H), 6.52 (td, *J* = 7.4 Hz, *J* = 1.2 Hz, 1H), 5.52 (d, *J* = 12.6 Hz, 1H), 5.10 (dd, *J* = 10.5 Hz, *J* = 4.7 Hz, 1H), 5.00 (dd, *J* = 12.6 Hz, *J* = 10.7 Hz, 1H), 4.48 (dq, *J* = 10.7 Hz, *J* = 7.1 Hz, 1H), 4.40 (dq, *J* = 10.7 Hz, *J* = 7.2 Hz, 1H), 3.91 (dq, *J* = 10.7 Hz, *J* = 7.1 Hz, 1H), 3.85 (d, *J* = 4.7 Hz, 1H), 3.71 (dq, *J* = 10.8 Hz, *J* = 7.2 Hz, 1H), 1.40 (t, *J* = 7.2 Hz, 3H), 0.89 (t, *J* = 7.2 Hz, 3H); ^{13}C NMR (176 MHz, $CDCl_3$) δ (ppm) 169.82, 169.78, 161.3, 156.9, 156.8, 152.9, 135.5, 134.5, 133.2, 130.14, 130.11, 128.8 (2C), 128.4 (2C), 125.6, 124.8, 120.6, 119.8, 118.4, 118.0, 117.7, 113.7, 101.0, 74.8, 65.8, 63.4, 62.7, 52.1, 46.6, 13.9, 13.4; HRMS (ESI) m/z: $[M+H]^+$ Calcd for $C_{32}H_{28}ClN_2O_7$ 587.1579; Found 587.1574; UPC^2 (Chiralpack IA, CO_2/*i*-PrOH gradient from 100% up to 40%, flow rate = 2.2 mL/min, λ = 225.2 nm) tR = 3.76 min (major), 3.65 min (minor), 94:6 er.

(3*R*,4*R*,5*S*)-Diethyl 3-(3-chlorophenyl)-4-(3-cyano-2-oxo-2*H*-chromen-4-yl)-5-(2-hydroxy-phenyl)pyro-lidine-2,2-dicarboxylate **3c**

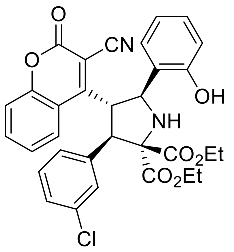

Following the general procedure, compound **3c** was isolated in 71% yield (41.7 mg) after 3 days as a colorless crystals. Mp = 118-120 °C; R_f = 0.28 (dichloromethane/acetone 100:1); $[\alpha]_D^{19}$= -5.7 (*c* 1, CH_2Cl_2); ^{1}H NMR (700 MHz, $CDCl_3$) δ (ppm) 10.64 (s, 1H), 7.63 – 7.61 (m, 1H), 7.53 – 7.50 (m, 1H), 7.42 (t, *J* = 1.8 Hz, 1H), 7.32 (dt, *J* = 7.7 Hz, *J* = 1.4 Hz, 1H), 7.20 – 7.14 (m, 4H), 7.06 (ddd, *J* = 8.2 Hz, *J* = 7.4 Hz, *J* = 1.7 Hz, 1H), 6.94 (dd, *J* = 8.2 Hz, *J* = 1.1 Hz, 1H), 6.69 (dd, *J* = 7.5 Hz, *J* = 1.6 Hz, 1H), 6.52 (td, *J* = 7.4 Hz, *J* = 1.2 Hz, 1H), 5.50 (d, *J* = 12.6 Hz, 1H), 5.11 (dd, *J* = 10.5 Hz, *J* = 4.8 Hz, 1H), 5.01 (dd, *J* = 12.6 Hz, *J* = 10.6 Hz, 1H), 4.49 (dq, *J* = 10.7 Hz, *J* = 7.2 Hz, 1H), 4.41 (dq, *J* = 10.7 Hz, *J* = 7.2 Hz, 1H), 3.91 (dq, *J* = 10.7 Hz, *J* = 7.1 Hz, 1H), 3.85 (d, *J* = 4.8 Hz, 1H), 3.73 (dq, *J* = 10.7 Hz, *J* = 7.2 Hz, 1H), 1.42 (t, *J* = 7.2 Hz, 3H), 0.89 (t, *J* = 7.2 Hz,3H); ^{13}C NMR (176 MHz, $CDCl_3$) δ (ppm) 169.8, 169.7, 161.2, 156.9, 156.8, 152.9, 136.7, 135.5, 134.5, 130.2, 129.8, 129.1, 128.7, 128.4, 126.8, 125.6, 124.9, 120.5, 119.8, 118.3, 118.1, 117.7, 113.8, 101.0, 74.8, 65.7, 63.5, 62.8, 52.2, 46.4, 14.0, 13.4; HRMS (ESI) m/z: $[M+H]^+$ Calcd for $C_{32}H_{28}ClN_2O_7$ 587.1579; Found 587.1575; UPC^2 (Chiralpack IC, CO_2/*i*-PrOH gradient from 100% up to 40%, flow rate = 2.2 mL/min, λ = 226.0 nm) tR = 3.99 min (major), 4.37 min (minor), 93:7 er.

(3*R*,4*R*,5*S*)-diethyl 4-(3-cyano-2-oxo-2*H*-chromen-4-yl)-3-(3-fluorophenyl)-5-(2-hydroxy-phenyl)pyro-lidine-2,2-dicarboxylate **3d**

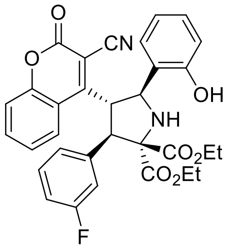

Following the general procedure, compound **3d** was isolated in 58% yield (33.1 mg) after 5 days as a colorless crystals. Mp = 98 °C; R_f = 0.28 (dichloromethane/acetone 100:1); $[\alpha]_D^{19}$= -5.8 (*c* 1, CH_2Cl_2); ^{1}H NMR (400 MHz, CDCl3) δ (ppm) 10.65 (s, 1H), 7.61 (d, *J* = 7.8 Hz, 1H), 7.51 (ddd, *J* = 8.5, 7.5, 1.4 Hz, 1H), 7.22 – 7.15 (m, 5H), 7.05 (ddd, *J* = 8.2, 7.4, 1.6 Hz, 1H), 6.94 (dd, *J* = 8.2, 1.0 Hz, 1H), 6.92 – 6.88 (m, 1H), 6.68 (dd, *J* = 7.5, 1.6 Hz, 1H), 6.52 (td, *J* = 7.4, 1.1 Hz, 1H), 5.54 (d, *J* = 12.6 Hz, 1H), 5.10 (dd, *J* = 10.5, 4.4 Hz, 1H), 5.00 (dd, *J* = 12.5, 10.7 Hz, 1H), 4.49 (dq, *J* = 10.7, 7.2 Hz, 1H), 4.41 (dq, *J* = 10.7, 7.2 Hz, 1H), 3.91 (dq, *J* = 10.7, 7.2 Hz, 1H), 3.86 (d, *J* = 4.7 Hz, 1H), 3.72 (dq, *J* = 10.7, 7.2 Hz, 1H), 1.41 (t, *J* = 7.2 Hz, 3H), 0.89 (t, *J* = 7.2 Hz, 3H); ^{13}C NMR (101 MHz, $CDCl_3$) δ (ppm) 169.80, 169.79, 162.6 (d, J_{C-F} = 247.4 Hz), 161.3, 156.95, 156.87, 152.9, 137.2 (d, J_{C-F} = 7.2 Hz), 135.5, 130.2, 130.1, 128.4, 125.6, 124.9, 124.34, 124.33, 120.6, 119.8, 118.3, 118.1, 117.6, 116.0 (d, J_{C-F} = 22.2 Hz), 115.5 (d, J_{C-F} = 20.9 Hz), 113.7, 101.0, 74.9,

65.8, 63.4, 62.7, 52.2, 46.6, 14.0, 13.4; HRMS (ESI) m/z: $[M+H]^+$ Calcd for $C_{32}H_{28}FN_2O_7$ 571.1874; Found 571.1879; UPC^2 (Chiralpack IC, CO_2/*i*-PrOH gradient from 100% up to 40%, flow rate = 2.2 mL/min, λ = 225.1 nm) tR = 3.75 min (major), 4.04 min (minor), 93.5:6.5 er.

(3*R*,4*R*,5*S*)-Diethyl 4-(3-cyano-2-oxo-2*H*-chromen-4-yl)-5-(2-hydroxyphenyl)-3-(4-(trifluoro-methyl)-phenyl)pyrrolidine-2,2-dicarboxylate **3e**

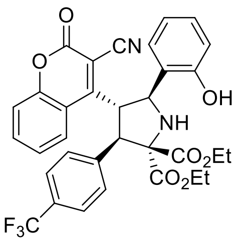

Following the general procedure, compound **3e** was isolated in 71% yield (44.1 mg) after 3 days as a colorless crystals**.** Mp = 98-100 °C; R_f = 0.4 (dichloromethane/acetone 100:1); $[\alpha]_D^{19}$= -5.8 (*c* 1, CH_2Cl_2); ^1H NMR (700 MHz, $CDCl_3$) δ (ppm) 10.62 (s, 1H), 7.61 (dd, *J* = 8.6 Hz, *J* = 1.0 Hz, 1H), 7.57 (d, *J* = 8.3 Hz, 2H), 7.53 – 7.51 (m, 1H), 7.49 (d, *J* = 8.3 Hz, 2H), 7.18 (dd, *J* = 8.3 Hz, *J* = 1.1 Hz, 1H), 7.16 (ddd, *J* = 8.5 Hz, *J* = 7.4 Hz, *J* = 1.2 Hz, 1H), 7.06 (ddd, *J* = 8.2 Hz, *J* = 7.4 hz, *J* = 1.7 Hz, 1H), 6.95 (dd, *J* = 8.2 Hz, *J* = 1.1 Hz, 1H), 6.69 (dd, *J* = 7.5 Hz, *J* = 1.7 Hz, 1H), 6.53 (td, *J* = 7.4 Hz, *J* = 1.2 Hz, 1H), 5.64 (d, *J* = 12.4 Hz, 1H), 5.12 (dd, *J* = 10.5 Hz, *J* = 4.7 Hz, 1H), 5.06 (dd, *J* = 12.3 Hz, *J* = 10.8 Hz, 1H), 4.49 (dq, *J* = 10.7 Hz, *J* = 7.2 Hz, 1H), 4.41 (dq, *J* = 10.7 Hz, *J* = 7.2 Hz, 1H), 3.90 – 3.84 (m, 2H), 3.66 (dq, *J* = 10.7 Hz, *J* = 7.1 Hz, 1H), 1.41 (t, *J* = 7.2 Hz, 3H), 0.81 (t, *J* = 7.2 Hz, 3H); ^{13}C NMR (176 MHz, $CDCl_3$) δ (ppm) 169.8, 169.6, 160.9, 156.9, 156.8, 152.9, 138.8, 135.6, 130.8 (d, *J* = 32.9 Hz), 130.2, 129.2, 128.4, 125.6, 125.5 (q, *J* = 3.7 Hz, 2C), 124.8, 123.7, 123.7 (q, *J* = 272.0 Hz), 120.5, 119.9, 118.3, 118.1, 117.7, 113.7, 101.1, 74.9, 65.9, 63.5, 62.8, 52.1, 46.3, 13.9, 13.2; HRMS (ESI) m/z: $[M+H]^+$ Calcd for $C_{33}H_{28}F_3N_2O_7$ 621.1843; Found 621.1844; UPC^2 (Chiralpack IA, CO_2/*i*-PrOH gradient from 100% up to 40%, flow rate = 2.2 mL/min, λ = 302.5 nm) tR = 3.35 min (major), 3.06 min (minor), 96:4 er.

(3*R*,4*R*,5*S*)-Diethyl 4-(3-cyano-2-oxo-2*H*-chromen-4-yl)-5-(2-hydroxyphenyl)-3-(4-nitro-phenyl)pyro-lidine-2,2-dicarboxylate **3f**

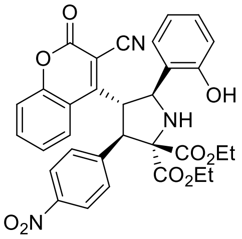

Following the general procedure, compound **3f** was isolated in 78% yield (46.5 mg) after 3 days as a yellowish solid**.** Mp = 158-160 °C; R_f = 0.27 (dichloromethane/acetone 100:1); $[\alpha]_D^{19}$= -3.3 (*c* 1, CH_2Cl_2); ^1H NMR (700 MHz, $CDCl_3$) δ (ppm) 10.52 (s, 1H), 8.11 – 8.07 (m, 2H), 7.65 – 7.62 (m, 2H), 7.60 (dd, *J* = 8.6 Hz, *J* = 0.9 Hz, 1H), 7.53 (ddd, *J* = 8.6 Hz, *J* = 7.4 Hz, *J* = 1.4 Hz, 1H), 7.20 – 7.16 (m, 2H), 7.09 – 7.06 (m, 1H), 6.95 (dd, *J* = 8.2 Hz, *J* = 1.1 Hz, 1H), 6.70 (dd, *J* = 7.5 Hz, *J* = 1.6 Hz, 1H), 6.54 (td, *J* = 7.4 Hz, *J* = 1.2 Hz, 1H), 5.67 (d, *J* = 12.5 Hz, 1H), 5.13 (dd, *J* = 10.4 Hz, *J* = 4.8 Hz, 1H), 5.07 (dd, *J* = 12.5 Hz, *J* = 10.6 Hz, 1H), 4.50 (dq, *J* = 10.7 Hz, *J* = 7.2 Hz, 1H), 4.42 (dq, *J* = 10.7 Hz, *J* = 7.1 Hz, 1H), 3.93 (dq, *J* = 10.8 Hz, *J* = 7.2 Hz, 1H), 3.89 (d, *J* = 4.8 Hz, 1H), 3.68 (dq, *J* = 10.8 Hz, *J* = 7.1 Hz, 1H), 1.41 (t, *J* = 7.2 Hz, 3H), 0.88 (t, *J* = 7.2 Hz, 3H); ^{13}C NMR (176 MHz, $CDCl_3$) δ (ppm) 169.6, 169.4, 160.6, 156.8, 156.6, 152.9, 147.9, 142.1, 135.7, 130.4 (2C), 129.9 (2C), 128.4, 125.7, 124.6, 123.7 (2C), 120.2, 120.0, 118.2, 117.8, 113.7, 101.1, 74.8, 65.9, 63.7, 62.9, 52.1, 46.4, 14.0, 13.5; HRMS (ESI) m/z: $[M+H]^+$ Calcd for $C_{32}H_{28}N_3O_9$ 598.1820; Found 598.1818; UPC^2 (Chiralpack IA, CO_2/*i*-PrOH gradient from 100% up to 40%, flow rate = 2.2 mL/min, λ = 227.1 nm) tR = 4.09 min (major), 3.67 min (minor), 87:13 er.

(3*R*,4*R*,5*S*)-Diethyl 4-(3-cyano-2-oxo-2*H*-chromen-4-yl)-5-(2-hydroxyphenyl)-3-(*p*-tolyl)-pyrolidine-2,2-dicarboxylate **3g**

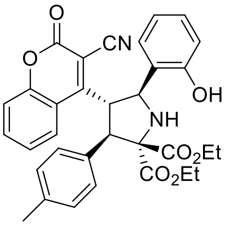

Following the general procedure, compound **3g** was isolated in 73% yield (41.3 mg) after 3 days as a yellowish solid. Mp = 93-95 °C; R_f = 0.3 (dichloromethane/acetone 100:1); $[\alpha]_D^{21}$= -3.6 (*c* 1, CH_2Cl_2); ^{1}H NMR (700 MHz, $CDCl_3$) δ (ppm) 10.77 (s, 1H), 7.62 (d, *J* = 7.6 Hz, 1H), 7.48 (ddd, *J* = 8.6 Hz, *J* = 7.4 Hz, *J* = 1.4 Hz, 1H), 7.30 (d, *J* = 8.2 Hz, 2H), 7.17 – 7.12 (m, 2H), 7.04 (ddd, *J* = 8.2 Hz, *J* = 7.4 Hz, *J* = 1.6 Hz, 1H), 7.01 (d, *J* = 7.9 Hz, 2H), 6.93 (dd, *J* = 8.2 Hz, *J* = 1.1 Hz, 1H), 6.68 (dd, *J* = 7.5 Hz, *J* = 1.6 Hz, 1H), 6.51 (td, *J* = 7.4 Hz, *J* = 1.2 Hz, 1H), 5.50 (d, *J* = 12.4 Hz, 1H), 5.10 (dd, *J* = 10.6 Hz, *J* = 4.7 Hz, 1H), 5.03 (dd, *J* = 12.3 Hz, *J* = 10.7 Hz, 1H), 4.48 (dq, *J* = 10.7 Hz, *J* = 7.1 Hz, 1H), 4.39 (dq, *J* = 10.7 Hz, *J* = 7.2 Hz, 1H), 3.89 – 3.84 (m, 2H), 3.67 (dq, *J* = 10.7 Hz, *J* = 7.2 Hz, 1H), 2.22 (s, 3H), 1.41 (t, *J* = 7.2 Hz, 3H), 0.85 (t, *J* = 7.2 Hz, 3H); ^{13}C NMR (176 MHz, $CDCl_3$) δ (ppm) 170.1, 169.9, 161.9, 157.0 (2C), 152.9, 138.3, 135.2, 131.5, 130.0 (2C), 129.2 (2C), 128.6, 128.3, 125.4, 125.1, 120.9, 119.7, 118.5, 118.0, 117.5, 113.8, 100.9, 75.0, 65.8, 63.2, 62.5, 52.5, 46.7, 20.9, 14.0, 13.3; HRMS (ESI) m/z: $[M+H]^+$ Calcd for $C_{33}H_{31}N_2O_7$ 567.2125; Found 567.2124; UPC^2 (Chiralpack IC, CO_2/*i*-PrOH gradient from 100% up to 40%, flow rate = 2.2 mL/min, λ = 224.8 nm) tR = 4.28 min (major), 4.61 min (minor), 94:6 er.

(3*R*,4*R*,5*S*)-Diethyl 4-(3-cyano-2-oxo-2*H*-chromen-4-yl)-5-(2-hydroxyphenyl)-3-(*m*-tolyl)-pyrrolidine-2,2-dicarboxylate **3h**

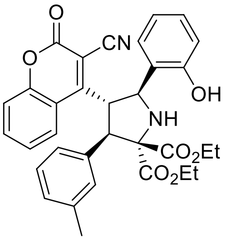

Following the general procedure, compound **3h** was isolated in 56% yield (31.5 mg) after 3 days as a colorless crystals. Mp = 108-110 °C; R_f = 0.33 (dichloromethane/acetone 100:1); $[\alpha]_D^{21}$= -4.4 (*c* 1, CH_2Cl_2); ^{1}H NMR (700 MHz, $CDCl_3$) δ (ppm) 7.63 (dd, *J* = 8.5, 1.2 Hz, 1H), 7.49 (ddd, *J* = 8.5, 7.4, 1.4 Hz, 1H), 7.22 (br s, 1H), 7.20 (d, *J* = 8.1 Hz, 1H), 7.17 – 7.13 (m, 2H), 7.08 (t, *J* = 7.7 Hz, 1H), 7.06 – 7.03 (m, 1H), 6.99 (d, *J* = 6.9 Hz, 1H), 6.94 (dd, *J* = 8.2, 1.1 Hz, 1H), 6.69 (dd, *J* = 7.5, 1.6 Hz, 1H), 6.51 (td, *J* = 7.4, 1.2 Hz, 1H), 5.49 (d, *J* = 12.5 Hz, 1H), 5.12 (d, *J* = 10.6 Hz, 1H), 5.04 (dd, *J* = 12.5, 10.6 Hz, 1H), 4.48 (dq, *J* = 10.7, 7.2 Hz, 1H), 4.40 (dq, *J* = 10.7, 7.1 Hz, 1H), 3.85 (dq, *J* = 10.7, 7.1 Hz, 1H), 3.65 (dq, *J* = 10.7, 7.2 Hz, 1H), 2.23 (s, 3H), 1.41 (t, *J* = 7.2 Hz, 3H), 0.83 (t, *J* = 7.2 Hz, 3H); ^{13}C NMR (176 MHz, $CDCl_3$) δ (ppm) 170.0, 169.9, 161.8, 157.02, 157.01, 152.9, 138.2, 135.3, 134.5, 130.0, 129.8, 129.1, 128.41, 128.35, 125.43, 125.38, 125.1, 120.8, 119.7, 118.5, 118.0, 117.6, 113.8, 101.0, 75.0, 65.7, 63.2, 62.5, 52.7, 46.6, 21.3, 14.0, 13.3; HRMS (ESI) m/z: $[M+H]^+$ Calcd for $C_{33}H_{31}N_2O_7$ 567.2125; Found 567.2123; UPC^2 (Chiralpack IC, CO_2/*i*-PrOH gradient from 100% up to 40%, flow rate = 2.2 mL/min, λ = 225.9 nm) tR = 4.15 min (major), 4.59 min (minor), 90:10 er.

(3*R*,4*R*,5*S*)-Diethyl 4-(3-cyano-2-oxo-2*H*-chromen-4-yl)-5-(2-hydroxyphenyl)-3-(4-methoxyphenyl)-pyrrolidine-2,2-dicarboxylate **3i**

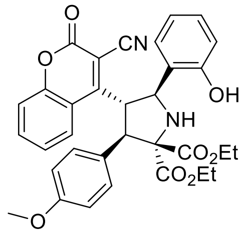

Following the general procedure, compound **3i** was isolated in 74% yield (43.2 mg) after 3 days as a colorless crystals. Mp = 102 °C; R_f = 0.3 (dichloromethane/acetone 100:1); $[\alpha]_D^{20}$= -2.4 (*c* 1, CH_2Cl_2); ^{1}H NMR (700 MHz, $CDCl_3$) δ (ppm) 7.52 (d, *J* = 8.3 Hz, 1H), 7.39 (ddd, *J* = 8.4 Hz, *J* = 7.4 Hz, *J* = 1.3 Hz, 1H), 7.25 – 7.22 (m, 1H), 7.08 – 7.02 (m, 2H), 6.94 (ddd, *J* = 8.2 Hz, *J* = 7.4 Hz, *J* = 1.6 Hz, 2H), 6.85 (d, *J* = 7.7 Hz, 1H), 6.64 (dd, *J* = 6.8 Hz, *J* = 5.1 Hz, 2H), 6.58 (dd, *J* = 7.5 Hz, *J* = 1.5 Hz, 1H), 6.41 (td, *J* = 7.4 Hz, *J* = 1.1 Hz, 1H), 5.38 (d, *J* = 12.5 Hz, 1H), 5.00 (d, *J* = 10.6 Hz, 1H), 4.92 (dd, *J* = 12.3 Hz, *J* = 10.9 Hz, 1H), 4.89 – 4.35 (m, 1H), 4.30 (dq, *J* = 10.7 Hz, *J* = 7.2 Hz, 1H), 3.79 (dq, *J* = 8.4 Hz, *J* = 7.1 Hz, 1H), 3.60 (s, 3H), 3.59 – 3.56 (m, 1H), 1.31 (t, *J* = 7.2 Hz, 3H), 0.79 (t, *J* = 7.2 Hz, 3H); ^{13}C NMR (176 MHz, $CDCl_3$) δ (ppm) 159.6, 157.0, 156.9, 152.9, 135.3, 130.1 (2C), 129.9 (2C), 128.4 (2C), 126.3, 125.5, 125.1 (2C), 119.7, 118.5, 118.0, 117.6, 113.95, 113.92 (2C), 113.8, 100.9, 75.0, 65.7, 63.3, 62.6, 55.2, 52.3, 46.8, 14.0, 13.5; HRMS (ESI) m/z: $[M+H]^+$ Calcd for $C_{33}H_{31}N_2O_8$ 583.2075; Found 583.2071; UPC^2 (Chiralpack IC, CO_2/*i*-PrOH gradient from 100% up to 40%, flow rate = 2.2 mL/min, λ = 227.8 nm) tR = 4.31 min (major), 4.64 min (minor), 92:8 er.

(3*R*,4*R*,5*S*)-Diethyl 4-(6-bromo-3-cyano-2-oxo-2*H*-chromen-4-yl)-5-(2-hydroxyphenyl)-3-phenylpyrrolidine-2,2-dicarboxylate **3j**

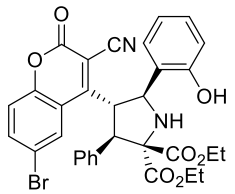

Following the general procedure, compound **3j** was isolated in 74% yield (46.7 mg) after 3 days as a yellowish solid. Mp = 106-108 °C; R_f = 0.38 (dichloromethane/acetone 100:1); $[\alpha]_D^{20}$= -6.1 (*c* 1, CH_2Cl_2); ^{1}H NMR (700 MHz, $CDCl_3$) δ (ppm) 7.66 (d, *J* = 2.1 Hz, 1H), 7.55 (dd, *J* = 8.7 Hz, *J* = 2.2 Hz, 1H), 7.44 – 7.41 (m, 2H), 7.28 – 7.26 (m, 1H), 7.26 – 7.21 (m, 2H), 7.10 (ddd, *J* = 8.8 Hz, *J* = 5.4 Hz, *J* = 1.7 Hz, 1H), 7.06 – 7.01 (m, 2H), 6.64 (dd, *J* = 7.5 Hz, *J* = 1.6 Hz, 1H), 6.51 (td, *J* = 7.4 Hz, *J* = 1.2 Hz, 1H), 5.60 (d, *J* = 12.5 Hz, 1H), 5.00 (d, *J* = 10.5 Hz, 1H), 4.91 (dd, *J* = 12.5 Hz, *J* = 10.6 Hz, 1H), 4.48 (dq, *J* = 10.7 Hz, *J* = 7.2 Hz, 1H), 4.40 (dq, *J* = 10.7 Hz, *J* = 7.1 Hz, 1H), 3.86 (dq, *J* = 10.7 Hz, *J* = 7.1 Hz, 1H), 3.63 (dq, *J* = 10.7 Hz, *J* = 7.2 Hz, 1H), 1.41 (t, *J* = 7.2 Hz, 3H), 0.82 (t, *J* = 7.2 Hz, 3H); ^{13}C NMR (176 MHz, $CDCl_3$) δ (ppm) 169.9, 169.8, 160.6, 157.0, 156.3, 151.6, 137.9, 134.6, 130.3, 128.8 (2C), 128.7 (2C), 128.6, 128.1, 127.7, 120.6, 119.9, 119.8, 119.0, 118.6, 118.2, 113.3, 102.0, 74.9, 66.1, 63.3, 62.7, 52.3, 46.9, 14.0, 13.3; HRMS (ESI) m/z: $[M+H]^+$ Calcd for $C_{32}H_{28}BrN_2O_7$ 631.1075; Found 631.1073; UPC^2 (Chiralpack IC, CO_2/*i*-PrOH gradient from 100% up to 40%, flow rate = 2.2 mL/min, λ = 226.1 nm) tR = 4.26 min (major), 4.45 min (minor), 94:6 er.

(3*R*,4*R*,5*S*)-Diethyl 4-(3-cyano-7-methoxy-2-oxo-2*H*-chromen-4-yl)-5-(2-hydroxyphenyl)-3-phenylpyrrolidine-2,2-dicarboxylate **3k**

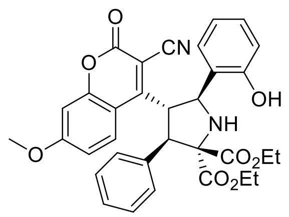

Following the general procedure, compound **3k** was isolated in 40% yield (23.3 mg) after 3 days as a yellowish solid. Mp = 126-128 °C; R_f = 0.24 (dichloromethane/acetone 100:1); $[\alpha]_D^{21}$= -7.6 (*c* 1, CH_2Cl_2); ^{1}H NMR (700 MHz, CDCl3) δ (ppm) 10.77 (s, 1H), 7.50 (d, *J* = 9.5 Hz, 1H), 7.43 – 7.40 (m, 2H), 7.23 – 7.17 (m, 3H), 7.06 (ddd, *J* = 8.2 Hz, *J* = 7.4 Hz, *J* = 1.7 Hz, 1H), 6.93 (dd, *J* = 8.2 Hz, *J* = 1.2 Hz, 1H), 6.70

(dd, J = 7.5 Hz, J = 1.6 Hz, 1H), 6.67 (dd, J = 9.2 Hz, J = 2.6 Hz, 1H), 6.59 (d, J = 2.6 Hz, 1H), 6.54 (td, J = 7.4 Hz, J = 1.2 Hz, 1H), 5.50 (d, J = 12.6 Hz, 1H), 5.11 (dd, J = 10.5 Hz, J = 4.8 Hz, 1H), 4.95 (dd, J = 12.5 Hz, J = 10.7 Hz, 1H), 4.47 (dq, J = 10.7 Hz, J = 7.2 Hz, 1H), 4.39 (dq, J = 10.7 Hz, J = 7.1 Hz, 1H), 3.86 – 3.81 (m, 2H), 3.79 (s,3H), 3.61 (dq, J = 10.7 Hz, J = 7.2 Hz, 1H), 1.40 (t, J = 7.2 Hz, 3H), 0.81 (t, J = 7.2 Hz, 3H); ^{13}C NMR (176 MHz, $CDCl_3$) δ (ppm) 170.1, 169.9, 165.5, 161.3, 157.8, 157.0, 155.4, 134.8, 129.9, 128.8 (2C), 128.5 (2C), 128.43, 128.37, 126.5, 121.0, 119.7, 117.9, 114.4, 114.0, 112.4, 101.0, 96.7, 75.1, 65.6, 63.2, 62.5, 56.0, 52.8, 47.1, 14.0, 13.3; HRMS (ESI) m/z: $[M+H]^+$ Calcd for $C_{33}H_{31}N_2O_8$ 583.2075; Found 583.2075; UPC^2 (Chiralpack IC, CO_2/*i*-PrOH gradient from 100% up to 40%, flow rate = 2.2 mL/min, λ = 226.1 nm) tR = 4.81 min (major), 5.24 min (minor), 96:4 er.

(3*R*,4*R*,5*S*)-Diethyl 5-(3-bromo-2-hydroxyphenyl)-4-(3-cyano-2-oxo-2*H*-chromen-4-yl)-3-phenylpyrrolidine-2,2-dicarboxylate **3m**

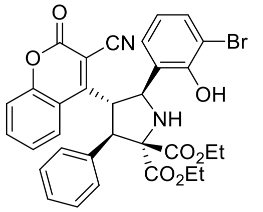

Following the general procedure, compound **3m** was isolated in 74% yield (41.3 mg) after 3 days as a colorless crystals. Mp = 98-100 °C; R_f = 0.5 (dichloromethane/acetone 100:1); $[\alpha]_D^{19}$= -13.1 (*c* 1, CH_2Cl_2); ^{1}H NMR (700 MHz, $CDCl_3$) δ (ppm) 11.46 (s, 1H), 7.57 (dd, J = 8.7 Hz, J = 1.0 Hz, 1H), 7.53 – 7.49 (m, 1H), 7.44 – 7.41 (m, 2H), 7.31 (dd, J = 7.9 Hz, J = 1.5 Hz, 1H), 7.23 – 7.16 (m, 5H), 6.65 (dd, J = 7.6 Hz, J = 1.5 Hz, 1H), 6.42 – 6.39 (m, 1H), 5.53 (d, J = 12.5 Hz, 1H), 5.10 (dd, J = 10.6 Hz, J = 4.4 Hz, 1H), 5.02 (dd, J = 12.3 Hz, J = 10.9 Hz, 1H), 4.48 (dq, J = 10.7 Hz, J = 7.2 Hz, 1H), 4.40 (dq, J = 10.7 Hz, J = 7.1 Hz, 1H), 3.91 (d, J = 4.6 Hz, 1H), 3.87 (dq, J = 10.7 Hz, J = 7.2 Hz, 1H), 3.62 (dq, J = 10.7 Hz, J = 7.2 Hz, 1H), 1.41 (t, J = 7.2 Hz, 3H), 0.80 (t, J = 7.2 Hz, 3H); ^{13}C NMR (176 MHz, $CDCl_3$) δ (ppm) 169.9, 169.6, 161.2, 156.8, 153.6, 152.9, 135.5, 134.4, 133.5, 128.7 (2C), 128.6 (2C), 128.5, 127.5, 125.5, 124.9, 122.3, 120.5, 118.3, 117.7, 113.7, 112.1, 101.0, 75.0, 65.6, 63.4, 62.7, 52.6, 46.5, 14.0, 13.3; HRMS (ESI) m/z: $[M+H]^+$ Calcd for $C_{32}H_{28}BrN_2O_7$ 631.1075; Found 631.1077; UPC^2 (Chiralpack IA, CO_2/*i*-PrOH gradient from 100% up to 40%, flow rate = 2.2 mL/min, λ = 227.0 nm) tR = 3.64 min (major), 3.77 min (minor), 92:8 er.

(3*R*,4*R*,5*R*)-Diethyl 5-(5-chloro-2-hydroxyphenyl)-4-(3-cyano-2-oxo-2*H*-chromen-4-yl)-3-phenylpyrrolidine-2,2-dicarboxylate **3n**

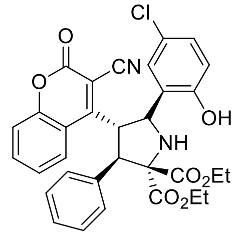

Following the general procedure, compound **3n** was isolated in 58% yield (34 mg) after 3 days as a colorless crystals. Mp = 112-113 °C; R_f = 0.22 (dichloromethane/acetone 100:1); $[\alpha]_D^{20}$= -3.5 (*c* 1, CH_2Cl_2); ^{1}H NMR (700 MHz, $CDCl_3$) δ (ppm) 10.75 (s, 1H), 7.61 (dd, J = 8.4 Hz, J = 1.3 Hz, 1H), 7.54 – 7.51 (m, 1H), 7.42 – 7.39 (m, 2H), 7.23 – 7.19 (m, 4H), 7.17 (ddd, J = 8.4 Hz, J = 5.4 Hz, J = 1.7 Hz, 1H), 7.03 – 7.01 (m, 1H), 6.89 (d, J = 8.7 Hz, 1H), 6.70 (d, J = 2.6 Hz, 1H), 5.49 (d, J = 11.9 Hz, 1H), 5.07 (dd, J = 10.4 Hz, J = 4.5 Hz, 1H), 5.06 – 5.02 (m, 1H), 4.48 (dq, J = 10.7 Hz, J = 7.2 Hz, 1H), 4.40 (dq, J = 10.7 Hz, J = 7.1 Hz, 1H), 3.87 – 3.82 (m, 2H), 3.61 (dq, J = 10.7 Hz, J = 7.2 Hz, 1H), 1.41 (t, J = 7.2 Hz, 3H), 0.80 (t, J = 7.2 Hz, 3H); ^{13}C NMR (176 MHz, $CDCl_3$) δ (ppm) 169.9, 169.8, 161.3, 156.8, 155.7, 153.0, 135.6, 134.4, 129.9, 128.7 (2C), 128.62 (2C), 128.57, 128.0, 125.6, 124.8, 124.4, 122.6, 119.5, 118.4, 117.8, 113.7, 101.2, 74.9, 65.1, 63.4, 62.7, 53.0, 46.4, 14.0, 13.3; HRMS (ESI) m/z: $[M+H]^+$ Calcd for $C_{32}H_{28}ClN_2O_7$ 587.1579; Found

587.1581; UPC[2] (Chiralpack IB, CO_2/*i*-PrOH gradient from 100% up to 40%, flow rate = 2.2 mL/min, λ = 225.7 nm) tR = 3.41 min (major), 3.28 min (minor), 94:6 er.

(3*R*,4*R*,5*S*)-Diethyl 4-(3-cyano-2-oxo-2*H*-chromen-4-yl)-5-(2-hydroxy-5-nitrophenyl)-3-phenylpyrrolidine-2,2-dicarboxylate **3o**

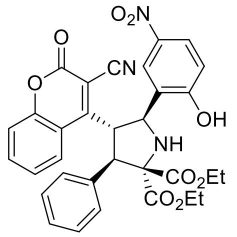

Following the general procedure, compound **3o** was isolated in 71% yield (42.4 mg) after 3 days as a colorless crystals. Mp = 100-102 °C; R_f = 0.39 (dichloromethane/acetone 100:1); $[\alpha]_D^{20}$= -2.6 (*c* 1, CH_2Cl_2); ^{1}H NMR (700 MHz, $CDCl_3$) δ (ppm) 11.89 (s, 1H), 7.99 (dd, *J* = 9.0 Hz, *J* = 2.8 Hz, 1H), 7.66 (d, *J* = 2.7 Hz, 1H), 7.54 – 7.51 (m, 1H), 7.51 – 7.49 (m, 1H), 7.41 – 7.39 (m, 2H), 7.25 – 7.20 (m, 4H), 7.15 (ddd, *J* = 8.4 Hz, *J* = 7.4 Hz, *J* = 1.2 Hz, 1H), 7.04 (d, *J* = 9.0 Hz, 1H), 5.54 (d, *J* = 12.6 Hz, 1H), 5.23 (dd, *J* = 10.6 Hz, *J* = 4.8 Hz, 1H), 4.98 (dd, *J* = 12.4 Hz, *J* = 10.7 Hz, 1H), 4.50 (dq, *J* = 10.7 Hz, *J* = 7.2 Hz, 1H), 4.41 (dq, *J* = 10.7 Hz, *J* = 7.1 Hz, 1H), 3.97 (d, *J* = 4.8 Hz, 1H), 3.87 (dq, *J* = 10.7 Hz, *J* = 7.2 Hz, 1H), 3.62 (dq, *J* = 10.7 Hz, *J* = 7.2 Hz, 1H), 1.42 (t, *J* = 7.2 Hz, 3H), 0.80 (t, *J* = 7.2 Hz, 3H); ^{13}C NMR (176 MHz, $CDCl_3$) δ (ppm) 169.9, 169.6, 163.1, 160.3, 156.4, 153.1, 140.4, 135.7, 133.9, 128.9, 128.8 (2C), 128.73, 128.68, 126.2, 125.6, 124.4, 124.2, 121.5, 118.6, 118.2, 118.1, 113.7, 101.5, 75.0, 64.9, 63.6, 62.9, 52.9, 46.6, 14.0, 13.3; HRMS (ESI) m/z: $[M+H]^+$ Calcd for $C_{32}H_{28}N_3O_9$ 598.1820; Found 598.1817; UPC[2] (Chiralpack IB, CO_2/*i*-PrOH gradient from 100% up to 40%, flow rate = 2.2 mL/min, λ = 227.5 nm) tR = 3.33 min (major), 3.24 min (minor), 98:2 er.

(3*R*,4*R*,5*S*)-Diethyl 4-(3-cyano-2-oxo-2*H*-chromen-4-yl)-5-(2-hydroxy-5-methylphenyl)-3-phenylpyrrolidine-2,2-dicarboxylate **3p**

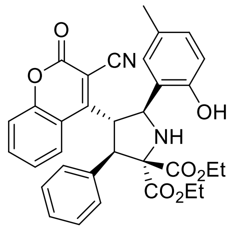

Following the general procedure, compound **3p** was isolated in 54% yield (30.6 mg) after 3 days as a yellowish solid. Mp = 92-93 °C; R_f = 0.4 (dichloromethane/acetone 100:1); $[\alpha]_D^{19}$= -4.3 (*c* 1, CH_2Cl_2); ^{1}H NMR (400 MHz, $CDCl_3$) δ (ppm) 10.45 (s, 1H),7.66 (d, *J* = 1.3 Hz), 7.50 (ddd, *J* = 8.3 Hz, *J* = 7.4 Hz, *J* = 1.4 Hz, 1H), 7.43 – 7.41 (m, 2H), 7.22 – 7.14 (m, 5H), 6.85 – 6.83 (m, 2H), 6.50 (d, *J* = 1.7 Hz, 1H), 5.51 (d, *J* = 12.1 Hz, 1H), 5.11 – 5.03 (m, 2H), 4.47 (dq, *J* = 10.7 Hz, *J* = 7.2 Hz, 1H), 4.40 (dq, *J* = 10.7 Hz, *J* = 7.2 Hz, 1H), 3.84 (dq, *J* = 10.7 Hz, *J* = 7.2 Hz, 1H), 3.80 (d, *J* = 3.7 Hz, 1H), 3.61 (dq, *J* = 10.7Hz, *J* = 7.2 Hz, 1H), 2.00 (s, 3H), 1.40 (t, *J* = 7.2 Hz, 3H), 0.81 (t, *J* = 7.2 Hz, 3H); ^{13}C NMR (101 MHz, $CDCl_3$) δ (ppm) 170.1, 169.9, 162.0, 157.1, 154.6, 152.9, 135.3, 134.7, 130.6, 128.9, 128.81, 128.77 (2C), 128.5 (2C), 128.4, 125.5, 125.2, 120.6, 118.6, 117.9, 117.5, 113.8, 101.0, 75.0, 65.7, 63.2, 62.5, 53.0, 46.5, 20.2, 14.0, 13.3; HRMS (ESI) m/z: $[M+H]^+$ Calcd for $C_{33}H_{31}N_2O_7$ 567.2125; Found 567.2127; UPC[2] (Chiralpack IA, CO_2/*i*-PrOH gradient from 100% up to 40%, flow rate = 2.2 mL/min, λ = 228.2 nm) tR = 3.81 min (major), 3.58 min (minor), 94:6 er.

(3*R*,4*R*,5*S*)-Diethyl 5-(5-(*tert*-butyl)-2-hydroxyphenyl)-4-(3-cyano-2-oxo-2*H*-chromen-4-yl)-3-phenylpyrrolidine-2,2-dicarboxylate **3r**

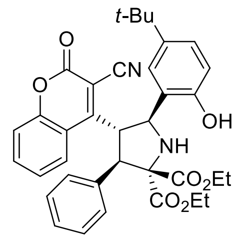

Following the general procedure, compound **3r** was isolated in 46% yield (28 mg) after 4 days as a yellowish solid. Mp = 88 °C; R_f = 0.38 (dichloromethane/acetone 100:1); $[\alpha]_D^{22}$= -3.4 (*c* 1, CH_2Cl_2); ^1H NMR (700 MHz, $CDCl_3$) δ (ppm) 10.73 (s, 1H), 7.49 – 7.46 (m, 2H), 7.46 – 7.43 (m, 2H), 7.24 – 7.18 (m, 3H), 7.14 (dd, *J* = 8.2 Hz, *J* = 1.0 Hz, 1H), 7.07 (ddd, *J* = 8.5 Hz, *J* = 7.4 Hz, *J* = 1.2 Hz, 1H), 6.99 (dd, *J* = 8.5 Hz, *J* = 2.5 Hz, 1H), 6.85 (d, *J* = 8.5 Hz, 1H), 6.50 (d, *J* = 2.4 Hz, 1H), 5.65 (d, *J* = 12.3 Hz, 1H), 5.00 (dd, *J* = 10.5 Hz, *J* = 4.7 Hz, 1H), 4.95 (dd, *J* = 12.2 Hz, *J* = 10.8 Hz, 1H), 4.48 (dq, *J* = 10.7 Hz, *J* = 7.2 Hz, 1H), 4.41 (dq, *J* = 10.7 Hz, *J* = 7.1 Hz, 1H), 3.89 (d, *J* = 4.8 Hz, 1H), 3.85 (dq, *J* = 10.7 Hz, *J* = 7.1 Hz, 1H), 3.63 (dq, *J* = 10.7 Hz, *J* = 7.2 Hz, 1H), 1.42 (t, *J* = 7.2 Hz, 3H), 0.90 (s, 9H), 0.82 (t, *J* = 7.2 Hz, 3H); ^{13}C NMR (176 MHz, $CDCl_3$) δ (ppm) 170.1, 169.7, 161.9, 156.9, 154.6, 152.8, 142.3, 135.1, 134.9, 128.8 (2C), 128.6 (2C), 128.4, 126.4, 125.4, 125.2, 124.7, 119.9, 118.5, 117.4, 117.3, 113.8, 100.7, 75.3, 66.9, 63.3, 62.5, 52.1, 47.6, 33.5, 31.1 (3C), 14.0, 13.4; HRMS (ESI) m/z: $[M+H]^+$ Calcd for $C_{36}H_{37}N_2O_7$ 609.2595; Found 609.2596; UPC^2 (Chiralpack IA, CO_2/*i*-PrOH gradient from 100% up to 40%, flow rate = 2.2 mL/min, λ = 227.8 nm) tR = 3.64 min (major), 3.33 min (minor), 97:3 er.

(3*R*,4*R*,5*S*)-Diethyl 4-(3-cyano-2-oxo-2*H*-chromen-4-yl)-5-(2-hydroxy-4,6-dimethoxy-phenyl)-3-phenyl-pyrrolidine-2,2-dicarboxylate **3s**

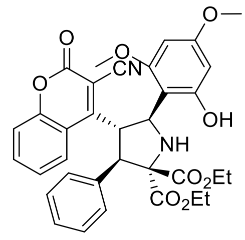

Following the general procedure, compound **3s** was isolated in 84% yield (51.5 mg) after 3 days as a yellowish solid. Mp = 95-97 °C; R_f = 0.42 (dichloromethane/acetone 100:1); $[\alpha]_D^{21}$= -8.2 (*c* 1, CH_2Cl_2); ^1H NMR (400 MHz, $CDCl_3$) δ (ppm) 11.51 (s, 1H), 7.63 (dd, *J* = 8.7 Hz, *J* = 0.9 Hz, 1H), 7.52 – 7.49 (m, 1H), 7.43 – 7.41 (m, 2H), 7.22 – 7.17 (m, 4H), 7.17 – 7.14 (m, 1H), 6.11 (d, *J* = 2.4 Hz, 1H), 5.81 (dd, *J* = 10.6 Hz, *J* = 5.1 Hz, 1H), 5.63 (d, *J* = 2.4 Hz, 1H), 5.51 (d, *J* = 12.5 Hz, 1H), 4.86 (dd, *J* = 12.7 Hz, *J* = 10.8 Hz, 1H), 4.48 (ddd, *J* = 14.3 Hz, *J* = 10.7 Hz, *J* = 7.2 Hz, 1H), 4.40 (dq, *J* = 10.7 Hz, *J* = 7.1 Hz, 1H), 3.84 (dq, *J* = 10.7 Hz, *J* = 7.2 Hz, 1H), 3.68 (m, 4H), 3.62 (dq, *J* = 14.4 Hz, *J* = 7.2 Hz, 1H), 3.44 (s, 3H), 1.41 (t, *J* = 7.2 Hz, 3H), 0.82 (t, *J* = 7.2 Hz, 3H); ^{13}C NMR (101 MHz, $CDCl_3$) δ (ppm) 170.1, 169.6, 161.5, 161.3, 159.6, 158.2, 157.1, 152.8, 134.97, 134.96, 128.8 (2C), 128.5 (2C), 128.3, 125.3, 125.2, 118.6, 117.4, 113.8, 101.5, 101.4, 95.0, 90.1, 75.1, 63.3, 62.4, 57.2, 55.2, 55.1, 52.8, 47.1, 14.0, 13.3; HRMS (ESI) m/z: $[M+H]^+$ Calcd for $C_{34}H_{33}N_2O_9$ 613.2180; Found 613.2179; UPC^2 (Chiralpack IC, CO_2/*i*-PrOH gradient from 100% up to 40%, flow rate = 2.2 mL/min, λ = 227.3 nm) tR = 4.20 min (major), 4.53 min (minor), 94.5:5.5 er.

3. Enatioselective synthesis of (3*R*,4*R*,5*S*)-Diethyl 4-(3-cyano-2-oxo-2*H*-chromen-4-yl)-5-(2-hydroxyphenyl)-3-phenyl-pyrrolidine-2,2-dicarboxylate 3a on a 1mmol scale

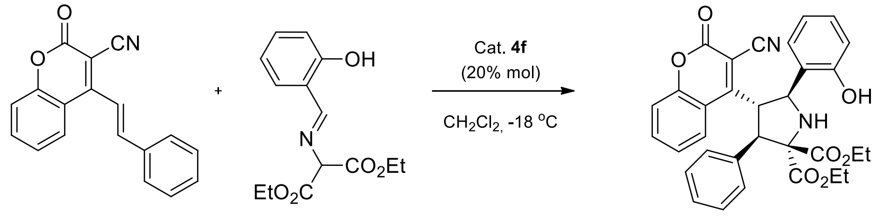

Scheme 2.

In an ordinary 4 mL glass vial, equipped with a magnetic stirring bar and a screw cap, 4-(alk-1-en-1-yl)-3-cyanocoumarin **1a** (1 equiv., 1 mmol, 273,3 mg) and aldimine **2a** (1.2 equiv., 1.2 mmol, 336 mg) were dissolved in dichloromethane (2 mL). After cooling to –18 °C catalyst **4f** (20 mol%, 0.2 mmol, 126 mg) was added and reaction was stirred for 72 hours at –18 °C. The reaction mixture was purified by the column chromatography on silica gel using dichloromethane/acetone 100:1 as an eluent to obtain pure product **3a** in 78% (430 mg) yield as a white solid. Mp = 92 °C; $[\alpha]_D^{21}$= -6.0 (*c* 1, CH_2Cl_2); HRMS (ESI) m/z: $[M+H]^+$ Calcd. for $C_{32}H_{29}N_2O_7$ 553.1969; Found 553.1975; UPC^2 (Chiralpack IC, CO_2/*i*-PrOH gradient from 100% up to 40%, flow rate = 2.2 mL/min, λ = 226.4nm) tR = 4.22 min (major), 4.63 min (minor), 92:8 er. Spectral data were in accordance with the data reported above.

4. Synthesis of compounds 5 – general procedure

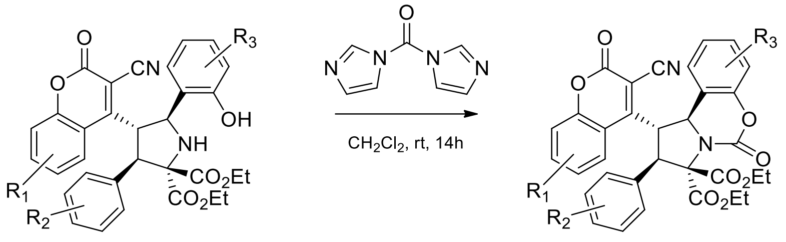

Scheme 3.

To the solution of **3** (1 equiv., 0.1mmol) in dichloromethane (0.7 mL) *N*,*N*'-carbonyldiimidazole (1 equiv., 0.1 mmol) was added and the reaction was allowed to procced overnight. The crude product **5** purified by column chromatography od silica gel using hexane/ethyl acetate 2:1 as an eluent.

(1*R*,2*R*,10b*S*)-Diethyl 1-(3-cyano-2-oxo-2*H*-chromen-4-yl)-5-oxo-2-phenyl-5,10*b*-dihydro-1*H*-benzo-[*e*]pyrrolo[1,2-*c*][1,3]oxazine-3,3(2*H*)-dicarboxylate **5a**

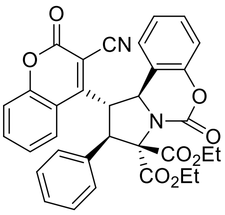

Following the general procedure, compound **5a** was isolated in 65% yield (37.6 mg) after 14 hours as a colorless crystals (inseparable mixture with circa 10% of 2nd diastereoisomer). Mp = 116-118 °C; R_f = 0.4 (hexane/ethyl acetate 1.5:1); $[\alpha]_D^{18}$= +17.7 (*c* 1, CH_2Cl_2); Major diastereoisomer: ^{1}H NMR (700 MHz, $CDCl_3$) δ (ppm) 7.88 (d, *J* = 7.8 Hz, 1H), 7.74 (ddd, *J* = 8.5 Hz, *J* = 7.4 Hz, *J* = 1.4 Hz, 1H), 7.43 – 7.39 (m, 2H), 7.31 – 7.22 (m, 4H), 7.18 – 7.15 (m, 3H), 6.84 (td, *J* = 7.6 Hz, *J* = 1.1 Hz, 1H), 6.43 (d, *J* = 7.7 Hz, 1H), 6.03 (d, *J* = 10.3 Hz, 1H), 5.10 (d, *J* = 12.4 Hz, 1H), 4.83 (dd, *J* = 12.4 Hz, *J* = 10.4 Hz, 1H), 4.48 – 4.44 (m, 2H), 3.99 (dq, *J* = 10.8 Hz, *J* = 7.1 Hz, 1H), 3.87 (ddd, *J* = 14.2 Hz, *J* = 10.7 Hz, *J* = 7.1 Hz, 1H), 1.42 (t, *J* = 7.1 Hz, 3H), 0.96 (t, *J* = 7.1 Hz, 3H); Minor diastereoisomer: ^{1}H NMR (700 MHz, $CDCl_3$) δ (ppm) 8.20 (d, *J* = 8.2 Hz), 7.78 – 7.76 (m, 1H), 7.52 – 7.47 (m), 7.23 (d, *J* = 8.7 Hz), 7.21 – 7.18 (m), 6.89 (d, *J* = 7.7 Hz), 5.98 (d, *J* = 9.5 Hz), 4.99 – 4.86 (m), 4.45 – 4.43 (m), 4.03 – 3.92 (m), 3.90 – 3.81 (m), 1.42 (t, *J* = 7.2 Hz), 0.99 (t, *J* = 7.2 Hz); Major diastereoisomer: ^{13}C NMR (176 MHz, $CDCl_3$) δ (ppm) 166.4, 165.4, 160.0, 156.3, 153.5, 149.2, 147.8, 131.6, 130.3, 129.5 (2C), 129.1, 128.9, 128.5 (2C), 125.9, 125.8, 124.6, 123.9, 118.5, 118.4, 117.9, 117.5, 74.8, 63.0, 62.7, 59.5, 55.6, 47.6, 14.1, 13.4; Minor diastereoisomer: ^{13}C NMR (176 MHz, $CDCl_3$) δ (ppm) 168.6, 164.6, 163.8, 159.1, 155.6, 153.9, 149.3, 148.4, 135.6, 130.4, 129.6, 128.9 (2C), 125.7, 125.6, 125.4, 123.1, 120.5, 119.4, 117.2, 115.3, 113.3, 106.6, 75.6, 63.3, 62.6, 59.9, 57.9, 54.8, 14.1, 13.4; HRMS (ESI) m/z: $[M+H]^+$ Calcd for $C_{33}H_{26}N_2O_8$ 579.1762; Found 579.1769; UPC^2 (Chiralpack IB, CO_2/*i*-PrOH gradient from 100% up to 40%, flow rate = 2.2 mL/min, λ = 228.2 nm) tR = 4.10 min (major), 3.74 min (minor), 85:15 er.

(1R,2R,10bS)-Diethyl 2-(4-chlorophenyl)-1-(3-cyano-2-oxo-2H-chromen-4-yl)-5-oxo-5,10b-dihydro-1H-benzo[e]pyrrolo[1,2-c][1,3]oxazine-3,3(2H)-dicarboxylate **5b**

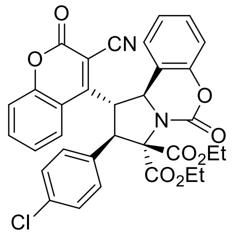

Following the general procedure, compound **5b** was isolated in 51% yield (31.3 mg) after 14 hours as a colorless crystals (inseparable mixture with circa 10% of 2nd diastereoisomer). Mp = 116-118 °C; R_f = 0.41 (hexane/ethyl acetate 1.5:1); $[\alpha]_D^{23}$= +18.7 (c 1, CH_2Cl_2); Major diastereoisomer: ^{1}H NMR (700 MHz, $CDCl_3$) δ (ppm) 7.91 (d, J = 7.8 Hz, 1H), 7.77 (ddd, J = 8.5 Hz, J = 7.5 Hz, J = 1.3 Hz, 1H), 7.45 – 7.41 (m, 2H), 7.34 – 7.30 (m, 3H), 7.20 – 7.18 (m, 3H), 6.87 (td, J = 7.6 Hz, J = 1.1 Hz, 1H), 6.45 (d, J = 7.7 Hz, 1H), 6.06 (d, J = 10.3 Hz, 1H), 5.13 (d, J = 12.4 Hz, 1H), 4.85 (dd, J = 12.4 Hz, J = 10.4 Hz, 1H), 4.51 – 4.46 (m, 2H), 4.02 (dq, J = 10.8 Hz, J = 7.1 Hz, 1H), 3.89 (ddd, J = 14.3 Hz, J = 10.8 Hz, J = 7.1 Hz, 1H), 1.44 (t, J = 6.3 Hz, 3H), 0.98 (t, J = 7.1 Hz, 3H); Minor diastereoisomer: ^{1}H NMR (700 MHz, $CDCl_3$) δ (ppm) 8.23 (d, J = 8.1 Hz), 7.81 – 7.78 (m), 7.56 – 7.49 (m), 7.36 – 7.31 (m), 7.27 – 7.20 (m), 6.91 (d, J = 7.7 Hz), 6.00 (d, J = 9.5 Hz), 4.96 – 4.89 (m), 4.54 – 4.49 (m), 4.05 – 3.96 (m), 3.93 – 3.85 (m), 1.45 (t, J = 7.3 Hz), 1.01 (t, J = 7.1 Hz).Major distereoisomer: ^{13}C NMR (176 MHz, $CDCl_3$) δ (ppm) 166.6, 165.6, 159.9, 156.4, 153.7, 149.3, 147.8, 136.5, 135.4, 131.1, 130.6, 130.3, 129.4, 128.9 (2C), 126.1, 125.8, 124.8, 124.2, 118.8, 118.3, 117.9, 117.7, 113.6, 101.9, 74.9, 63.4, 63.1, 59.7, 55.0, 47.9, 14.3, 13.7; Minor distereoisomer: ^{13}C NMR (176 MHz, $CDCl_3$) δ (ppm) 168.6, 164.0, 159.0, 155.7, 154.2, 149.4, 136.0, 135.9, 130.7 (2C), 130.6, 130.1, 126.0, 125.6 (2C), 123.2, 120.3, 119.7, 117.4, 115.3, 113.5, 106.8, 75.7, 63.6, 63.0, 60.1, 57.2, 55.1, 14.3, 13.7; HRMS (ESI) m/z: $[M+H]^+$ Calcd for $C_{33}H_{26}ClN_2O_8$ 613.1372; Found 613.1376; UPC^2 (Chiralpack IC, CO_2/i-PrOH gradient from 100% up to 40%, flow rate = 2.2 mL/min, λ = 228.1 nm) tR = 4.17 min (major), 3.74 min (minor), 86.5:13.5 er.

(1R,2R,10bS)-Diethyl 7-bromo-1-(3-cyano-2-oxo-2H-chromen-4-yl)-5-oxo-2-phenyl-5,10b-dihydro-1H-benzo[e]pyrrolo[1,2-c][1,3]oxazine-3,3(2H)-dicarboxylate **5c**

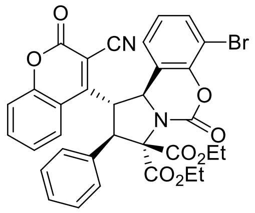

Following the general procedure, compound **5c** was isolated in 61% yield (40 mg) after 14 hours as a colorless crystals (inseparable mixture with circa 10% of 2nd diastereoisomer). Mp = 116-117 °C; R_f = 0.41 (hexane/ethyl acetate 1.5:1); $[\alpha]_D^{22}$= +11.9 (c 1, CH_2Cl_2); Major diastereoisomer: ^{1}H NMR (700 MHz, $CDCl_3$) δ (ppm) 7.92 (d, J = 7.8 Hz, 1H), 7.74 – 7.71 (m, 1H), 7.56 – 7.53 (m, 1H), 7.43 – 7.37 (m, 2H), 7.33 – 7.30 (m, 2H), 7.22 – 7.16 (m, 3H), 6.72 (t, J = 7.9 Hz, 1H), 6.41 – 6.36 (m, 1H), 6.08 (d, J = 10.3 Hz, 1H), 5.12 (d, J = 12.4 Hz, 1H), 4.89 (dd, J = 12.4 Hz, J = 10.4 Hz, 1H), 4.50 – 4.41 (m, 2H), 3.96 (dq, J = 10.8 Hz, J = 7.2 Hz, 1H), 3.80 (dq, J = 10.8, 7.1 Hz, 1H), 1.42 (t, J = 7.1 Hz, 3H), 0.89 (t, J = 7.1 Hz, 3H); Minor diastereoisomer: ^{1}H NMR (700 MHz, $CDCl_3$) δ (ppm) 8.27 (d, J = 8.3 Hz), 7.77 (ddd, J = 8.5, 7.4, 1.3 Hz), 7.64 – 7.60 (m), 7.51 (td, J = 7.9, 1.3 Hz), 7.47 (dd, J = 8.4, 1.2 Hz), 7.32 (d, J = 2.2 Hz), 7.23 – 7.21 (m), 7.06 (t, J = 7.9 Hz), 6.89 (dt, J = 7.7, 1.2 Hz), 6.01 (d, J = 9.4 Hz), 4.99 – 4.92 (m), 4.52 – 4.42 (m), 4.00 – 3.91 (m), 3.75 (dq, J = 10.8, 7.2 Hz), 1.42 (t, J = 7.1 Hz), 0.92 (t, J = 7.1 Hz); Major diastereoisomer: ^{13}C NMR (176 MHz, $CDCl_3$) δ (ppm) 166.2, 165.3, 159.7, 156.2, 153.5, 146.8, 146.5, 136.2, 134.2, 131.3, 129.5 (2C), 129.2, 128.5 (2C), 125.84, 125.80, 125.2, 122.9, 120.1, 118.6, 117.8, 113.3, 111.5, 101.9, 74.7, 63.1, 62.8, 59.5, 55.8, 47.4, 14.0, 13.5; Minor diastereoisomer: ^{13}C NMR (176 MHz, $CDCl_3$) δ (ppm) 168.4, 163.8, 158.9, 155.6, 154.0, 147.5, 146.7, 135.7, 134.2, 131.3, 129.6, 129.1, 129.0 (2C), 126.1, 125.6, 122.3, 122.1, 119.5, 115.1, 113.3, 111.2, 106.6,

75.6, 63.4, 62.7, 60.0, 58.0, 54.4, 14.1, 13.4; HRMS (ESI) m/z: $[M+H]^+$ Calcd for $C_{33}H_{26}BrN_2O_8$ 657.0867; Found 657.0866; UPC^2 (Chiralpack IA, CO_2/*i*-PrOH gradient from 100% up to 40%, flow rate = 2.2 mL/min, λ = 228.2 nm) tR = 4.10 min (major), 4.41 min (minor), 90:10 er.

5. Crystal and X-ray data for (1*R*,2*R*,10b*S*)-diethyl 1-(3-cyano-2-oxo-2*H*-chromen-4-yl)-5-oxo-2-phenyl-5,10*b*-dihydro-1*H*-benzo[*e*]pyrrolo[1,2*c*][1,3]oxazine-3,3(2*H*)-dicarboxylate (5a)

Suitable crystals of compound **5a** were obtained by slowly evaporating a mixture of cyclohexane and ethyl acetate solution at 5 °C. The crystal structure of the compound **5a**, ($C_{33}H_{26}N_2O_8 \cdot C_4H_8O_2$), was established by single-crystal X-ray diffraction at 100 K. The compound crystallizes in the non-centrosymmetric orthorhombic space group $Pna2_1$ (Z = 4) and the crystal structure consists of one crystallographically independent main molecule (Figure 1) and one ethyl acetate solvent molecule in the unit cell.

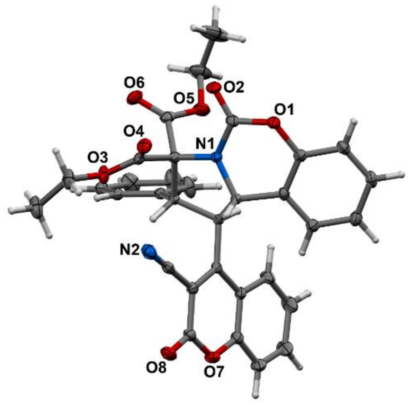

Figure 1. View of a main molecule of the compound **5a** at 100 K, showing 50% probability displacement ellipsoids. Hydrogen atoms are drawn with an arbitrary radius. Disordered solvent molecule was omitted for clarity.

Single crystal X-ray diffraction data were collected at 100 K by the ω-scan technique using a RIGAKU XtaLAB Synergy, Dualflex, Pilatus 300K diffractometer[4] with PhotonJet micro-focus X-ray Source Cu-Kα (λ = 1.54184 Å). Data collection, cell refinement, data reduction and absorption correction were performed using CrysAlis PRO software[4] . The crystal structure was solved by using direct methods with the SHELXT 2018/2 program[5] . Atomic scattering factors were taken from the International Tables for X-ray Crystallography. Positional parameters of non-H-atoms were refined by a full-matrix least-squares method on F^2 with anisotropic thermal parameters by using the SHELXL 2018/3 program[6] . All hydrogen atoms were found from the difference Fourier maps and for further calculations they were positioned geometrically in calculated positions (C–H = 0.95–1.00 Å) and constrained to ride on their parent atoms with isotropic displacement parameters set to 1.2-1.5 times the U_{eq} of the parent atom. The ethyl acetate solvent molecule was refined as disordered over two sets of sites with an occupancy ratio of 0.627(6):0.373(6).

5a: Formula $C_{37}H_{34}N_2O_{10}$, orthorhombic, space group $Pna2_1$, Z = 4, unit cell constants a = 18.3194(1), b = 12.2475(1), c = 14.6653(1) Å, V = 3290.41(4) Å^3. The integration of the data yielded a total of 109293 reflections with θ angles in the range of 4.34 to 79.22°, of which 6415 were unique (R_{int} = 2.86%). The final anisotropic full-matrix least-squares refinement on F^2 with 504 parameters. The final R_1 was 0.0244 (for $I > 2\sigma(I)$) and wR_2 was 0.0639 (all data). The largest peak in the final difference electron density synthesis was 0.216 eÅ^{-3} and the largest hole was -0.165 eÅ^{-3}. The goodness-of-fit was 1.028. The absolute configuration was unambiguously established from anomalous scattering, by calculating the x Flack parameter[7] of -0.016(18) using 2765 quotients.

CCDC 2226810 contains the supplementary crystallographic data for this paper. These data can be obtained free of charge from The Cambridge Crystallographic Data Centre via www.ccdc.cam.ac.uk/structures

4. Rigaku, O.D. CrysAlis PRO. Rigaku Oxford Diffraction Ltd, Yarnton, Oxfordshire, England, **2019**.
5. Sheldrick, G.M. SHELXT - integrated space-group and crystal-structure determination, *Acta Cryst.* **2015**, *A71*, 3-8.
6. Sheldrick, G.M. Crystal structure refinement with SHELXL, *Acta Cryst.* **2015**, *C71*, 3-8.
7. Parsons, S.; Flack, H.D.; Wagner, T. Use of intensity quotients and differences in absolute structure refinement, *Acta Cryst.* **2013**, *B69*, 249-259.

5. NMR data

(3*R*,4*R*,5*S*)-Diethyl 4-(3-cyano-2-oxo-2*H*-chromen-4-yl)-5-(2-hydroxyphenyl)-3-phenylpyrrolidine-2,2-dicarboxylate **3a**

^{1}H NMR (700 MHz, $CDCl_3$)

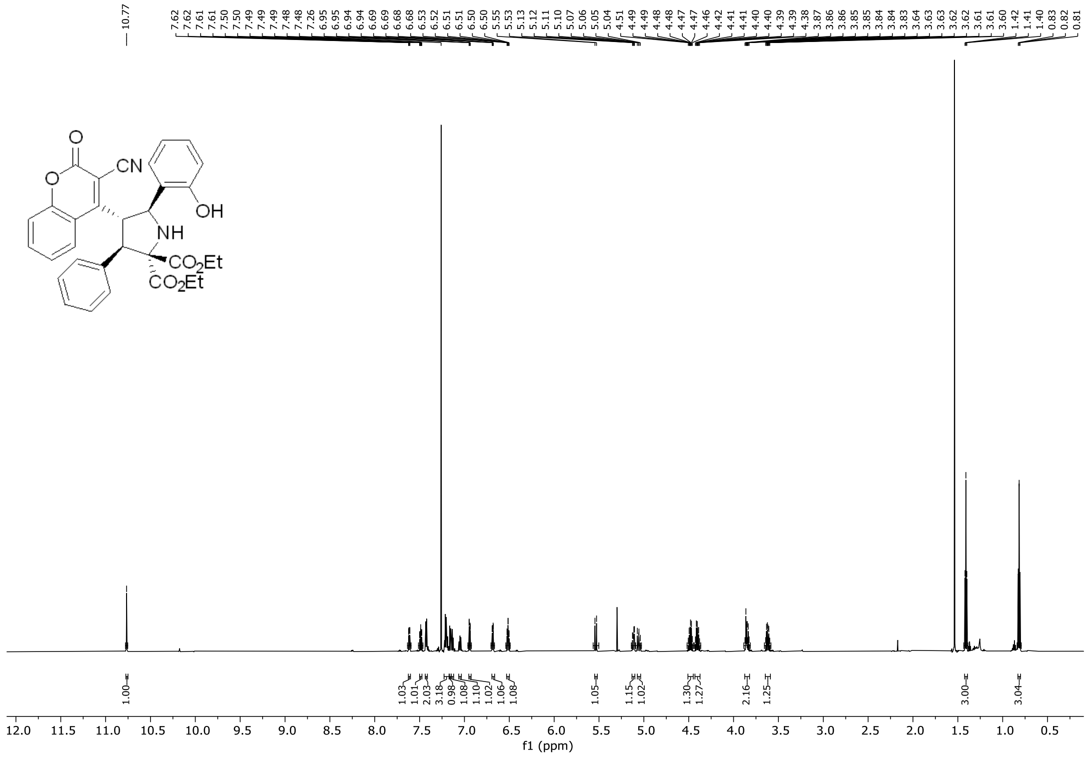

^{13}C NMR (176 MHz, $CDCl_3$)

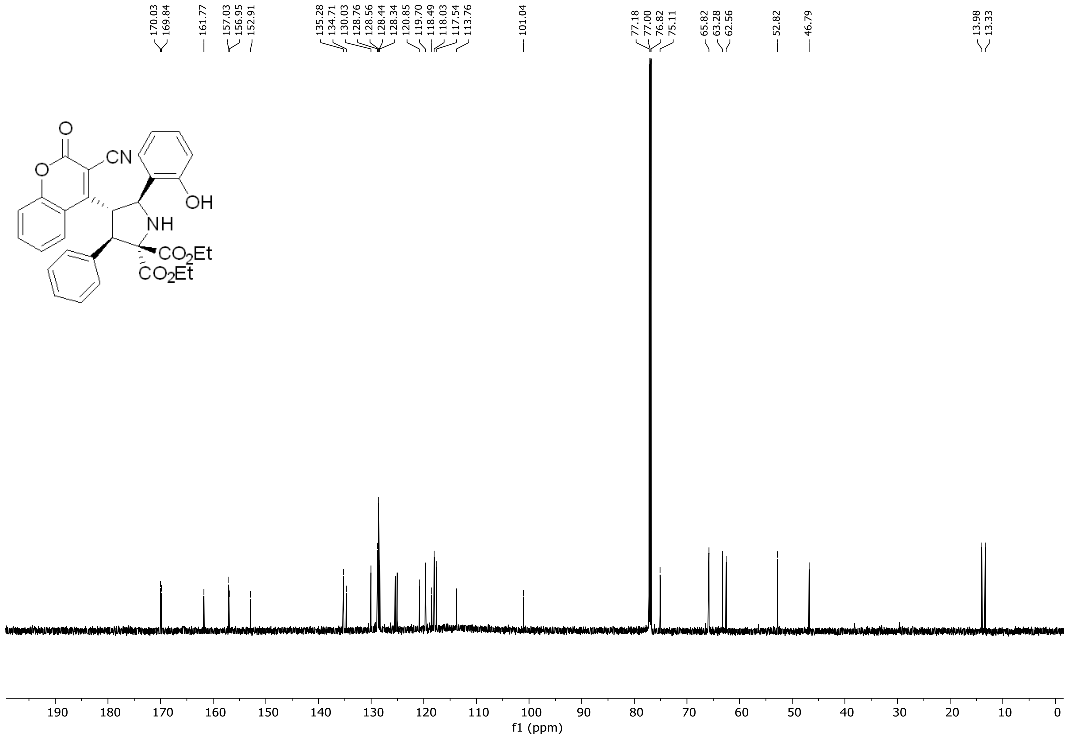

(3*R*,4*R*,5*S*)-Diethyl 3-(4-chlorophenyl)-4-(3-cyano-2-oxo-2*H*-chromen-4-yl)-5-(2-hydroxyphenyl)pyrolidine-2,2-dicarboxylate **3b**

^{1}H NMR (700 MHz, $CDCl_3$)

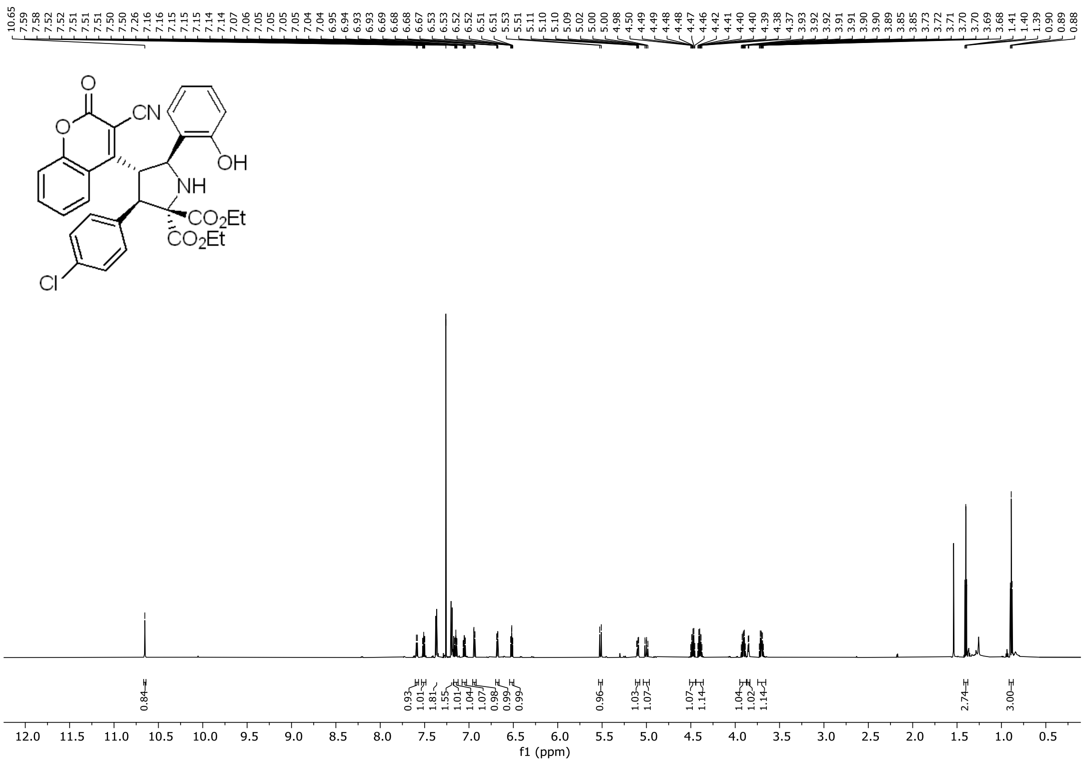

^{13}C NMR (176 MHz, $CDCl_3$)

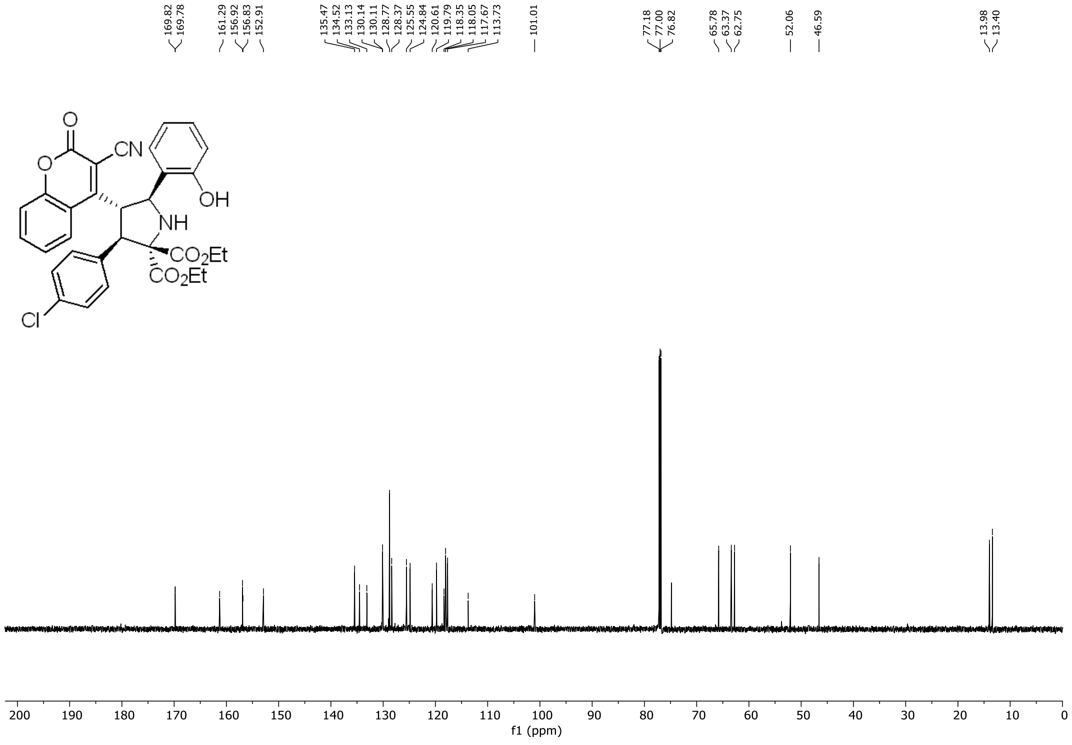

(3*R*,4*R*,5*S*)-Diethyl 3-(3-chlorophenyl)-4-(3-cyano-2-oxo-2*H*-chromen-4-yl)-5-(2-hydroxyphenyl)pyrolidine-2,2-dicarboxylate **3c**

^{1}H NMR (700 MHz, $CDCl_3$)

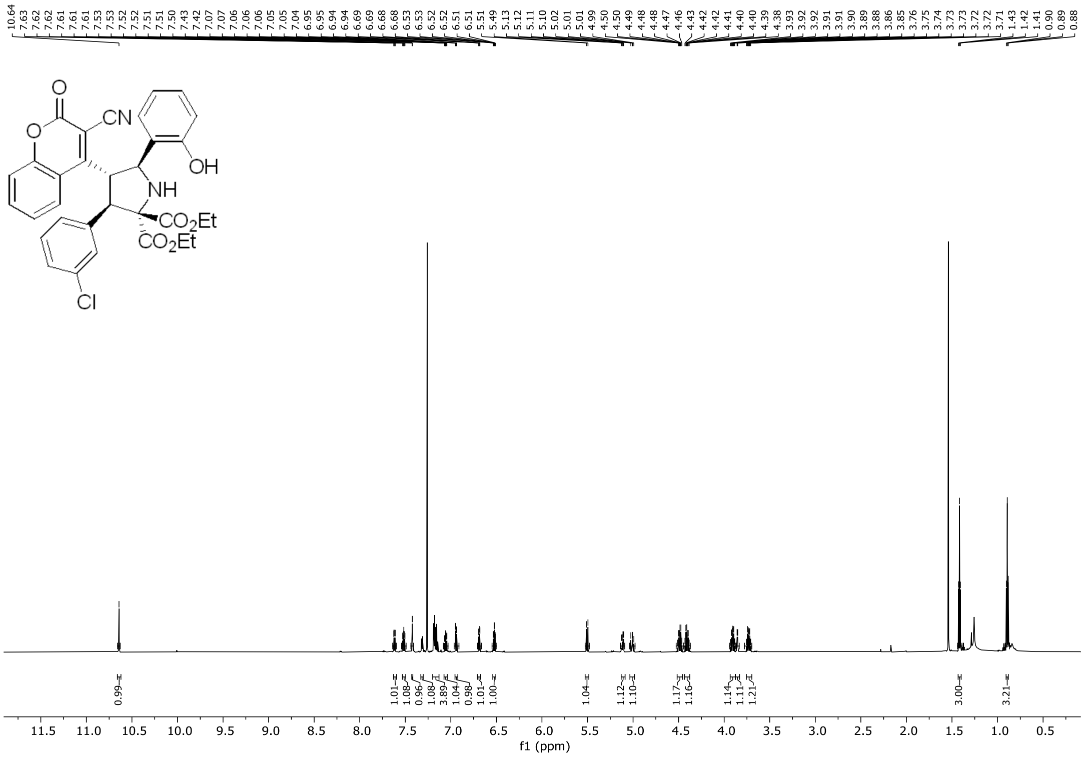

^{13}C NMR (176 MHz, $CDCl_3$)

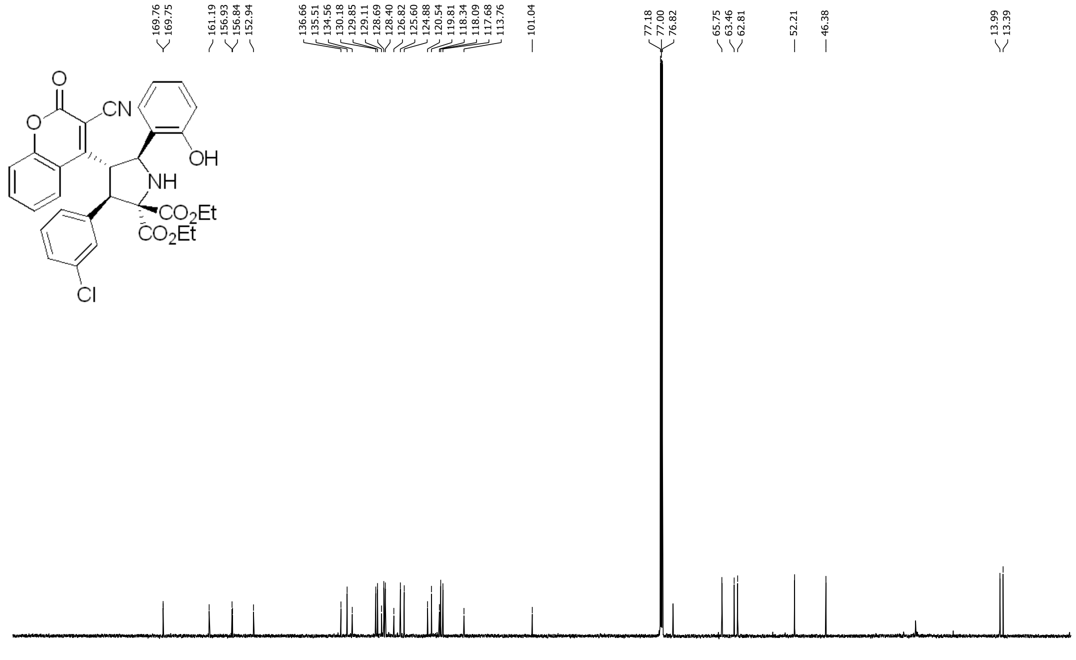

(3*R*,4*R*,5*S*)-Diethyl 4-(3-cyano-2-oxo-2*H*-chromen-4-yl)-3-(3-fluorophenyl)-5-(2-hydroxyphenyl)pyrolidine-2,2-dicarboxylate **3d**

^{1}H NMR (400 MHz, $CDCl_3$)

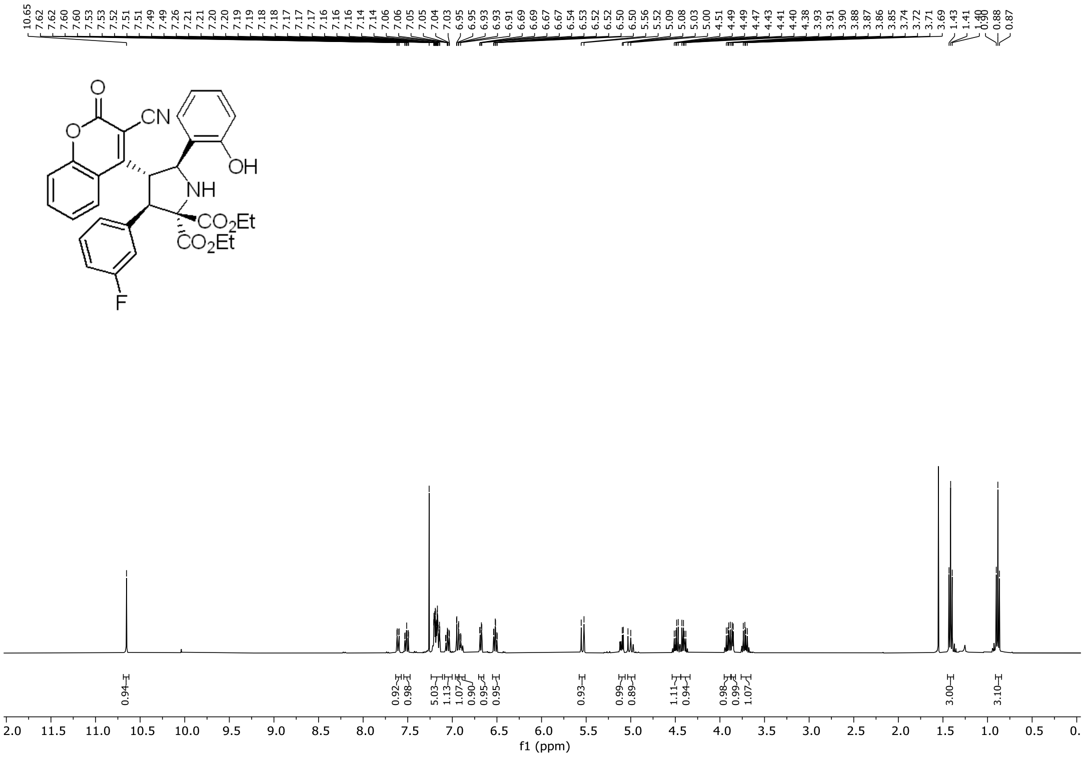

^{13}C NMR (101 MHz, $CDCl_3$)

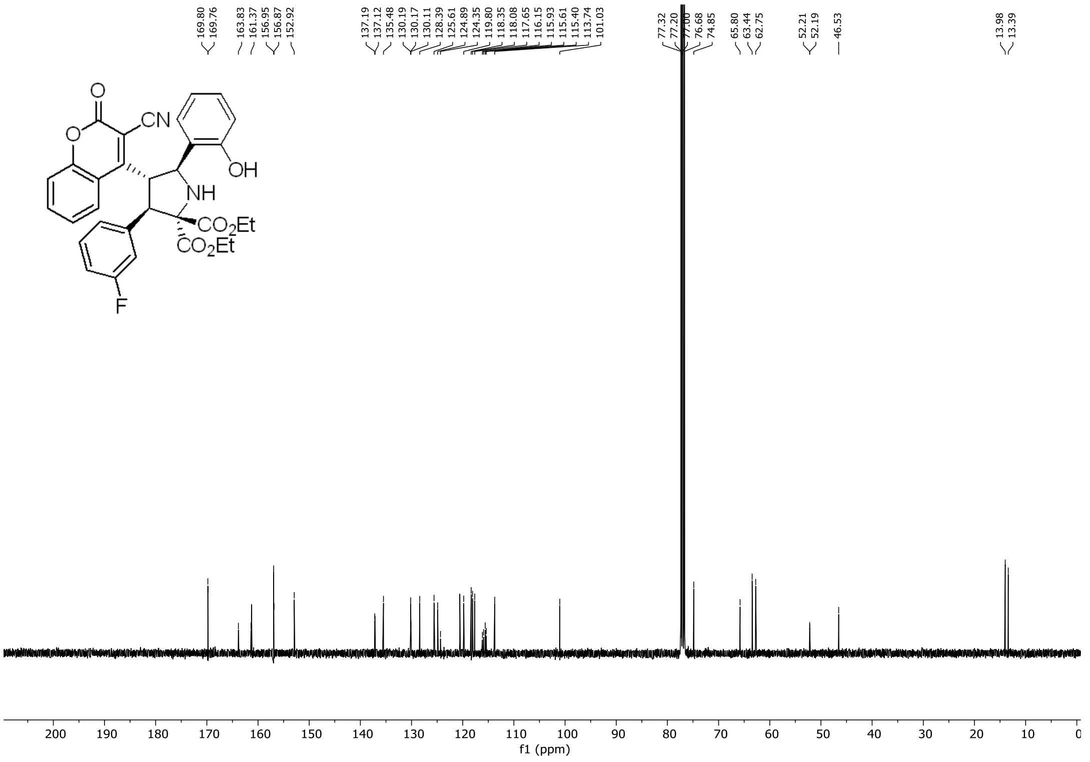

(3*R*,4*R*,5*S*)-Diethyl 4-(3-cyano-2-oxo-2*H*-chromen-4-yl)-5-(2-hydroxyphenyl)-3-(4-(trifluoro-methyl)phenyl)pyrolidine-2,2-dicarboxylate **3e**

^{1}H NMR (700 MHz, $CDCl_3$)

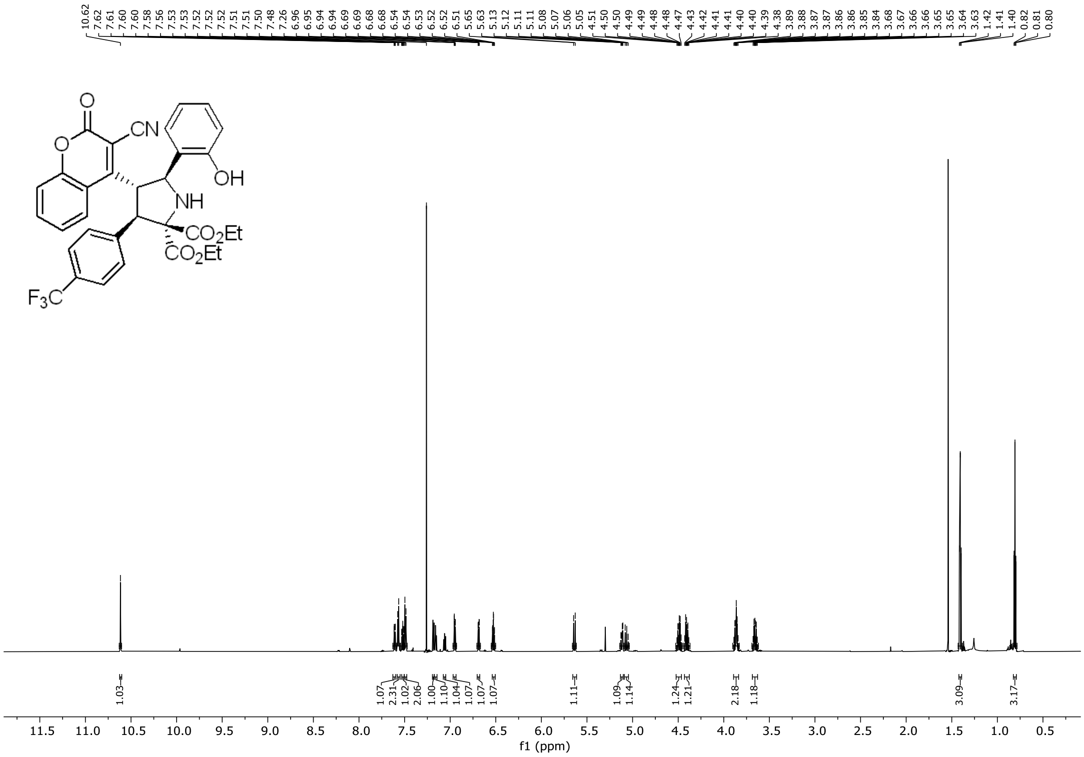

^{13}C NMR (176 MHz, $CDCl_3$)

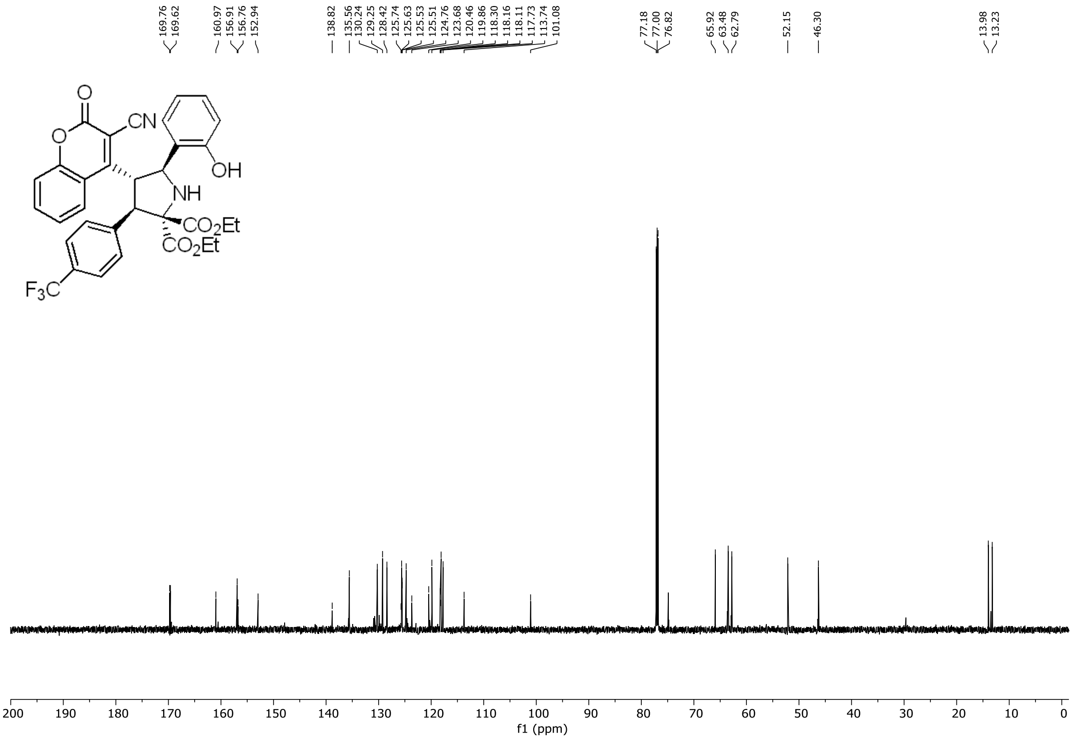

(3*R*,4*R*,5*S*)-Diethyl 4-(3-cyano-2-oxo-2*H*-chromen-4-yl)-5-(2-hydroxyphenyl)-3-(4-nitrophenyl)-pyrrolidine-2,2-dicarboxylate **3f**

^{1}H NMR (700 MHz, $CDCl_3$)

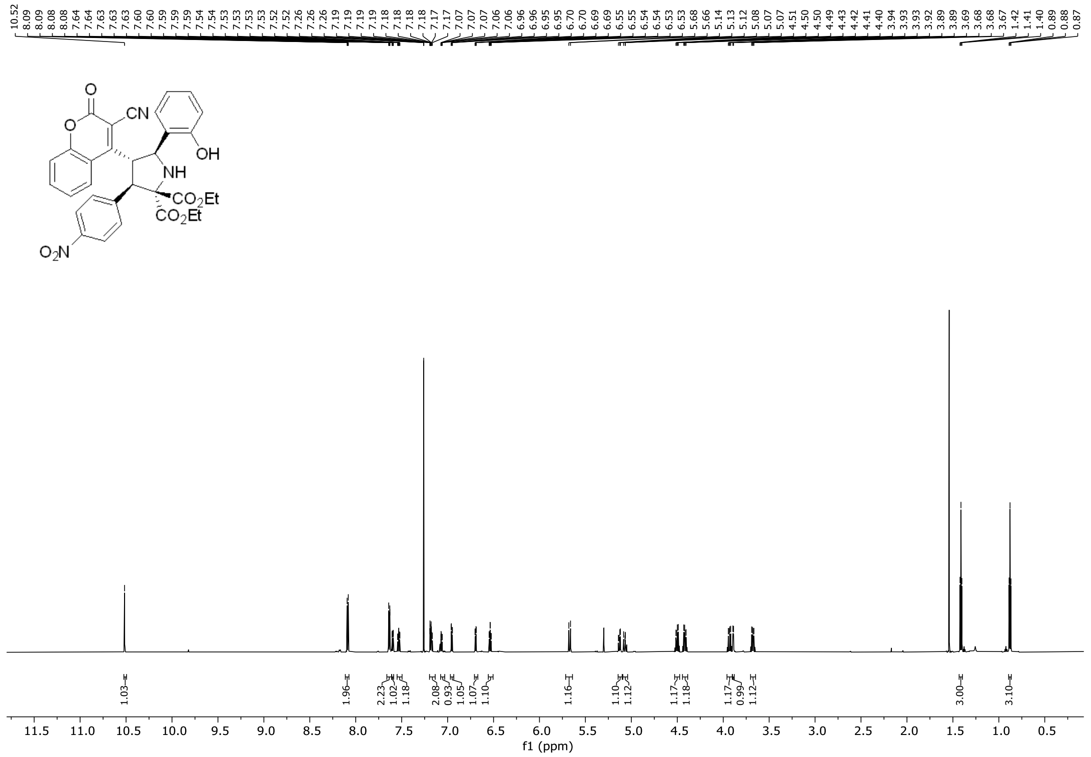

^{13}C NMR (176 MHz, $CDCl_3$)

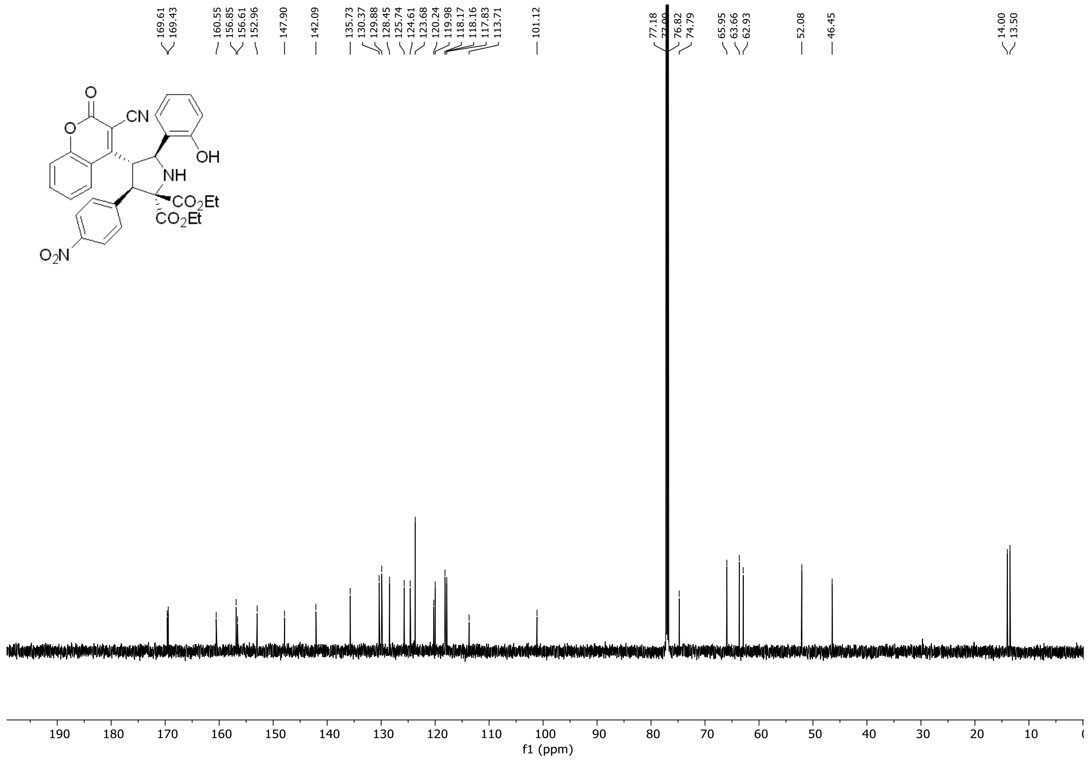

(3*R*,4*R*,5*S*)-Diethyl 4-(3-cyano-2-oxo-2*H*-chromen-4-yl)-5-(2-hydroxyphenyl)-3-(*p*-tolyl)-pyrrolidine-2,2-dicarboxylate **3g**

^{1}H NMR (700 MHz, $CDCl_3$)

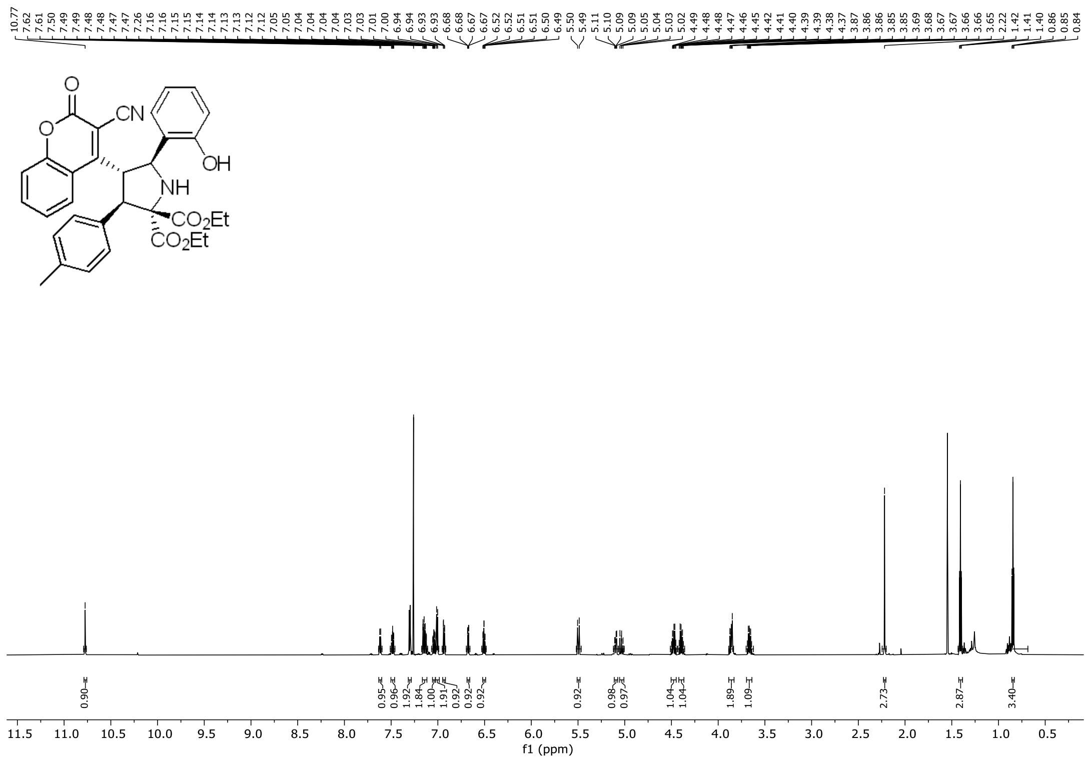

^{13}C NMR (176 MHz, $CDCl_3$)

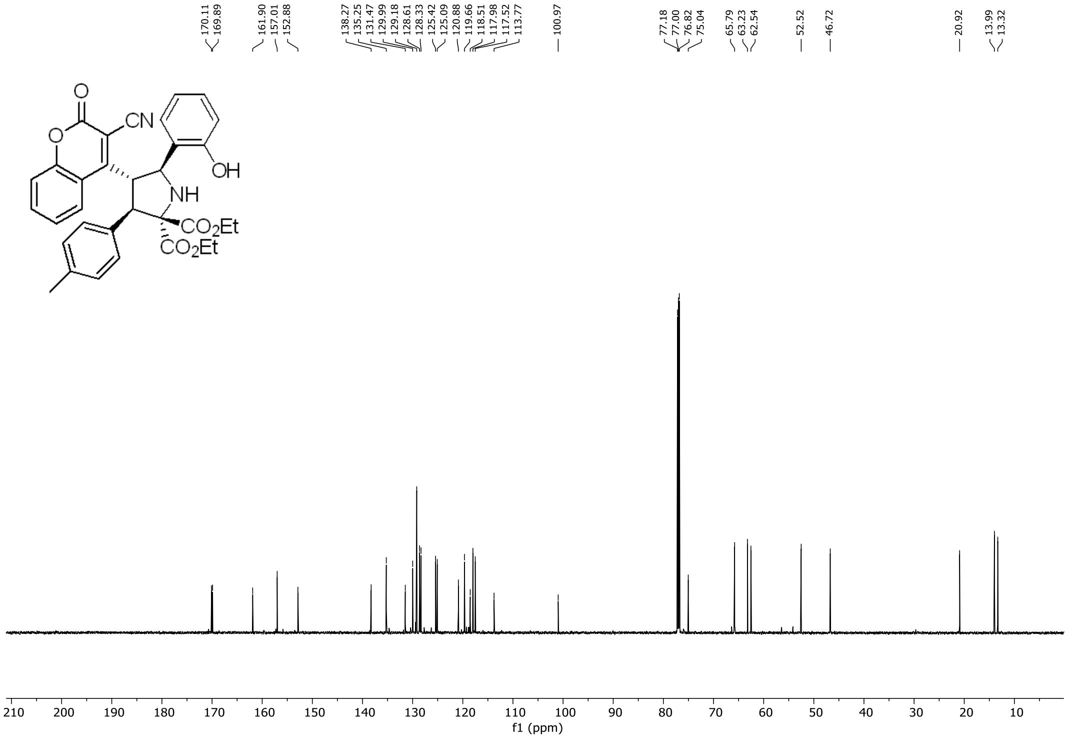

(3*R*,4*R*,5*S*)-Diethyl 4-(3-cyano-2-oxo-2*H*-chromen-4-yl)-5-(2-hydroxyphenyl)-3-(*m*-tolyl)-pyrrolidine-2,2-dicarboxylate **3h**

^{1}H NMR (700 MHz, $CDCl_3$)

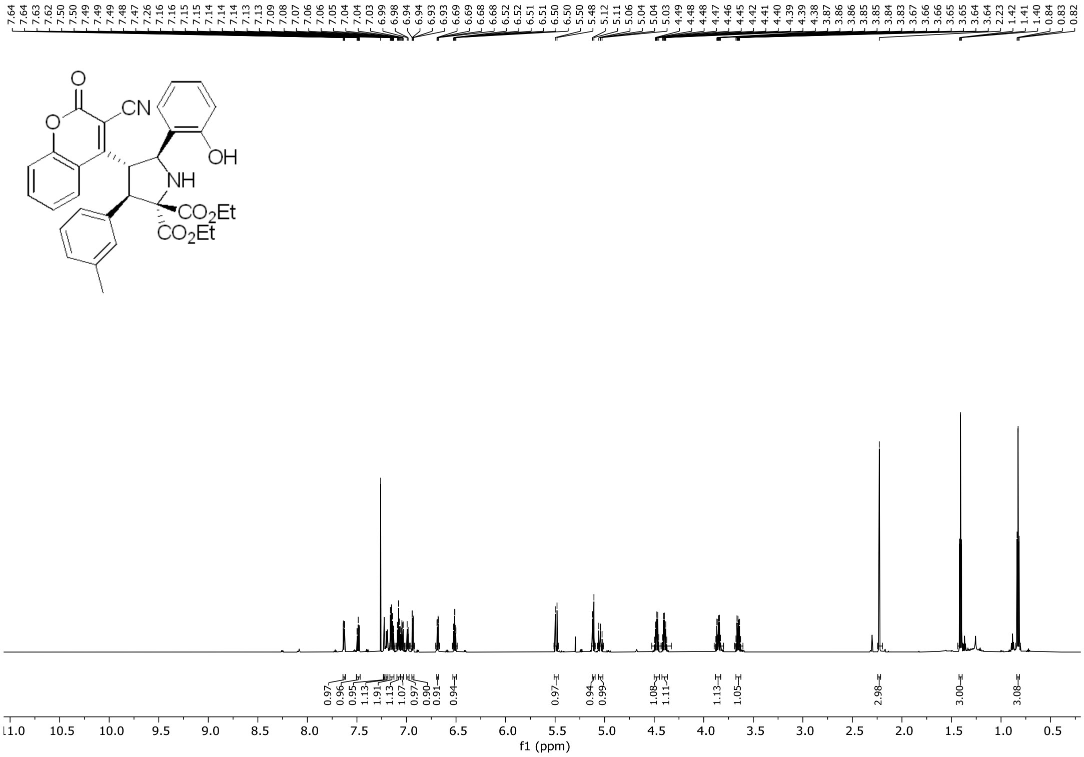

^{13}C NMR (176 MHz, $CDCl_3$)

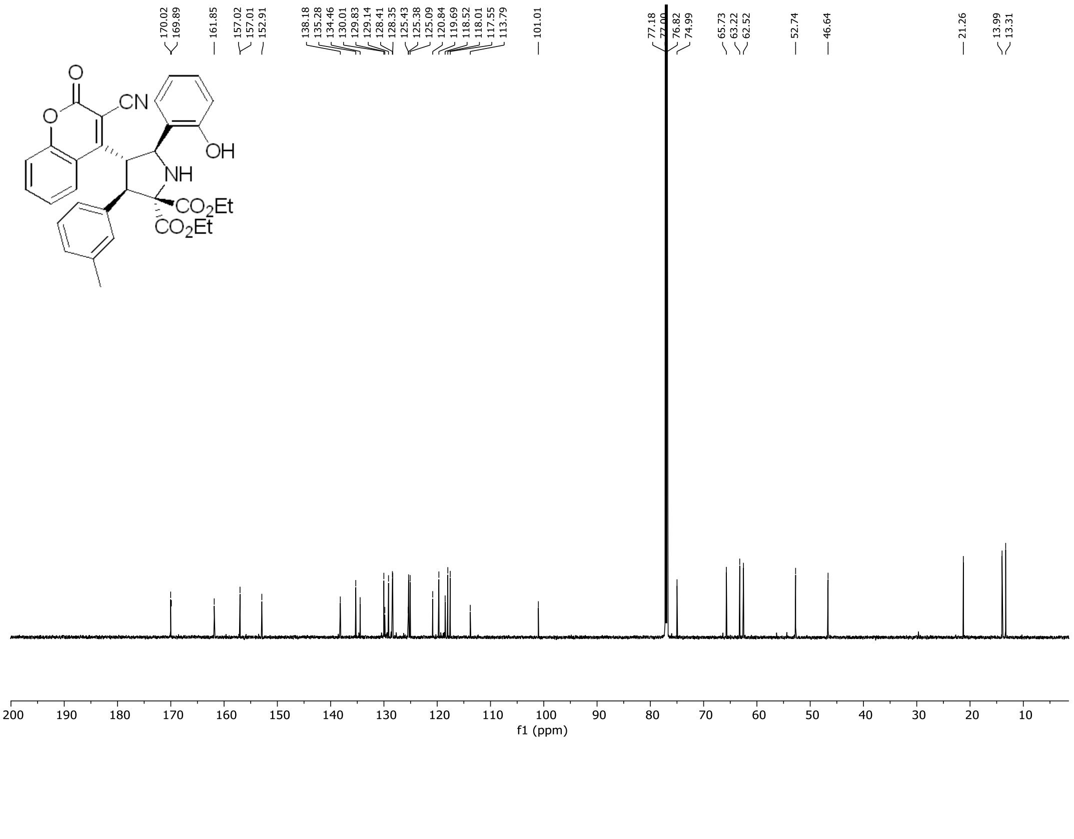

(3*R*,4*R*,5*S*)-Diethyl 4-(3-cyano-7-methoxy-2-oxo-2*H*-chromen-4-yl)-5-(2-hydroxyphenyl)-3-phenylpyrrolidine-2,2-dicarboxylate **3i**

^{1}H NMR (700 MHz, $CDCl_3$)

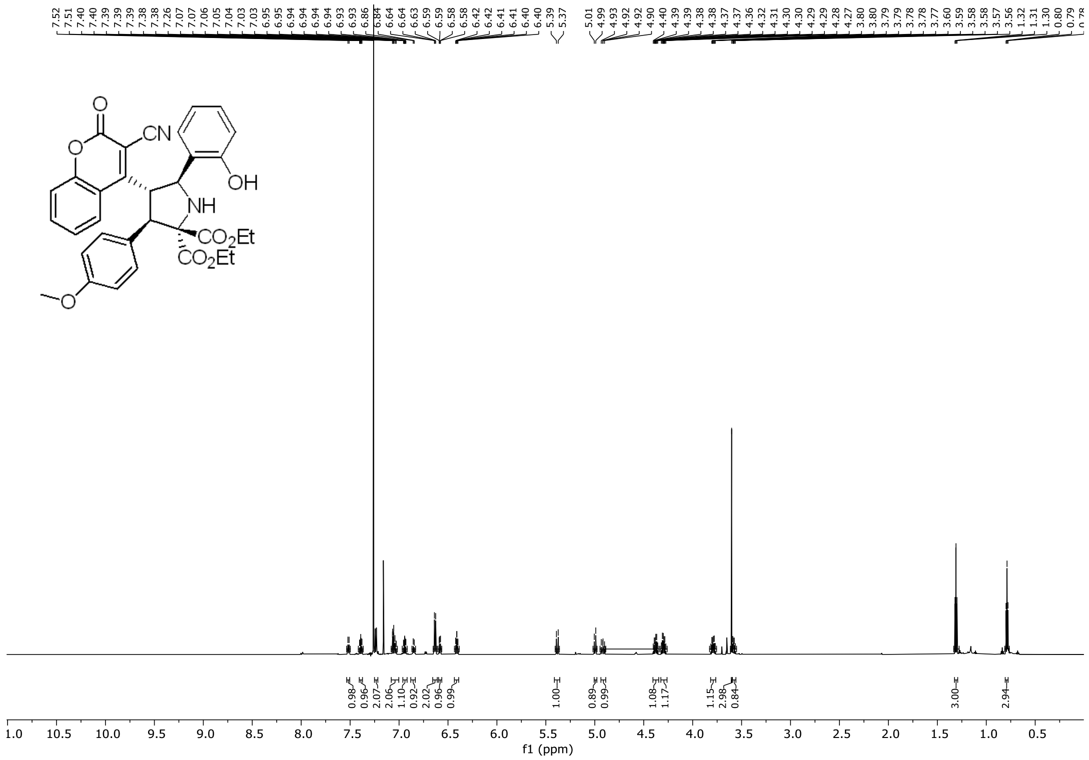

^{13}C NMR (176 MHz, $CDCl_3$)

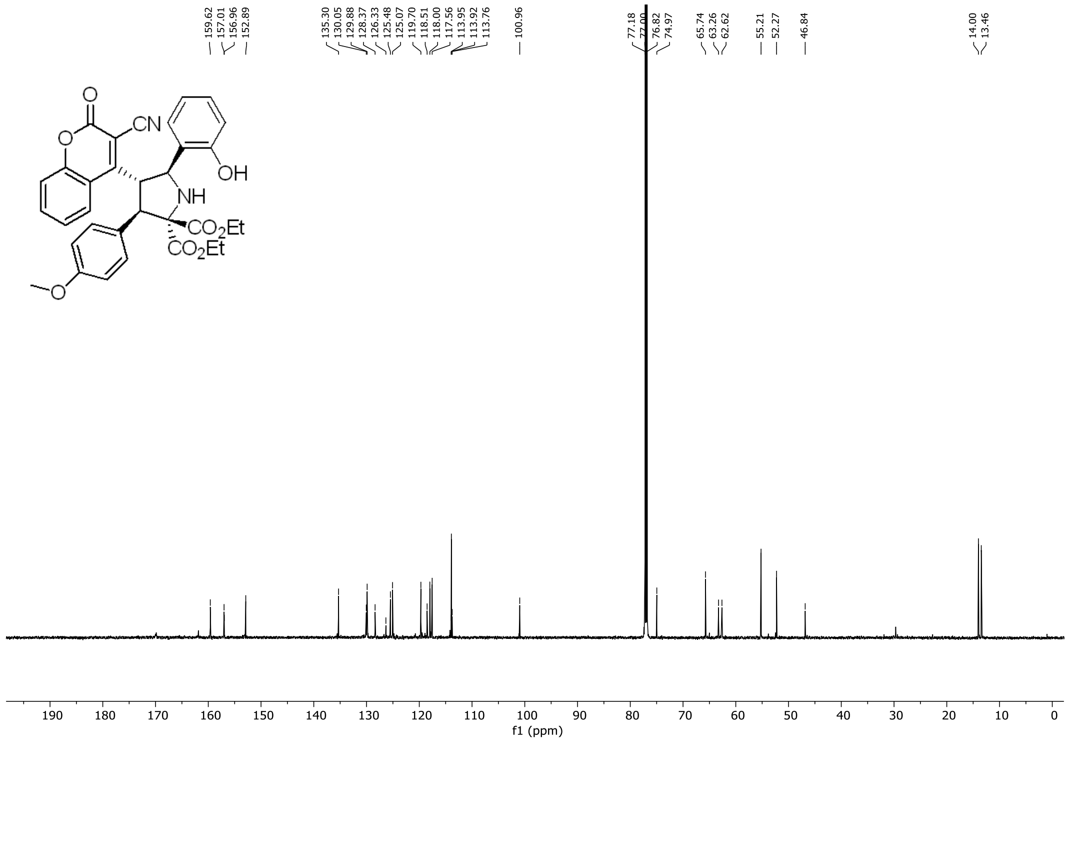

(3*R*,4*R*,5*S*)-Diethyl 4-(6-bromo-3-cyano-2-oxo-2*H*-chromen-4-yl)-5-(2-hydroxyphenyl)-3-phenylpyrrolidine-2,2-dicarboxylate **3j**

^{1}H NMR (700 MHz, $CDCl_3$)

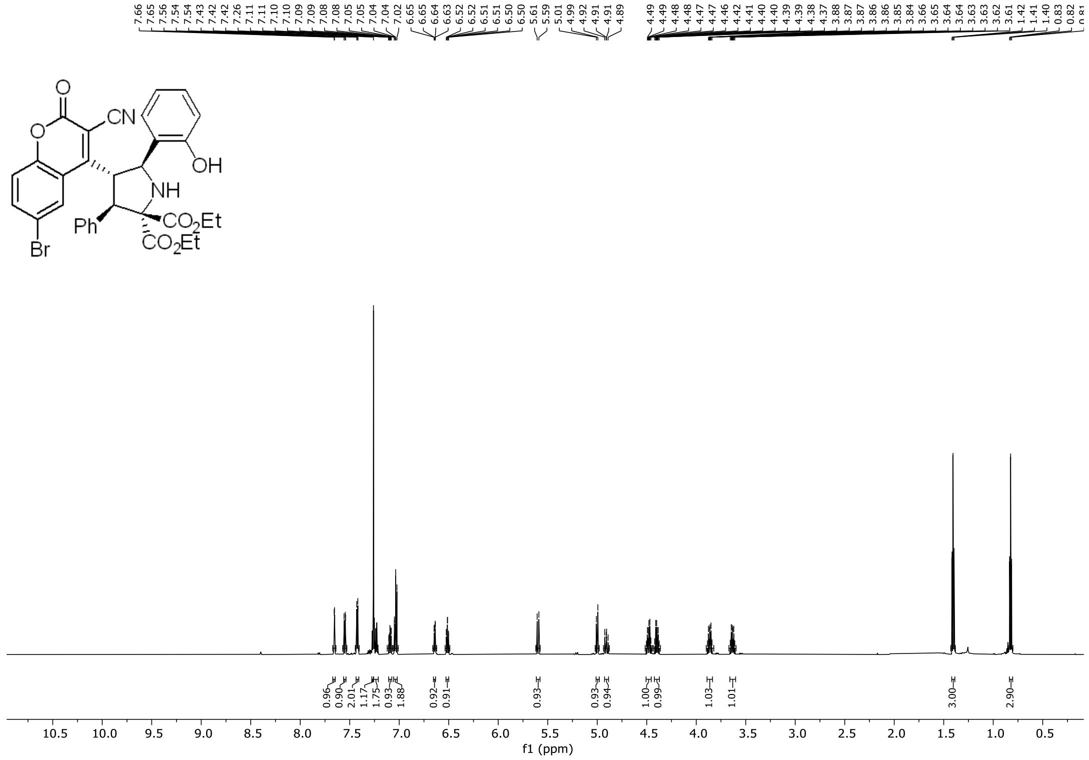

^{13}C NMR (176 MHz, $CDCl_3$)

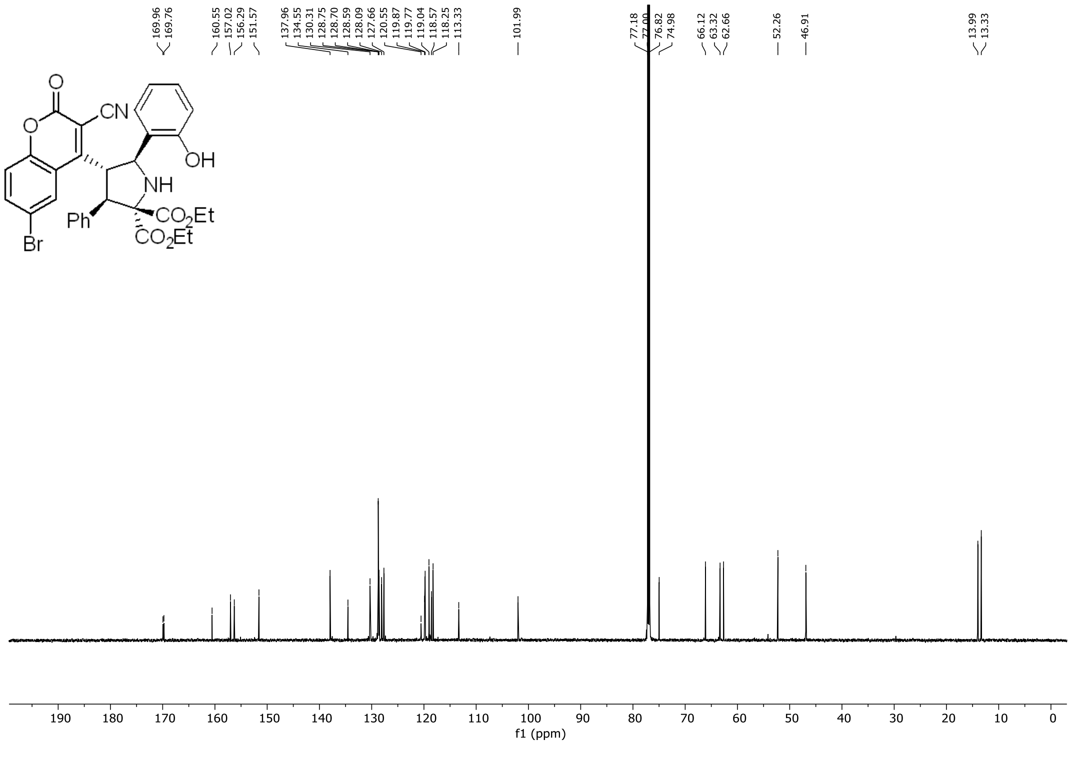

(3*R*,4*R*,5*S*)-Diethyl 4-(3-cyano-7-methoxy-2-oxo-2*H*-chromen-4-yl)-5-(2-hydroxyphenyl)-3-phenylpyrrolidine-2,2-dicarboxylate **3k**

^{1}H NMR (700 MHz, $CDCl_3$)

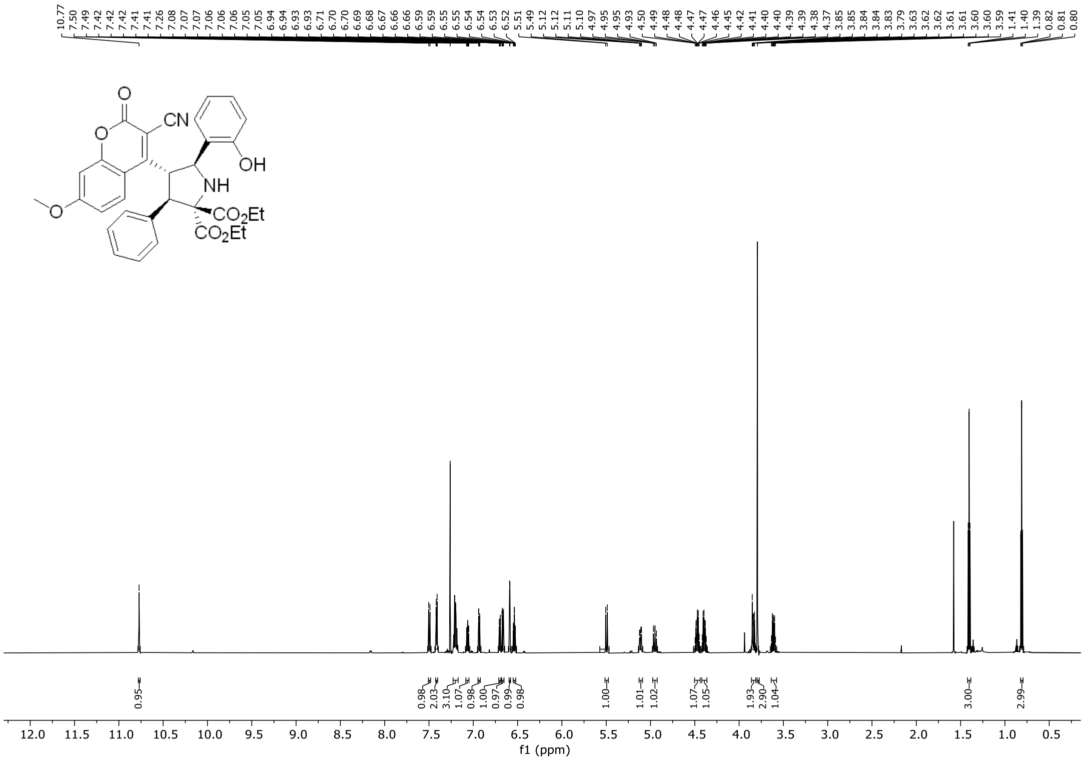

^{13}C NMR (176 MHz, $CDCl_3$)

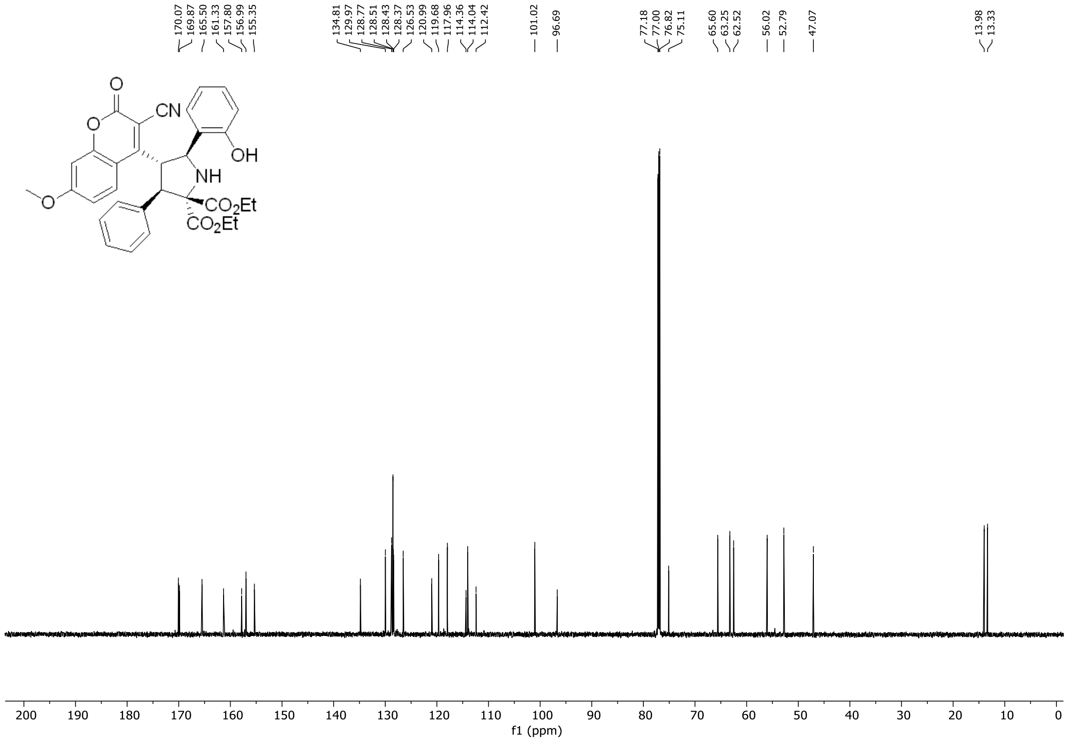

(3*R*,4*R*,5*S*)-Diethyl 5-(3-bromo-2-hydroxyphenyl)-4-(3-cyano-2-oxo-2*H*-chromen-4-yl)-3-phenylpyrrolidine-2,2-dicarboxylate **3m**

^{1}H NMR (700 MHz, $CDCl_3$)

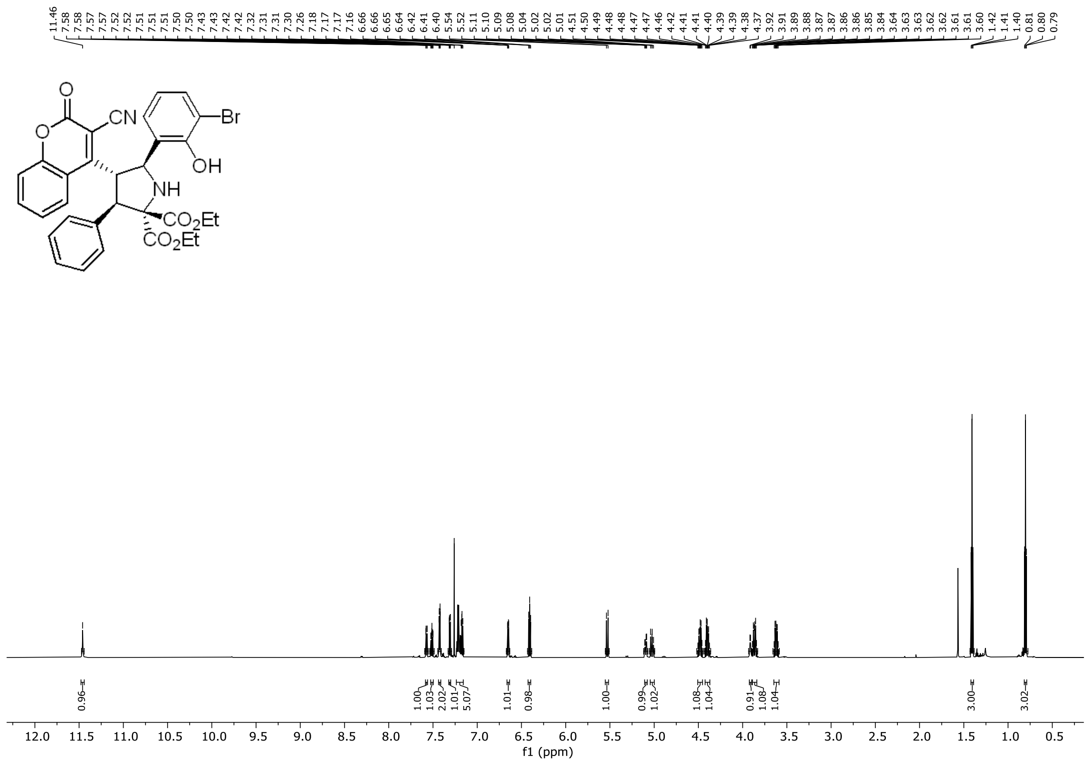

^{13}C NMR (176 MHz, $CDCl_3$)

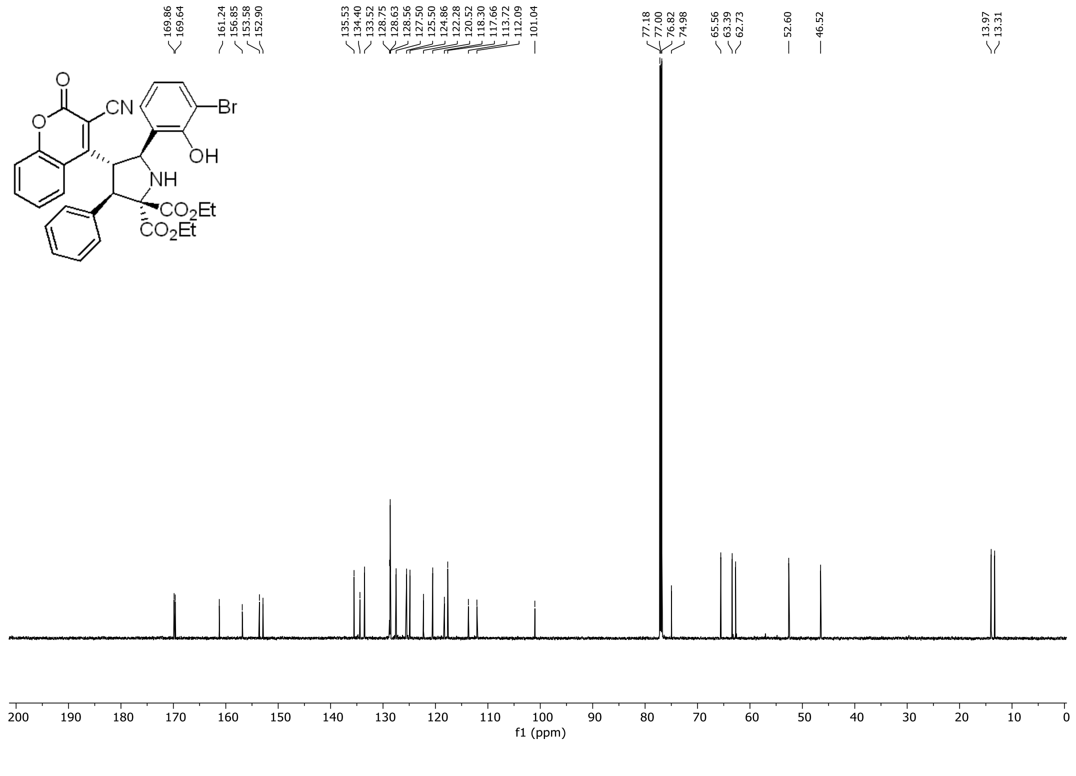

(3*R*,4*R*,5*S*)-Diethyl 5-(5-chloro-2-hydroxyphenyl)-4-(3-cyano-2-oxo-2*H*-chromen-4-yl)-3-phenylpyrrolidine-2,2-dicarboxylate **3n**

^{1}H NMR (700 MHz, $CDCl_3$)

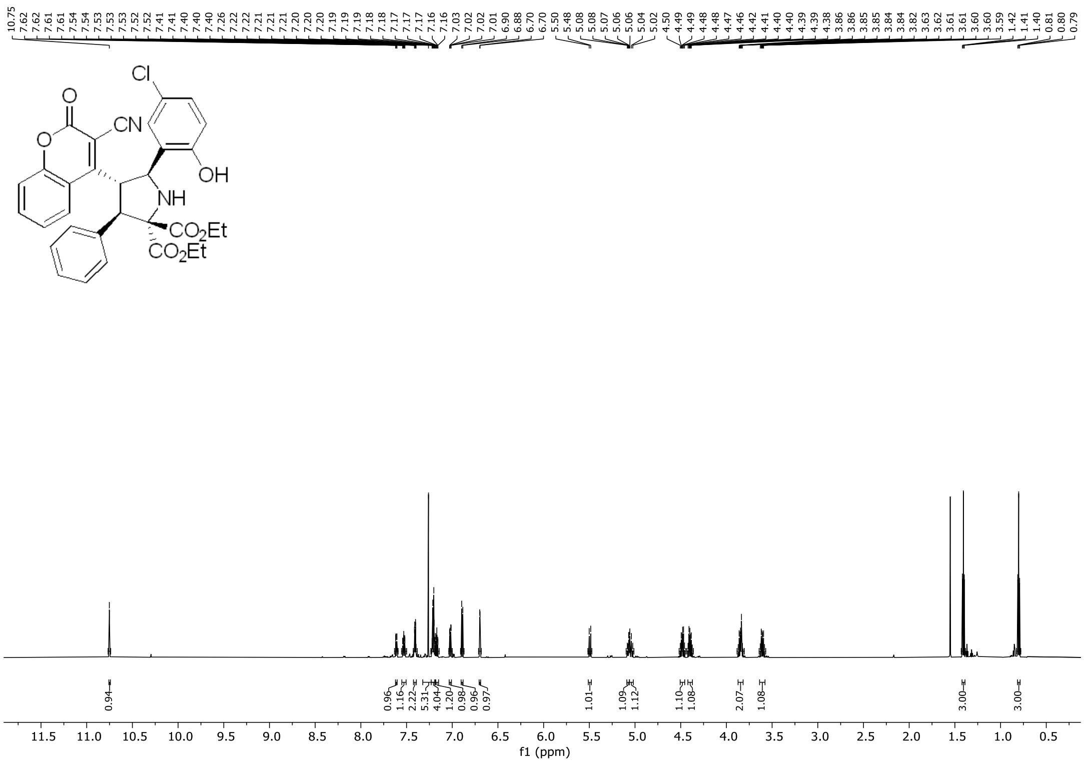

^{13}C NMR (176 MHz, $CDCl_3$)

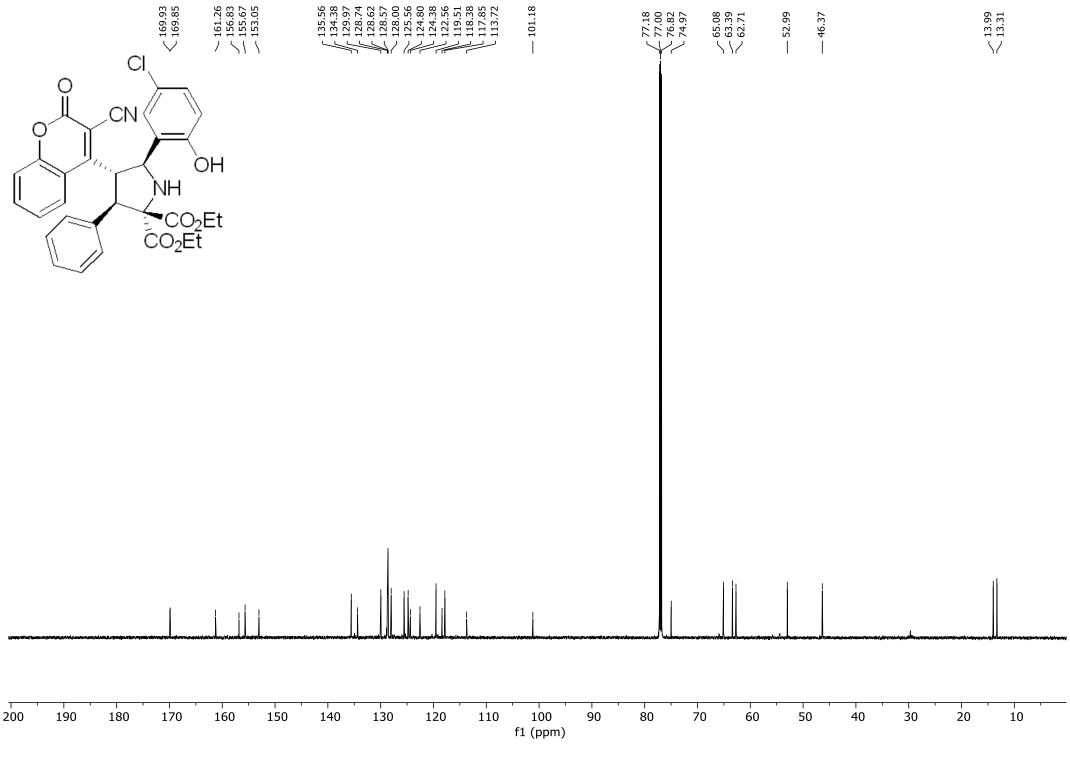

(3*R*,4*R*,5*S*)-Diethyl 4-(3-cyano-2-oxo-2*H*-chromen-4-yl)-5-(2-hydroxy-5-nitrophenyl)-3-phenylpyrrolidine-2,2-dicarboxylate **3o**

^{1}H NMR (700 MHz, $CDCl_3$)

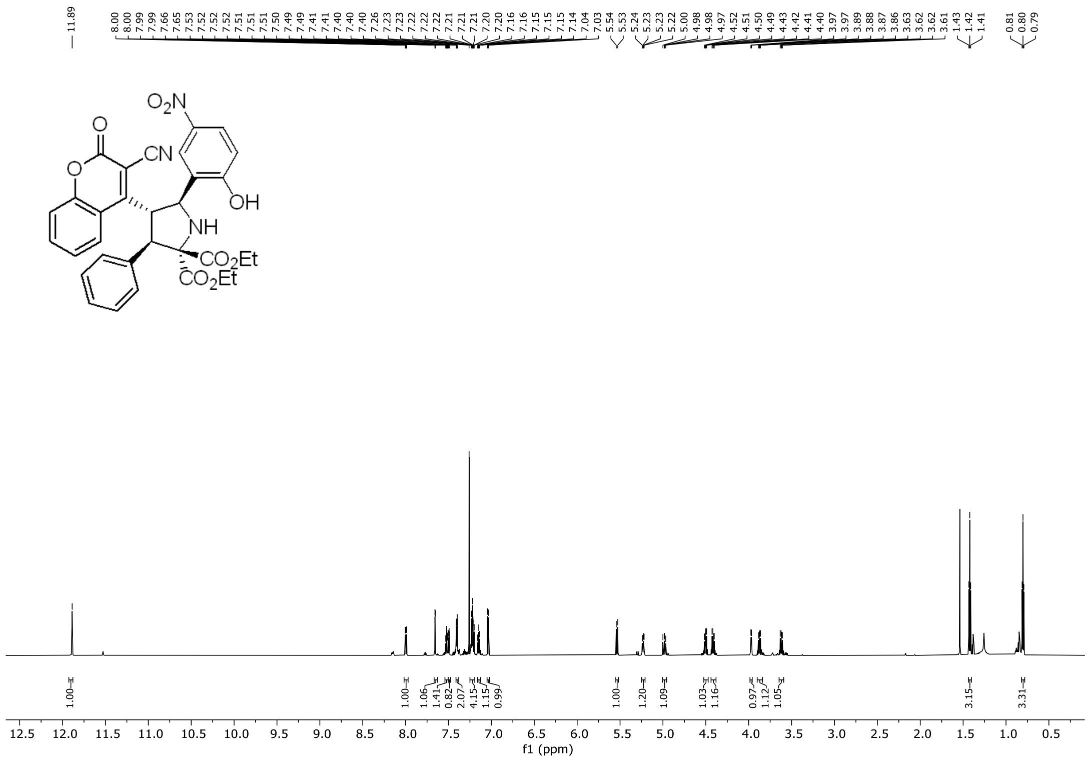

^{13}C NMR (176 MHz, $CDCl_3$)

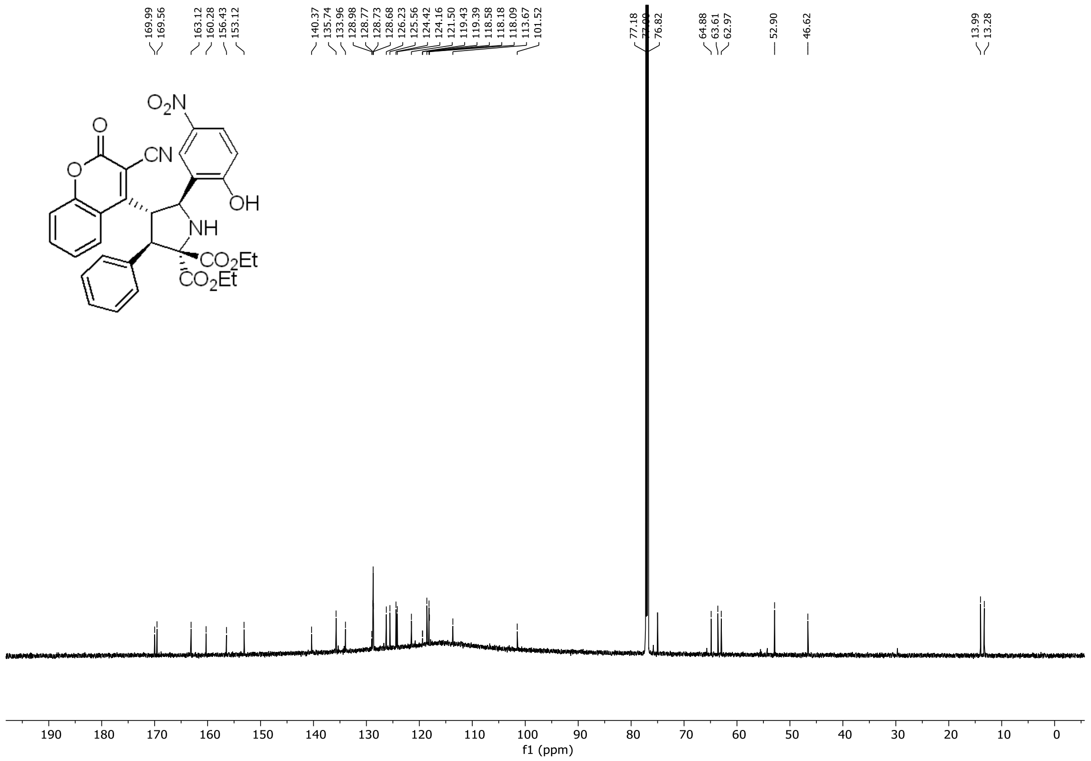

(3*R*,4*R*,5*S*)-Diethyl 4-(3-cyano-2-oxo-2*H*-chromen-4-yl)-5-(2-hydroxy-5-methylphenyl)-3-phenylpyrrolidine-2,2-dicarboxylate **3p**

^{1}H NMR (400 MHz, $CDCl_3$)

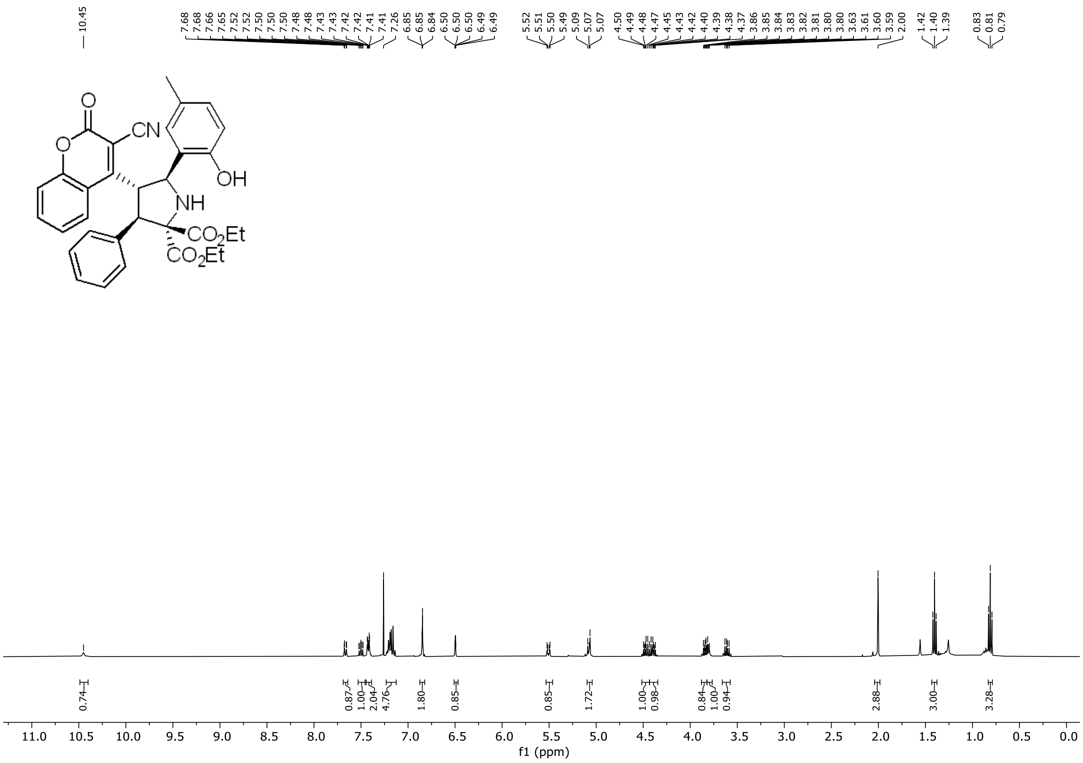

^{13}C NMR (101 MHz, $CDCl_3$)

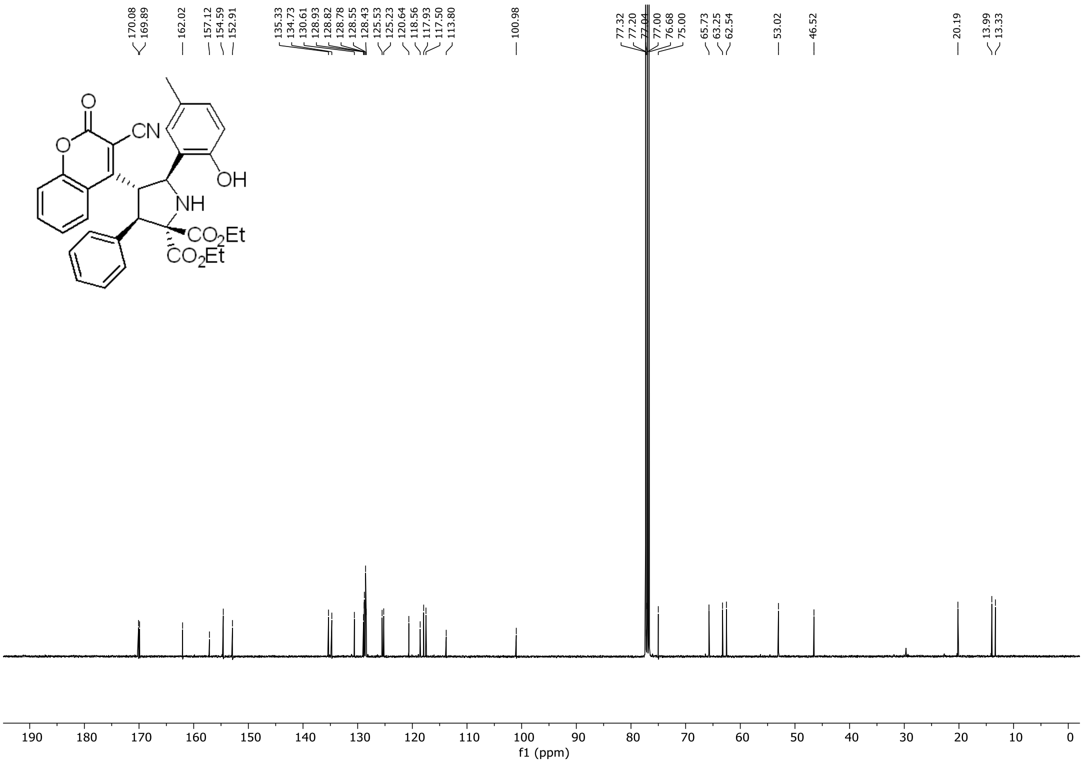

(3*R*,4*R*,5*S*)-Diethyl 5-(5-(*tert*-butyl)-2-hydroxyphenyl)-4-(3-cyano-2-oxo-2*H*-chromen-4-yl)-3-phenylpyrrolidine-2,2-dicarboxylate **3r**

^{1}H NMR (700 MHz, $CDCl_3$)

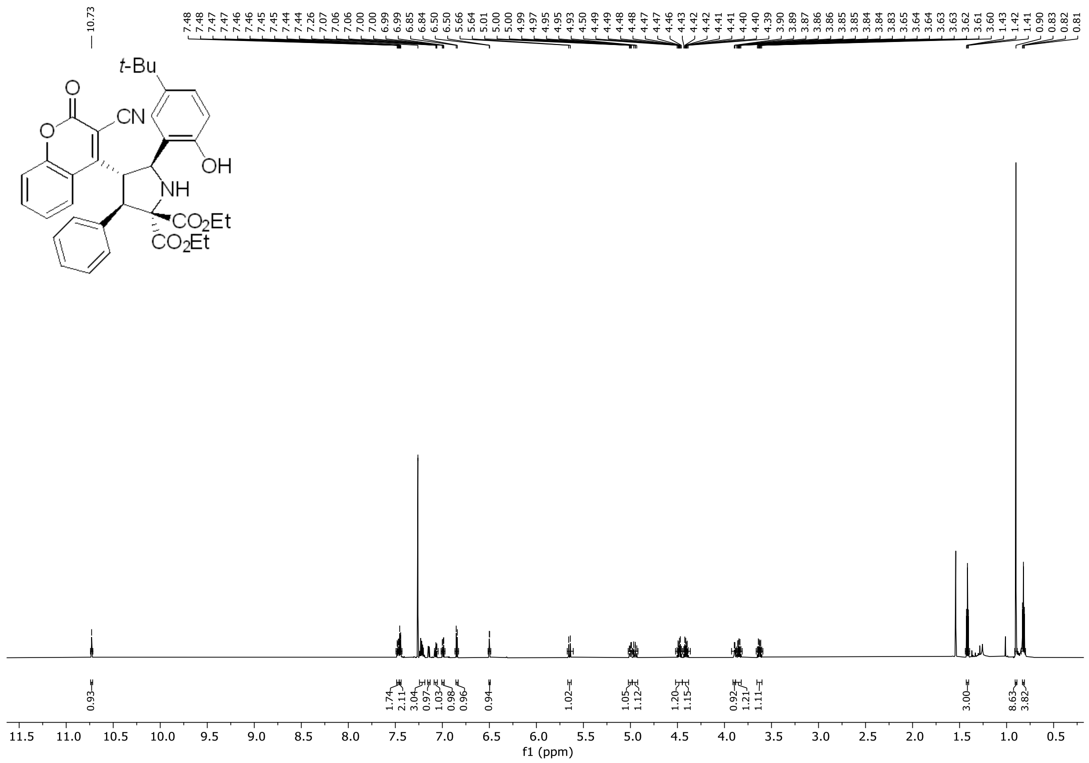

^{13}C NMR (176 MHz, $CDCl_3$)

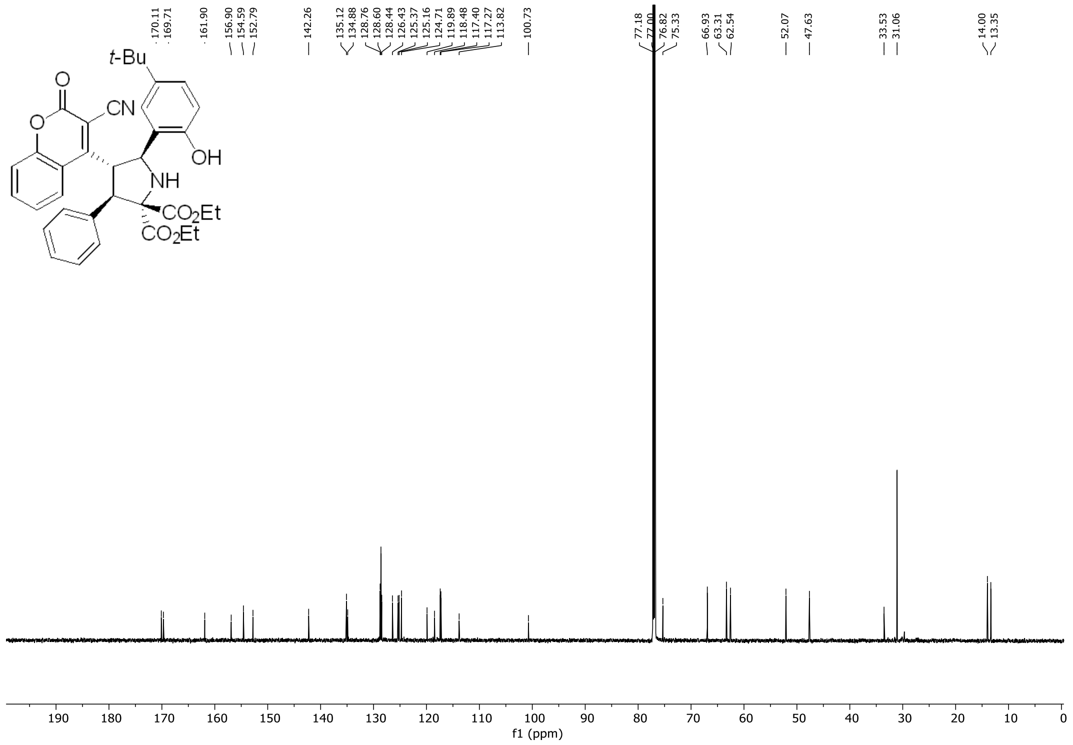

(3*R*,4*R*,5*S*)-Diethyl 4-(3-cyano-2-oxo-2*H*-chromen-4-yl)-5-(2-hydroxy-4,6-dimethoxy-phenyl)-3-phenylpyrrolidine-2,2-dicarboxylate **3s**

^{1}H NMR (400 MHz, CDCl$_3$)

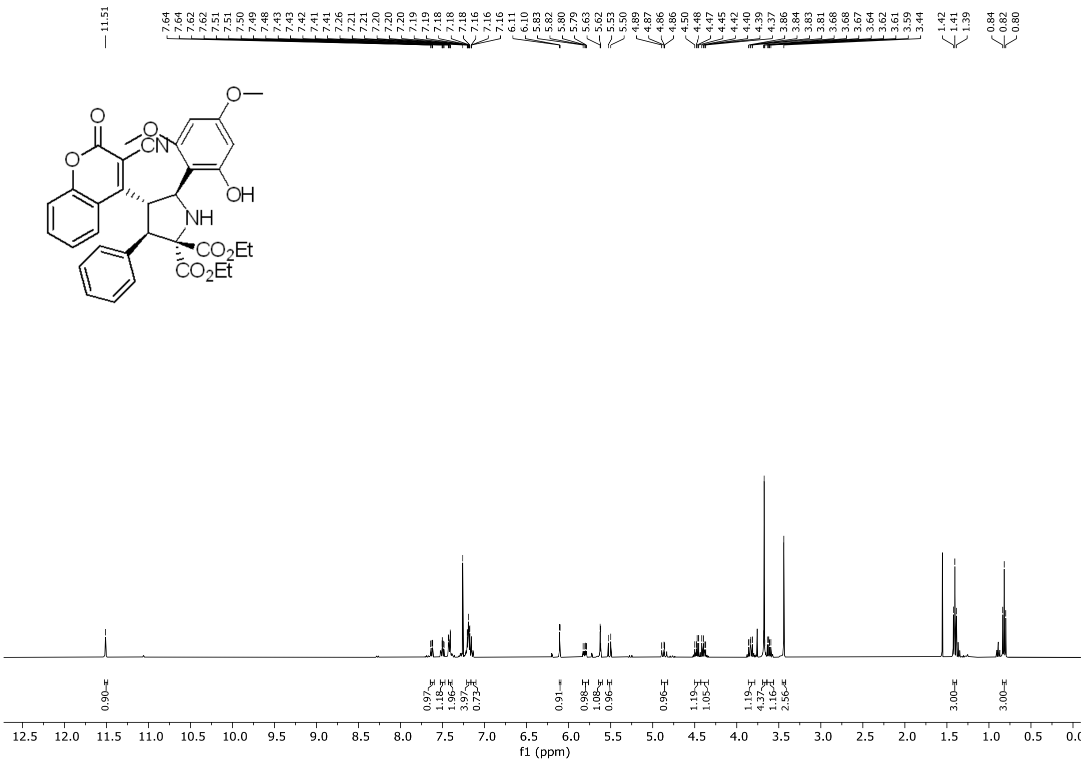

^{13}C NMR (101 MHz, CDCl$_3$)

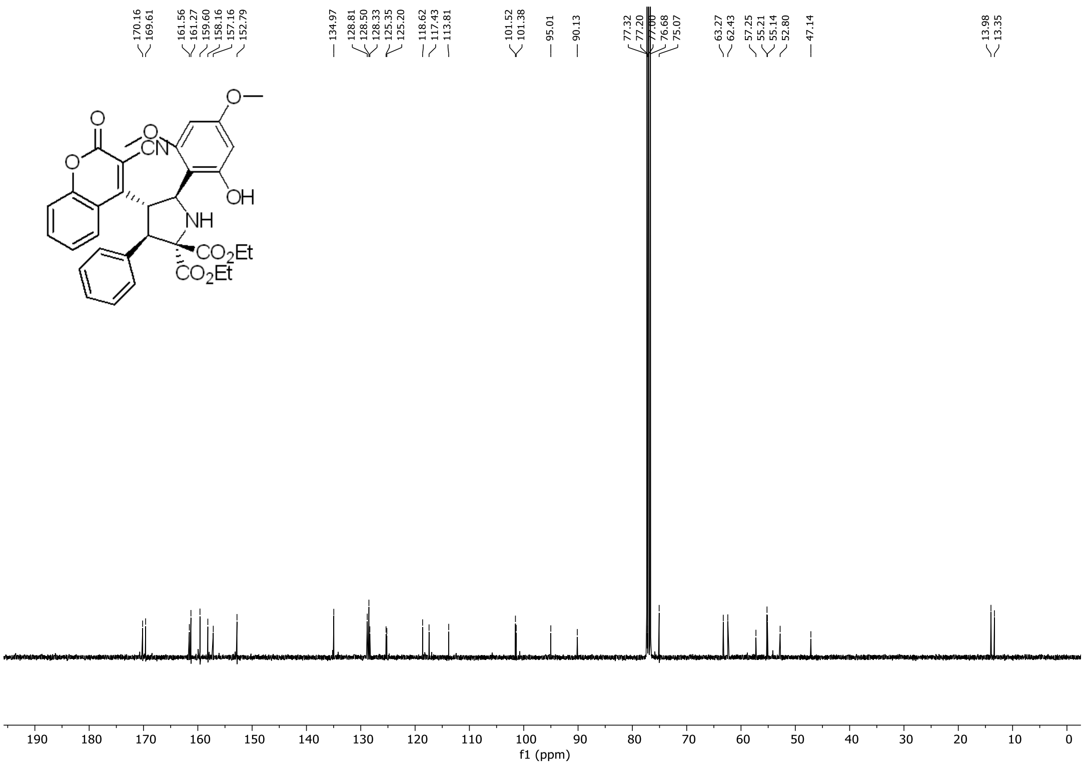

(1*R*,2*R*,10b*S*)-Diethyl 1-(3-cyano-2-oxo-2*H*-chromen-4-yl)-5-oxo-2-phenyl-5,10*b*-dihydro-1*H*-benzo-[*e*]pyrrolo[1,2-*c*][1,3]oxazine-3,3(2*H*)-dicarboxylate **5a**

^{1}H NMR (700 MHz, $CDCl_3$)

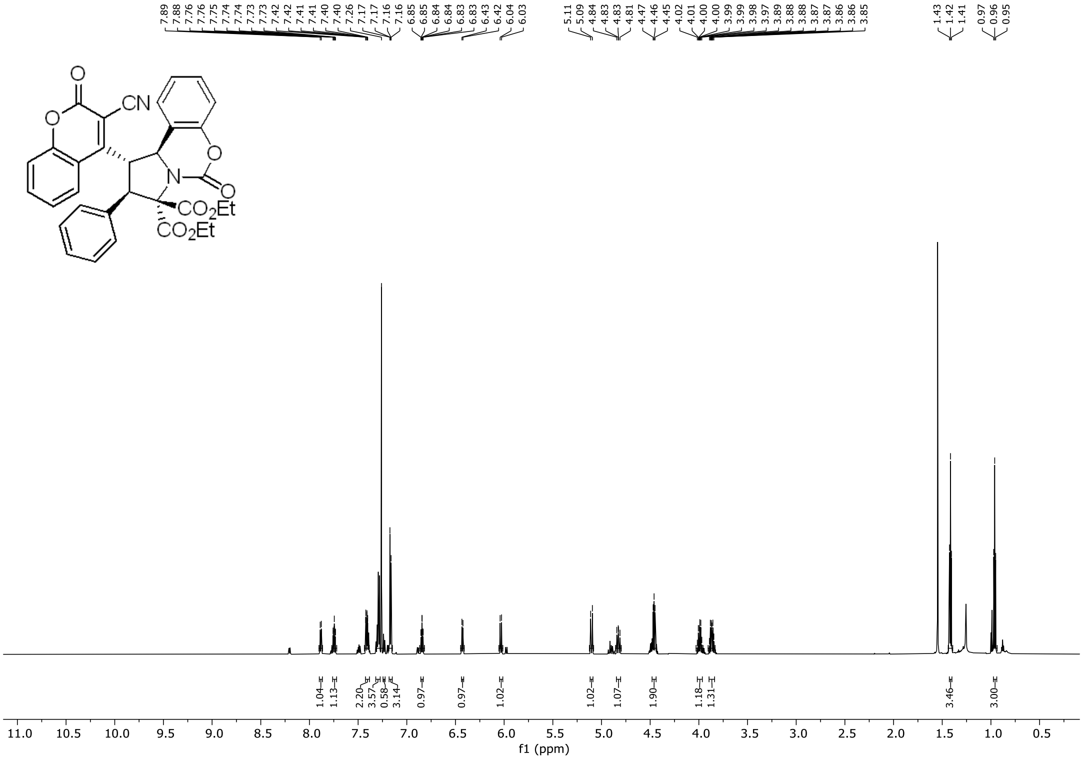

^{13}C NMR (176 MHz, $CDCl_3$)

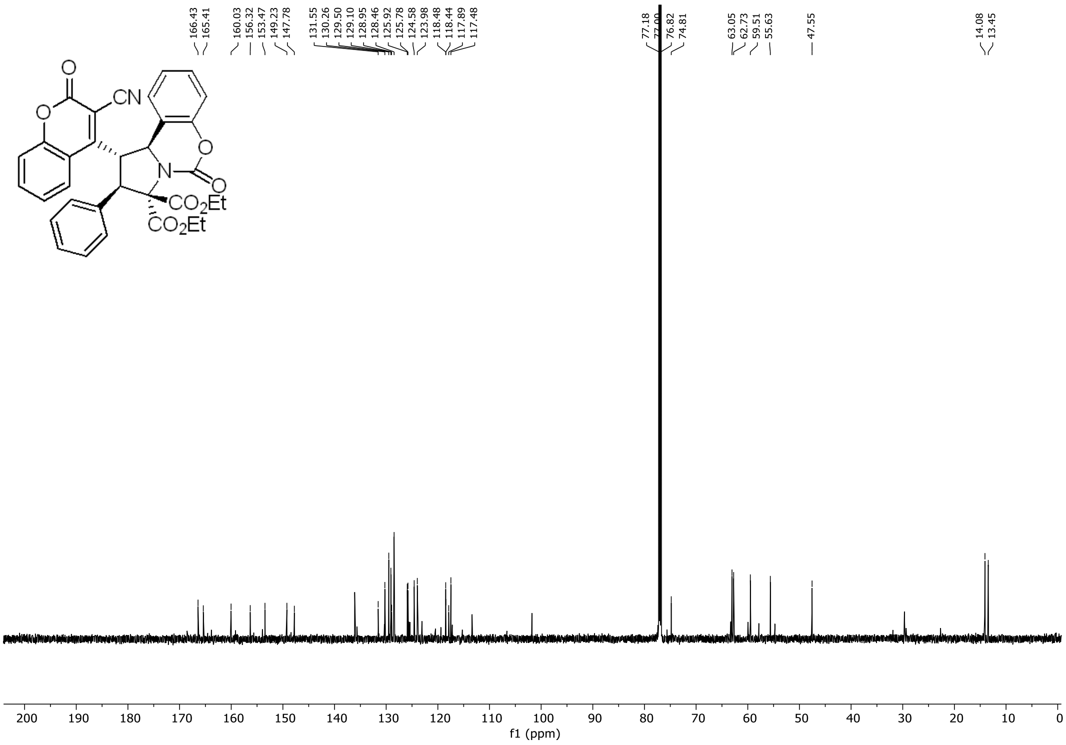

(1*R*,2*R*,10b*S*)-Diethyl 2-(4-chlorophenyl)-1-(3-cyano-2-oxo-2*H*-chromen-4-yl)-5-oxo-5,10*b*-dihydro-1*H*-benzo[e]-pyrrolo[1,2-*c*][1,3]oxazine-3,3(2*H*)-dicarboxylate **5b**

^{1}H NMR (700 MHz, $CDCl_3$)

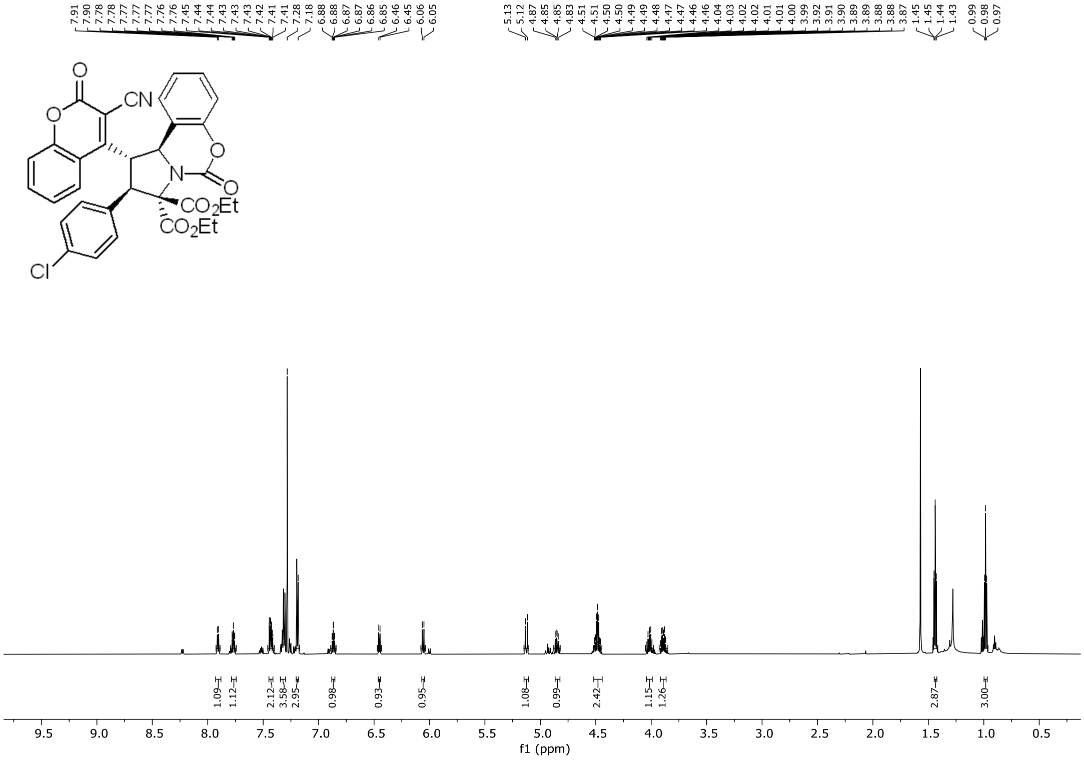

^{13}C NMR (176 MHz, $CDCl_3$)

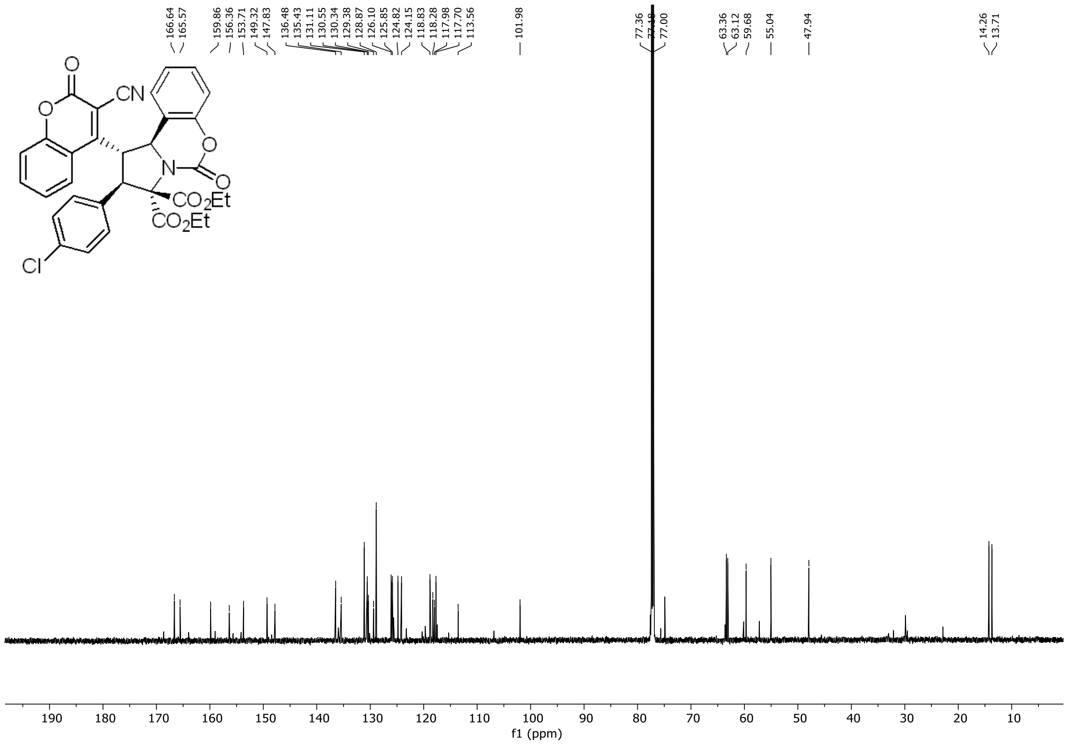

(1*R*,2*R*,10b*S*)-Diethyl 7-bromo-1-(3-cyano-2-oxo-2*H*-chromen-4-yl)-5-oxo-2-phenyl-5,10*b*-dihydro-1*H*-benzo[*e*]py-rrolo[1,2-*c*][1,3]oxazine-3,3(2*H*)-dicarboxylate **5c**

^{1}H NMR (700 MHz, $CDCl_3$)

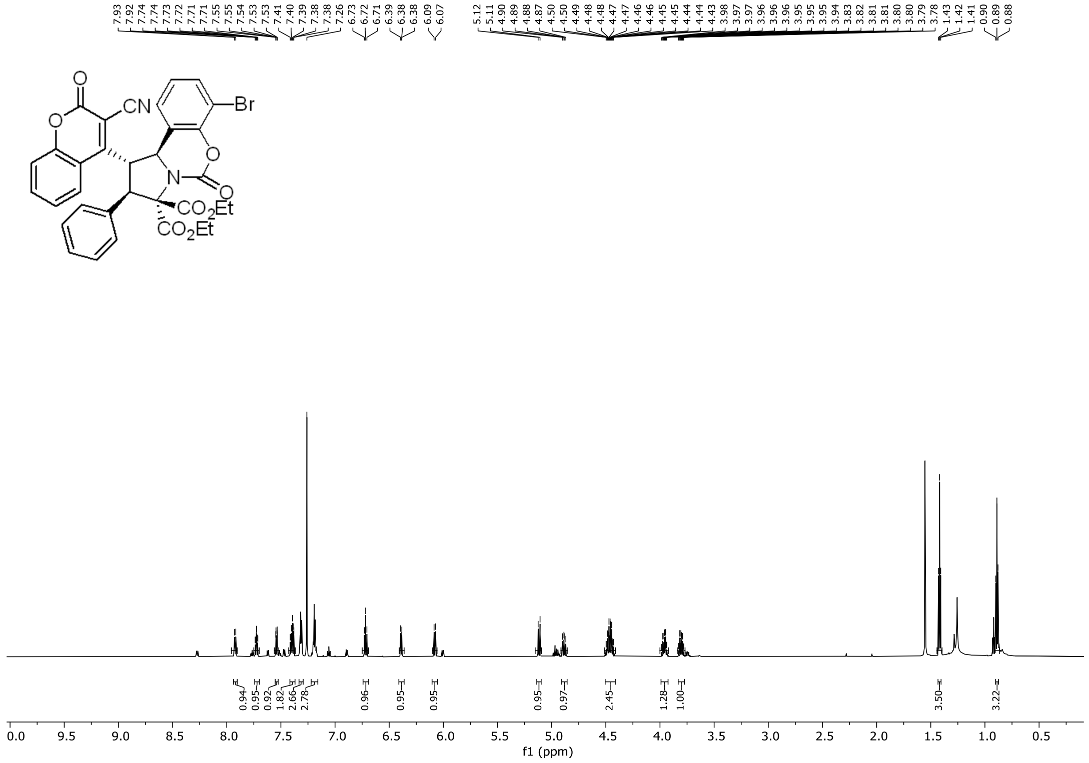

^{13}C NMR (176 MHz, $CDCl_3$)

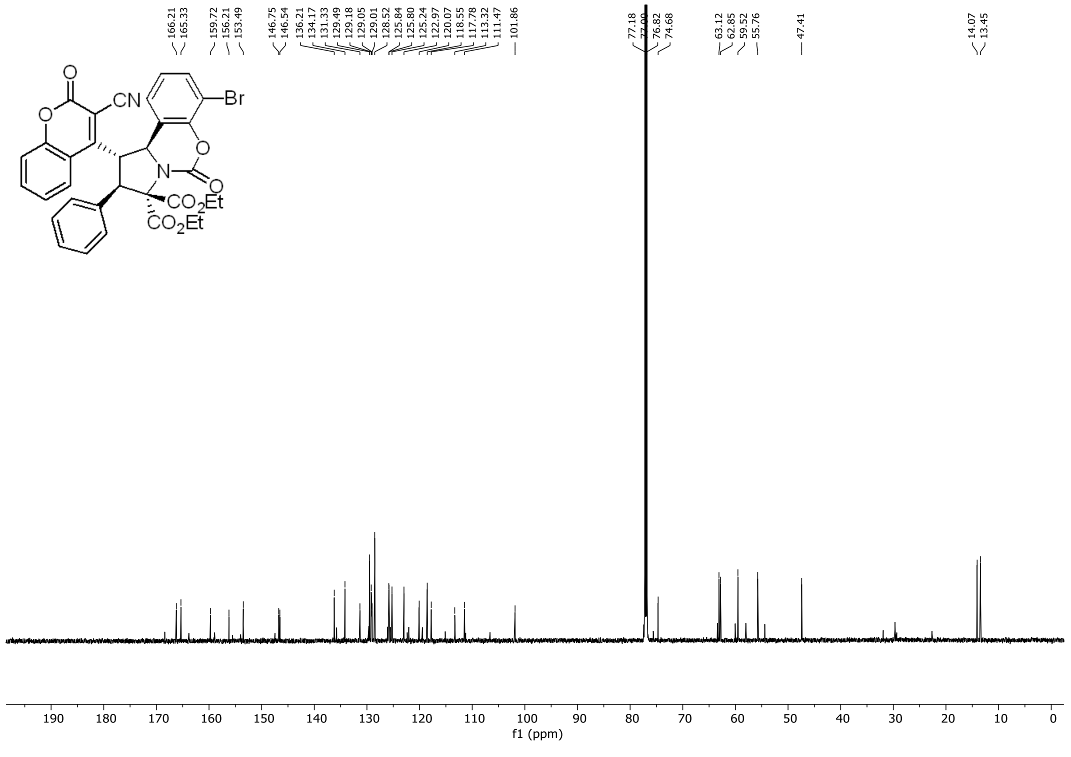

6. UPC^2 traces

(3*R*,4*R*,5*S*)-Diethyl 4-(3-cyano-2-oxo-2*H*-chromen-4-yl)-5-(2-hydroxyphenyl)-3-phenyl-pyrrolidine-2,2-dicarboxylate **3a**

Racemic sample

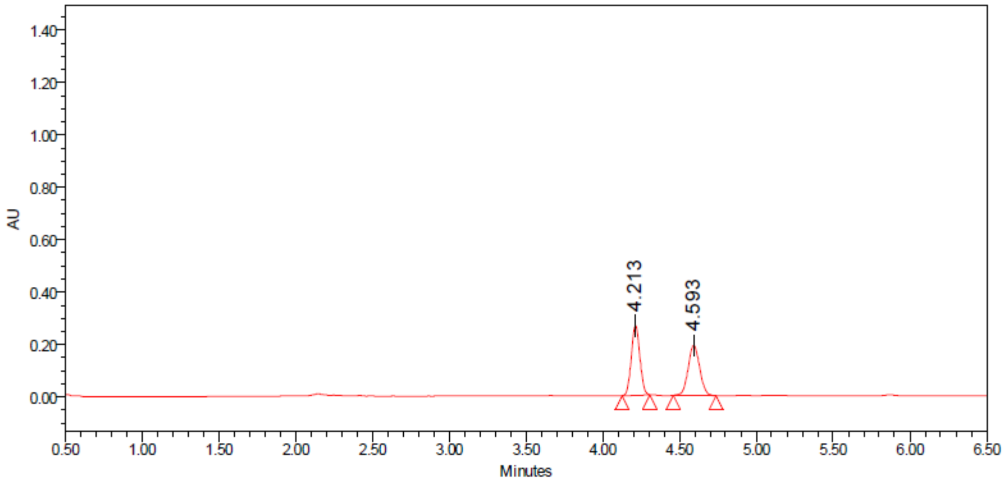

Peak Results

	RT	% Area
1	4.213	50.94
2	4.593	49.06

Enantiomerically enriched sample

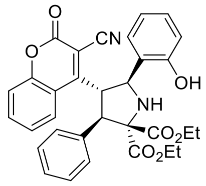

3a

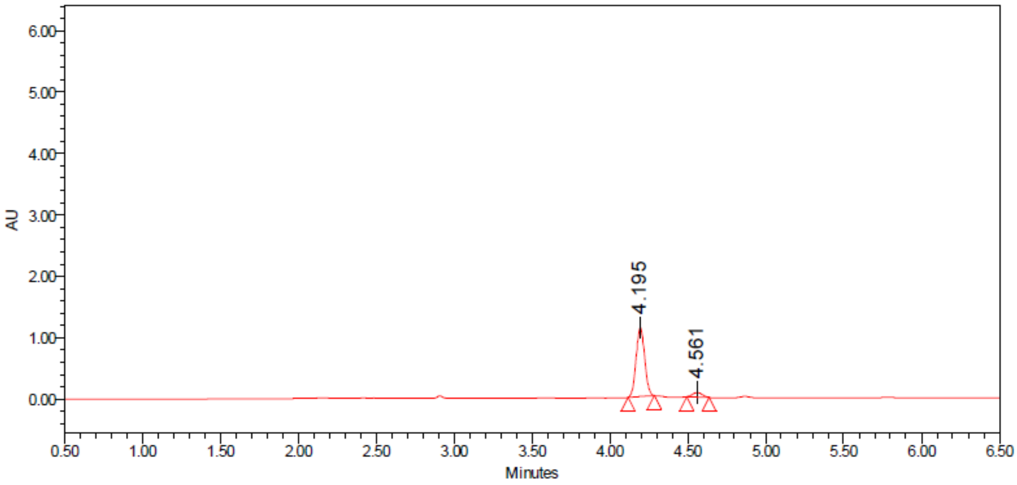

Peak Results

	RT	% Area
1	4.195	93.03
2	4.561	6.97

(3*R*,4*R*,5*S*)-Diethyl 3-(4-chlorophenyl)-4-(3-cyano-2-oxo-2*H*-chromen-4-yl)-5-(2-hydroxy-phenyl)pyrrolidine-2,2-dicarboxylate **3b**

Racemic sample

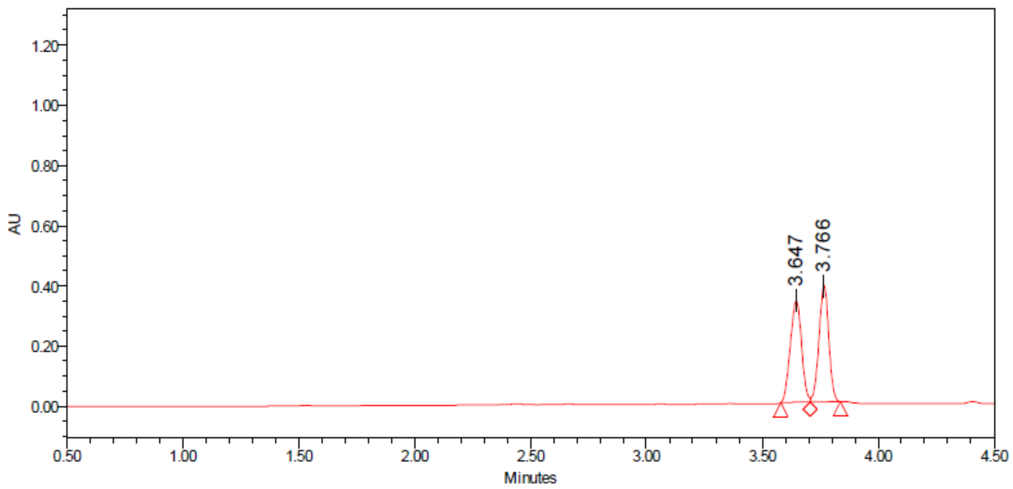

Peak Results

	RT	% Area
1	3.647	50.11
2	3.766	49.89

Enantiomerically enriched sample

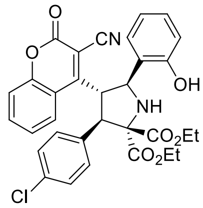

3b

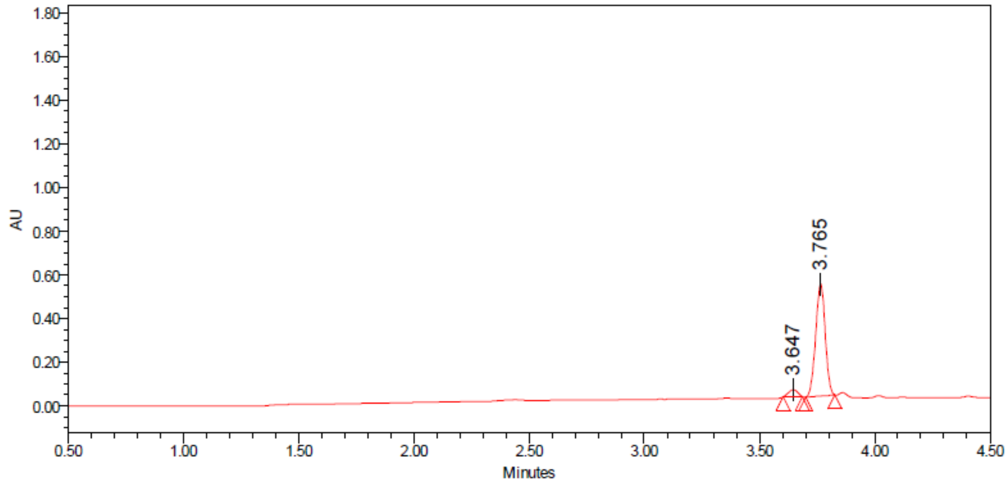

Peak Results

	RT	% Area
1	3.647	5.56
2	3.765	94.44

(3*R*,4*R*,5*S*)-Diethyl 3-(3-chlorophenyl)-4-(3-cyano-2-oxo-2*H*-chromen-4-yl)-5-(2-hydroxy-phenyl)pyrrolidine-2,2-dicarboxylate **3c**

Racemic sample

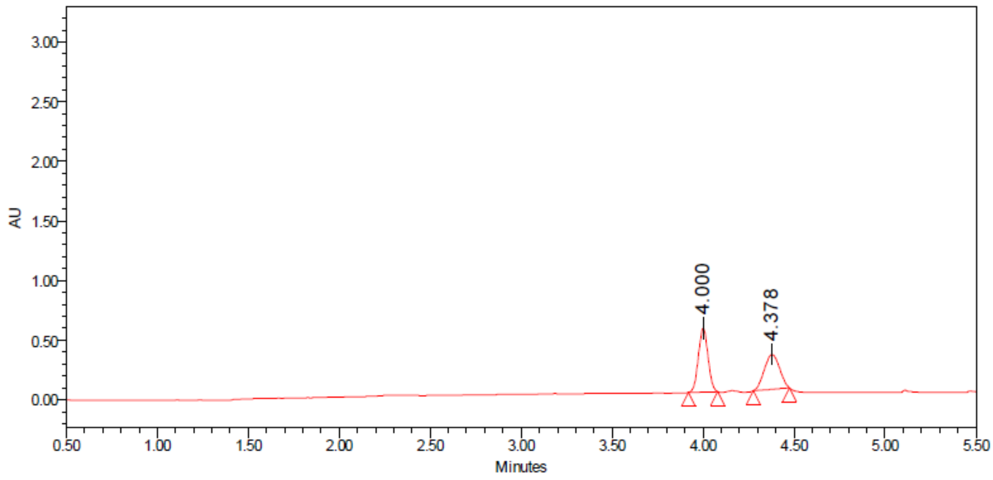

Peak Results

	RT	% Area
1	4.000	53.79
2	4.378	46.21

Enantiomerically enriched sample

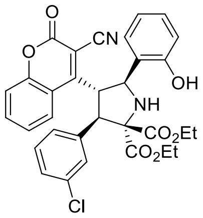

3c

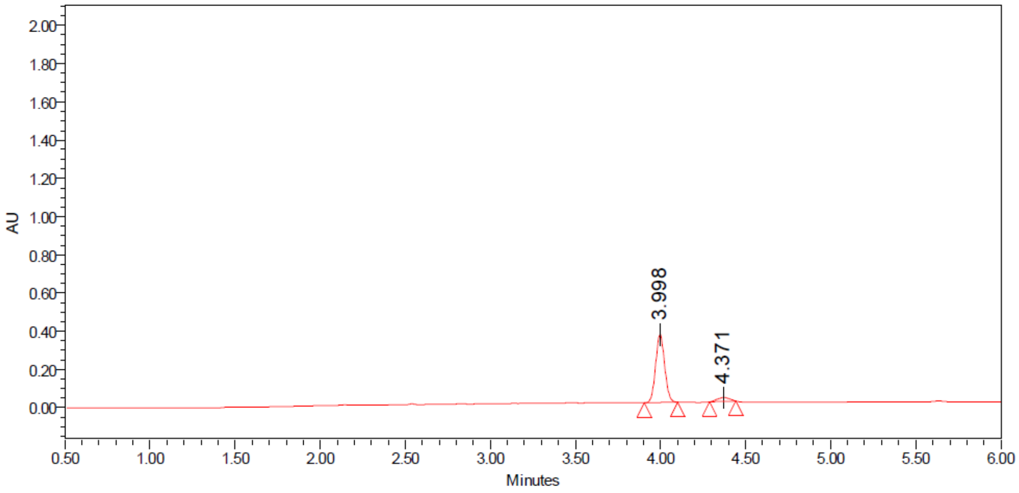

Peak Results

	RT	% Area
1	3.998	92.86
2	4.371	7.14

(3*R*,4*R*,5*S*)-Diethyl 4-(3-cyano-2-oxo-2*H*-chromen-4-yl)-3-(3-fluorophenyl)-5-(2-hydroxy-phenyl)pyrolidine-2,2-dicarboxylate **3d**

Racemic sample

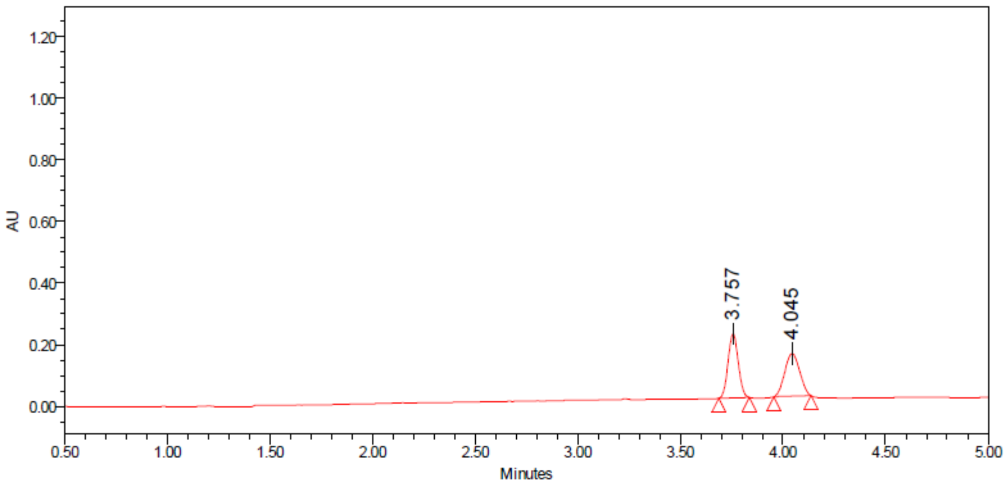

Peak Results

	RT	% Area
1	3.757	50.71
2	4.045	49.29

Enantiomerically enriched sample

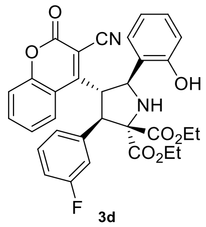

3d

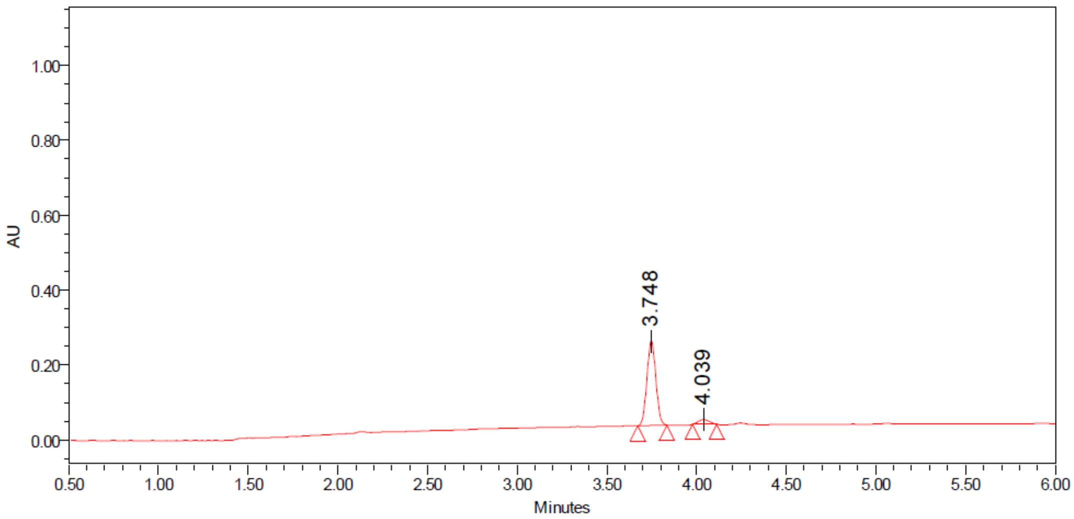

Peak Results

	RT	% Area
1	3.748	93.47
2	4.039	6.53

(3*R*,4*R*,5*S*)-Diethyl 4-(3-cyano-2-oxo-2*H*-chromen-4-yl)-5-(2-hydroxyphenyl)-3-(4-(trifluoro-methyl)phenyl)pyrolidine-2,2-dicarboxylate **3e**

Racemic sample

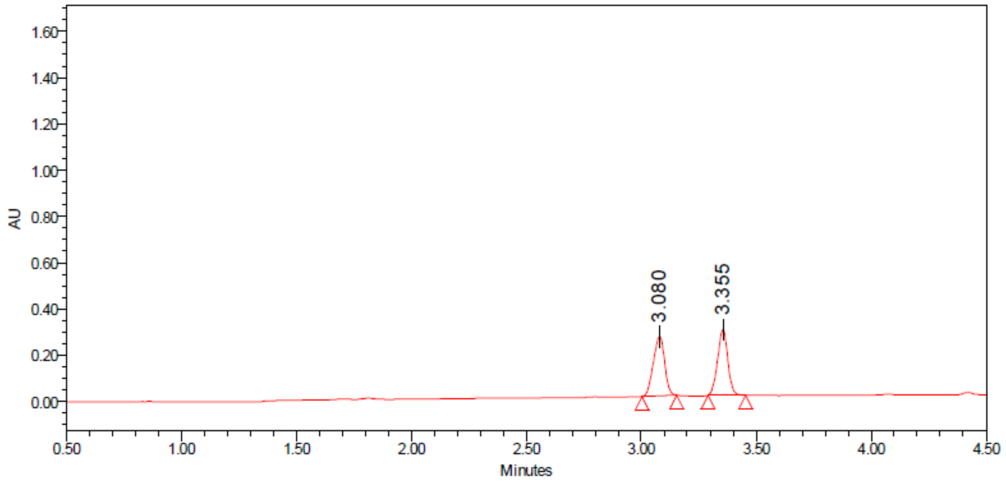

Peak Results

	RT	% Area
1	3.080	49.99
2	3.355	50.01

Enantiomerically enriched sample

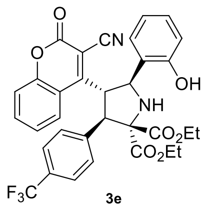

3e

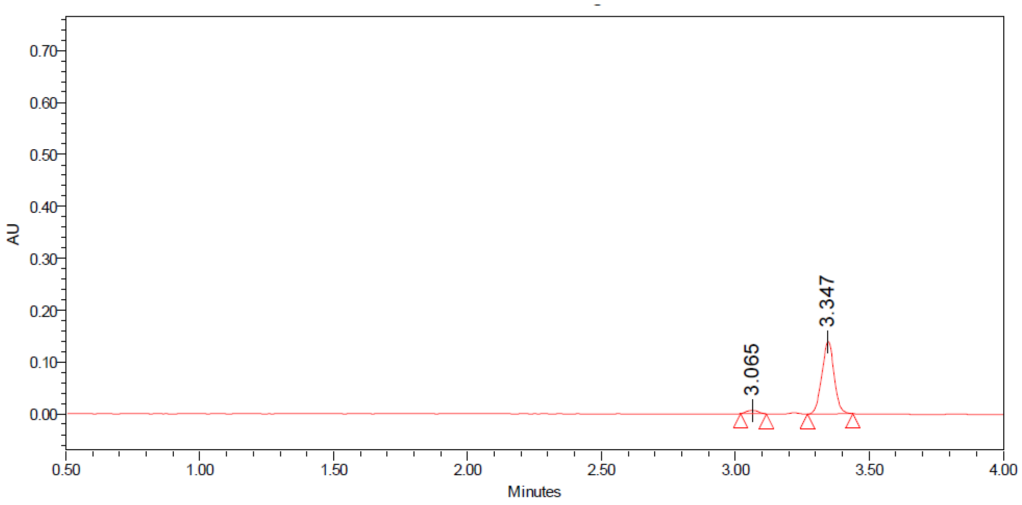

Peak Results

	RT	% Area
1	3.065	4.27
2	3.347	95.73

(3*R*,4*R*,5*S*)-Diethyl 4-(3-cyano-2-oxo-2*H*-chromen-4-yl)-5-(2-hydroxyphenyl)-3-(4-nitrophenyl)pyrrolidine-2,2-dicarboxylate **3f**

Racemic sample

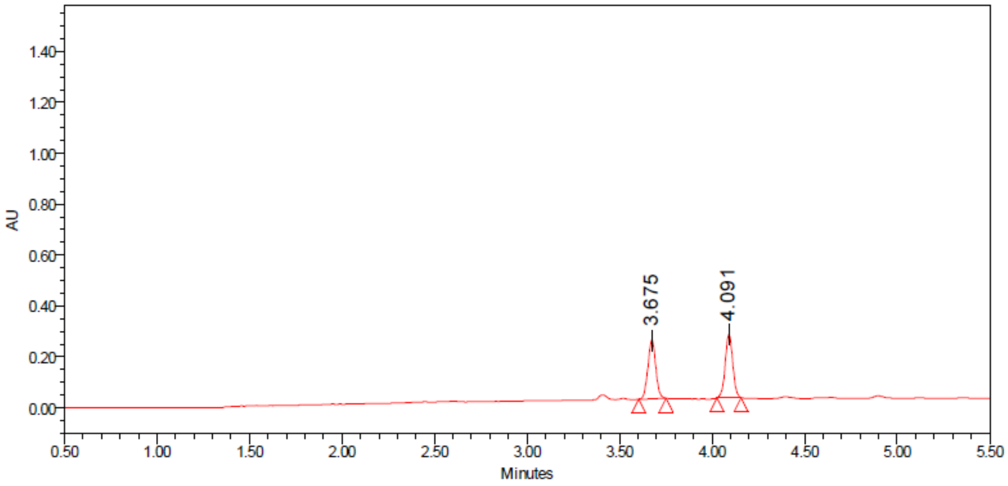

Peak Results

	RT	% Area
1	3.675	49.24
2	4.091	50.76

Enantiomerically enriched sample

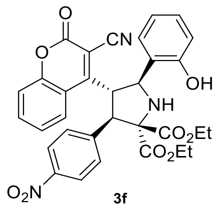

3f

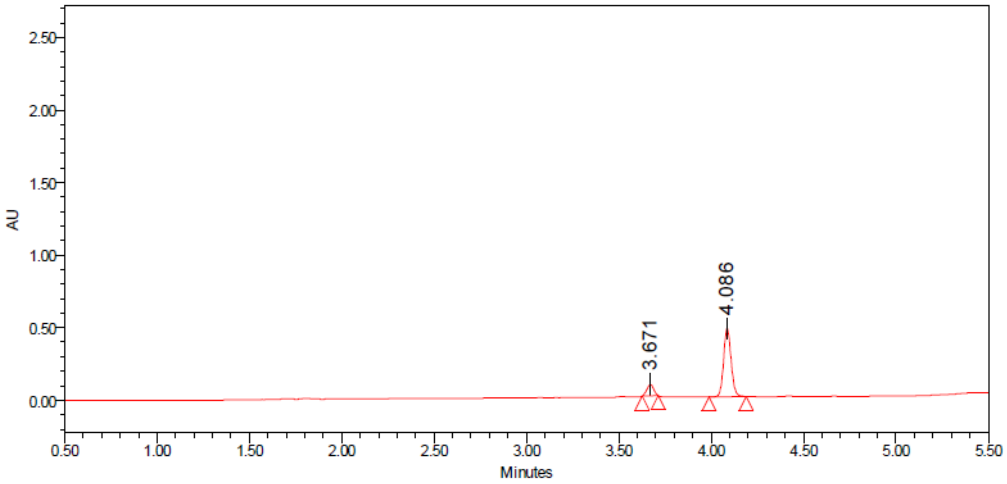

Peak Results

	RT	% Area
1	3.671	13.10
2	4.086	86.90

(3*R*,4*R*,5*S*)-Diethyl 4-(3-cyano-2-oxo-2*H*-chromen-4-yl)-5-(2-hydroxyphenyl)-3-(*p*-tolyl)-pyrrolidine-2,2-dicarboxylate **3g**

Racemic sample

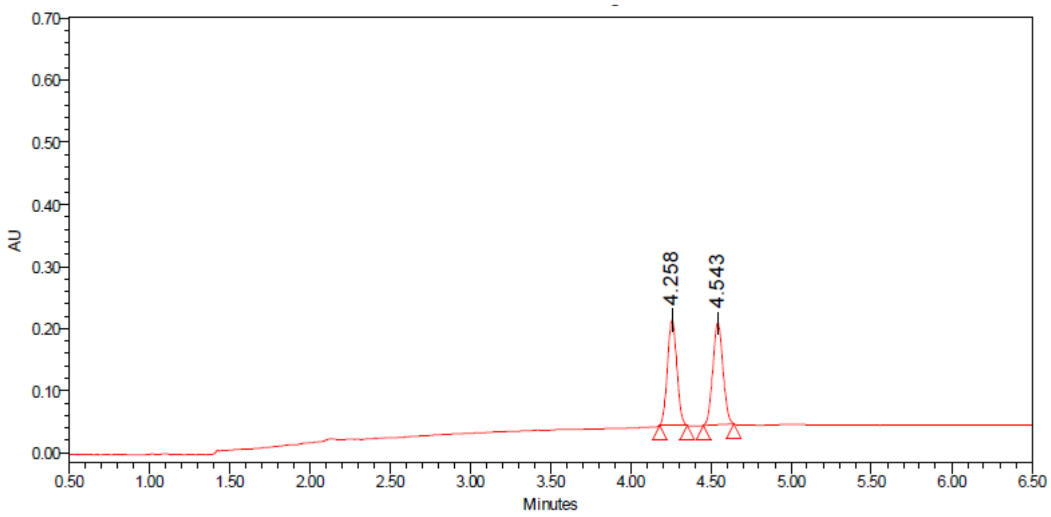

Peak Results

	RT	% Area
1	4.258	48.77
2	4.543	51.23

Enantiomerically enriched sample

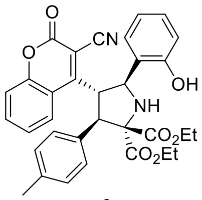

3g

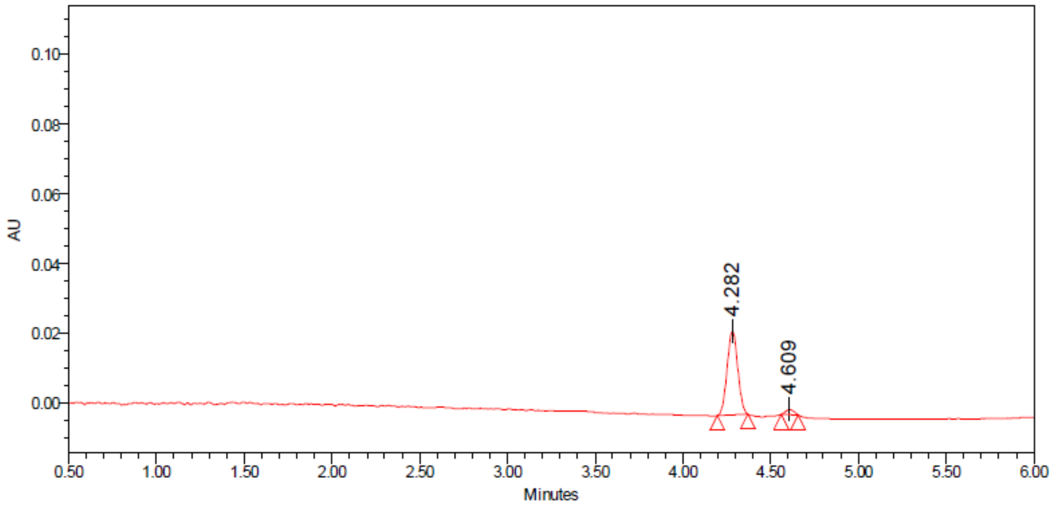

Peak Results

	RT	% Area
1	4.282	95.38
2	4.609	4.62

(3*R*,4*R*,5*S*)-Diethyl 4-(3-cyano-2-oxo-2*H*-chromen-4-yl)-5-(2-hydroxyphenyl)-3-(*m*-tolyl)-pyrrolidine-2,2-dicarboxylate **3h**

Racemic sample

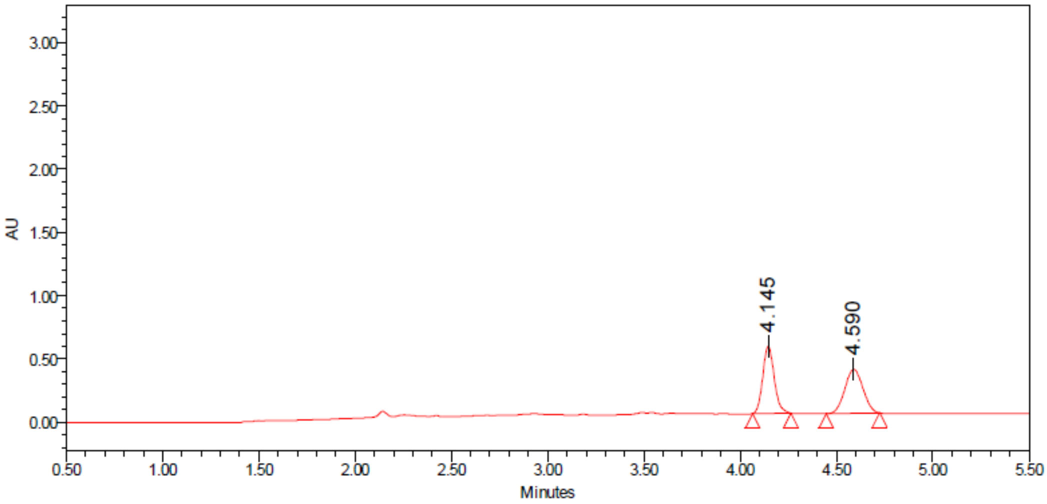

Peak Results

	RT	% Area
1	4.145	49.29
2	4.590	50.71

Enantiomerically enriched sample

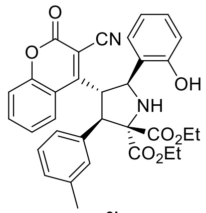

3h

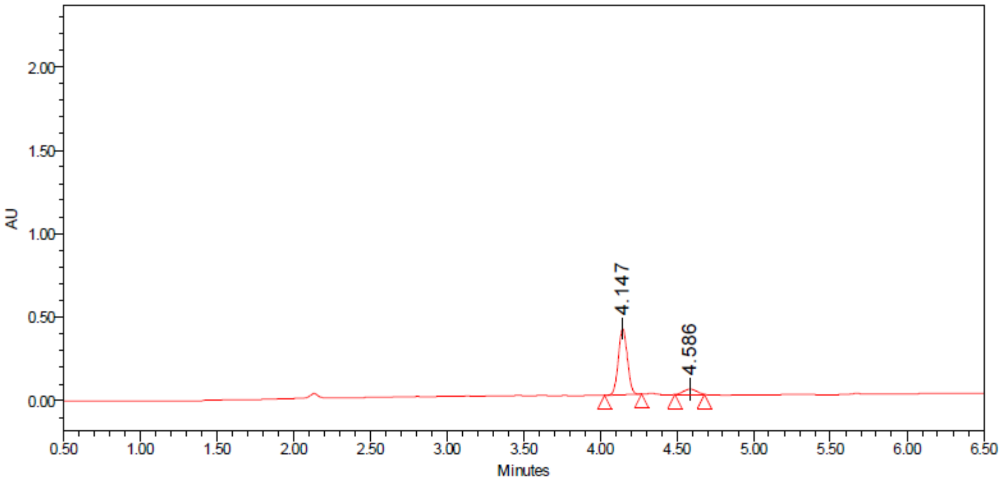

Peak Results

	RT	% Area
1	4.147	89.70
2	4.586	10.30

(3*R*,4*R*,5*S*)-Diethyl 4-(3-cyano-7-methoxy-2-oxo-2*H*-chromen-4-yl)-5-(2-hydroxyphenyl)-3-phenylpyrrolidine-2,2-dicarboxylate **3i**

Racemic sample

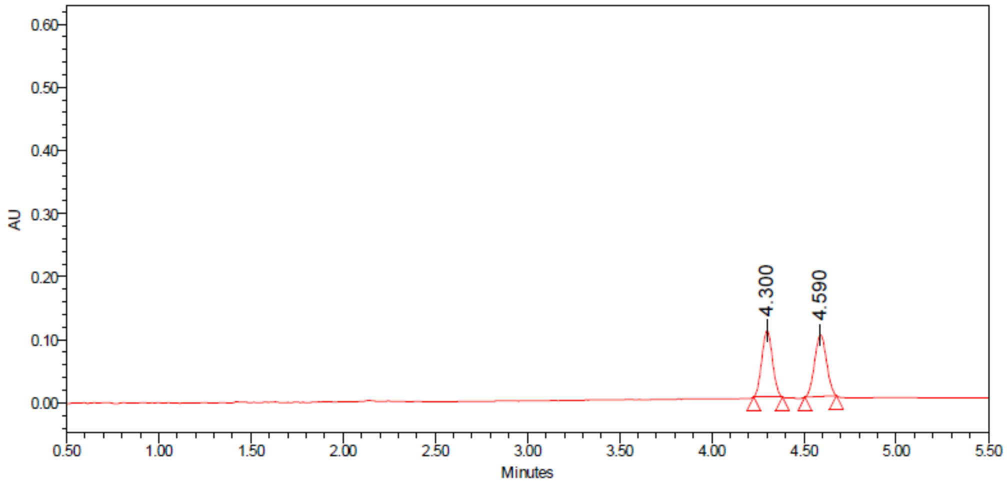

Peak Results

	RT	% Area
1	4.300	49.15
2	4.590	50.85

Enantiomerically enriched sample

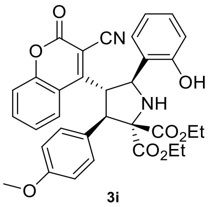

3i

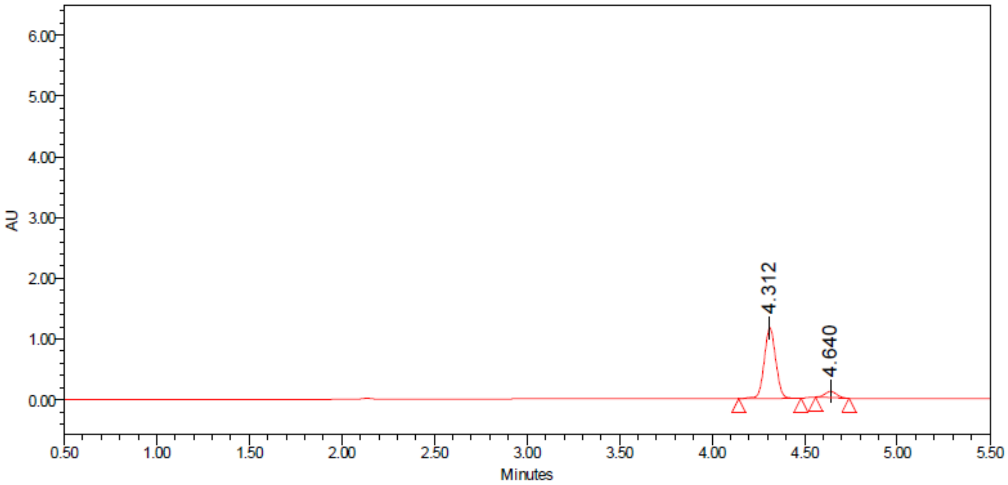

Peak Results

	RT	% Area
1	4.312	91.80
2	4.640	8.20

(3*R*,4*R*,5*S*)-Diethyl 4-(6-bromo-3-cyano-2-oxo-2*H*-chromen-4-yl)-5-(2-hydroxyphenyl)-3-phenylpyrrolidine-2,2-dicarboxylate **3j**

Racemic sample

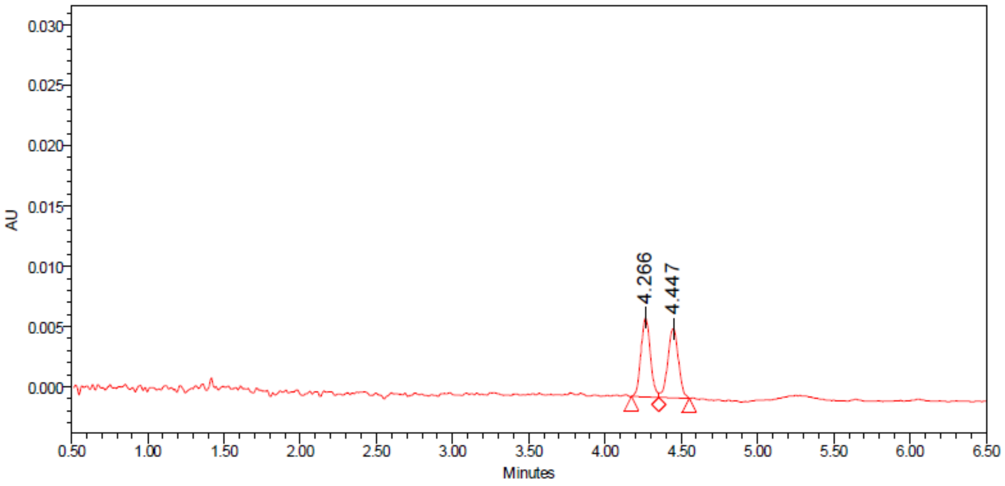

Peak Results

	RT	% Area
1	4.266	50.27
2	4.447	49.73

Enantiomerically enriched sample

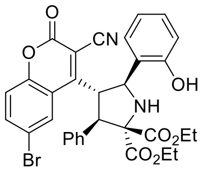

3j

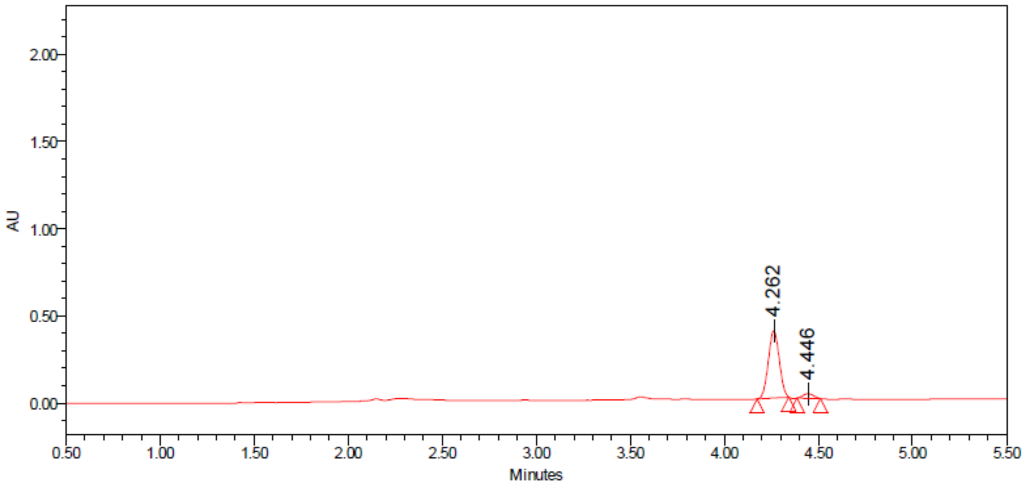

Peak Results

	RT	% Area
1	4.262	93.70
2	4.446	6.30

(3*R*,4*R*,5*S*)-Diethyl 4-(3-cyano-7-methoxy-2-oxo-2*H*-chromen-4-yl)-5-(2-hydroxyphenyl)-3-phenylpyrrolidine-2,2-dicarboxylate **3k**

Racemic sample

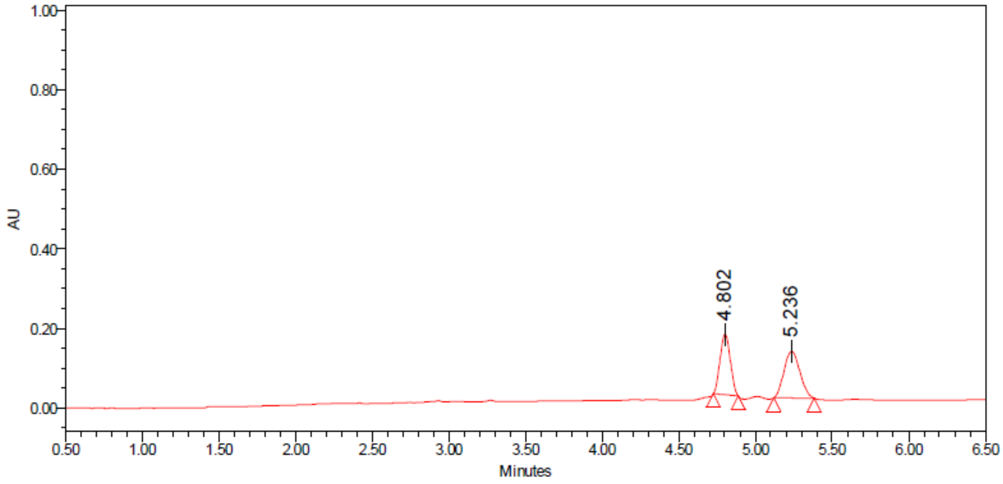

Peak Results

	RT	% Area
1	4.802	45.50
2	5.236	54.50

Enantiomerically enriched sample

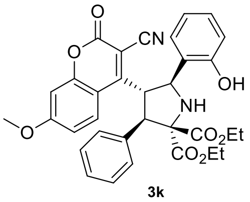

3k

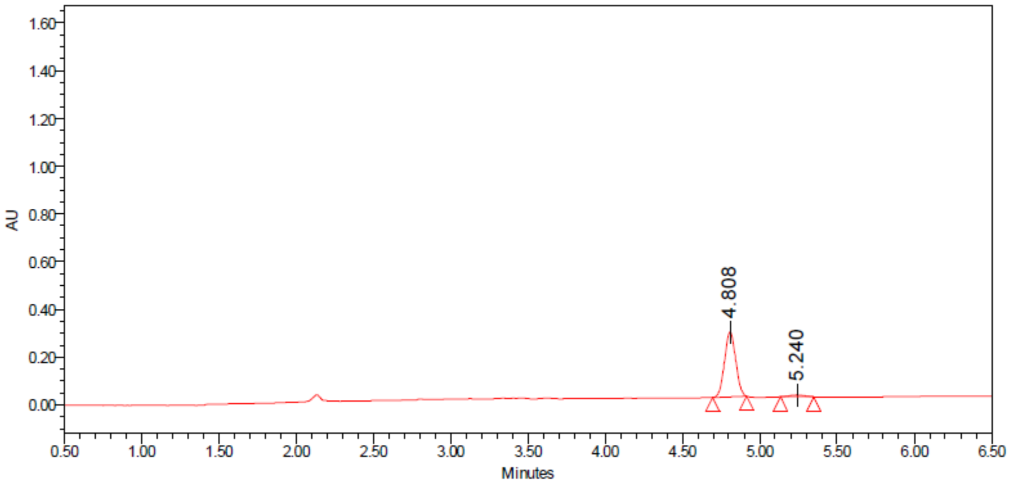

Peak Results

	RT	% Area
1	4.808	96.42
2	5.240	3.58

(3*R*,4*R*,5*S*)-Diethyl 5-(3-bromo-2-hydroxyphenyl)-4-(3-cyano-2-oxo-2*H*-chromen-4-yl)-3-phenylpyrrolidine-2,2-dicarboxylate **3m**

Racemic sample

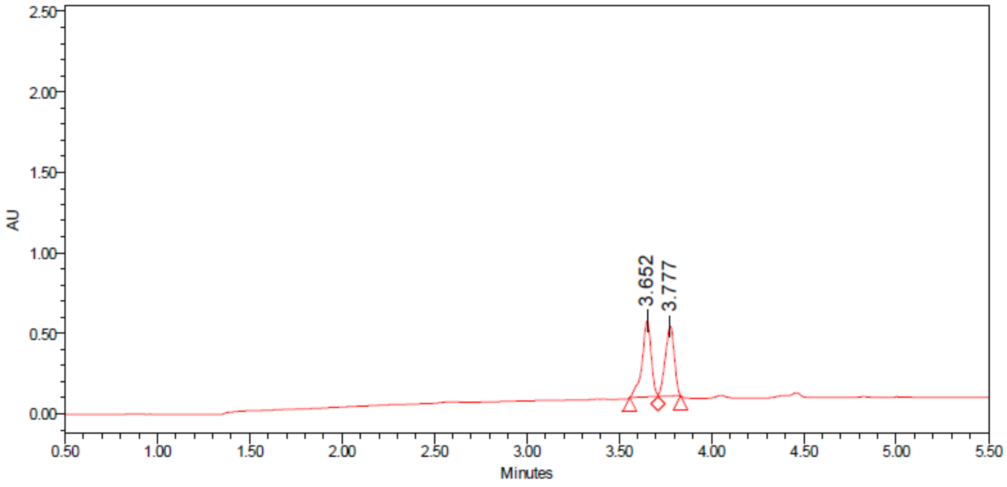

Peak Results

	RT	% Area
1	3.652	52.68
2	3.777	47.32

Enantiomerically enriched sample

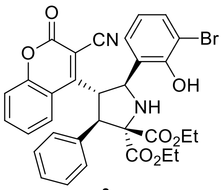

3m

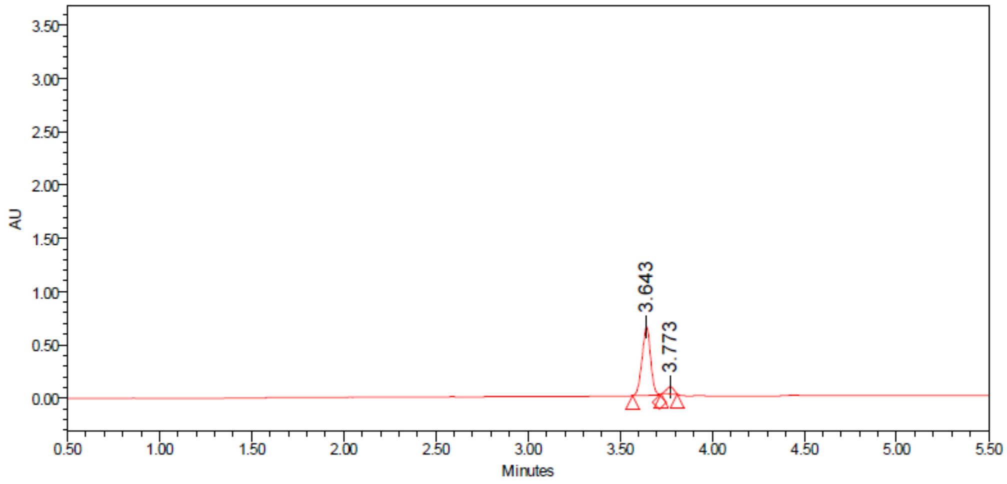

Peak Results

	RT	% Area
1	3.643	91.61
2	3.773	8.39

(3*R*,4*R*,5*S*)-Diethyl 5-(5-chloro-2-hydroxyphenyl)-4-(3-cyano-2-oxo-2*H*-chromen-4-yl)-3-phenylpyrrolidine-2,2-dicarboxylate **3n**

Racemic sample

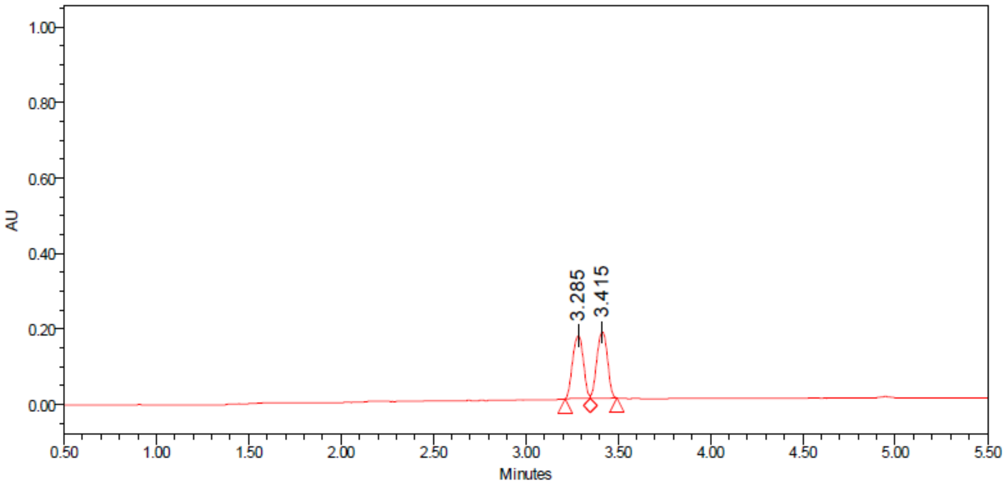

Peak Results

	RT	% Area
1	3.285	49.11
2	3.415	50.89

Enantiomerically enriched sample

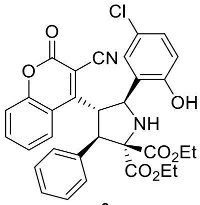

3n

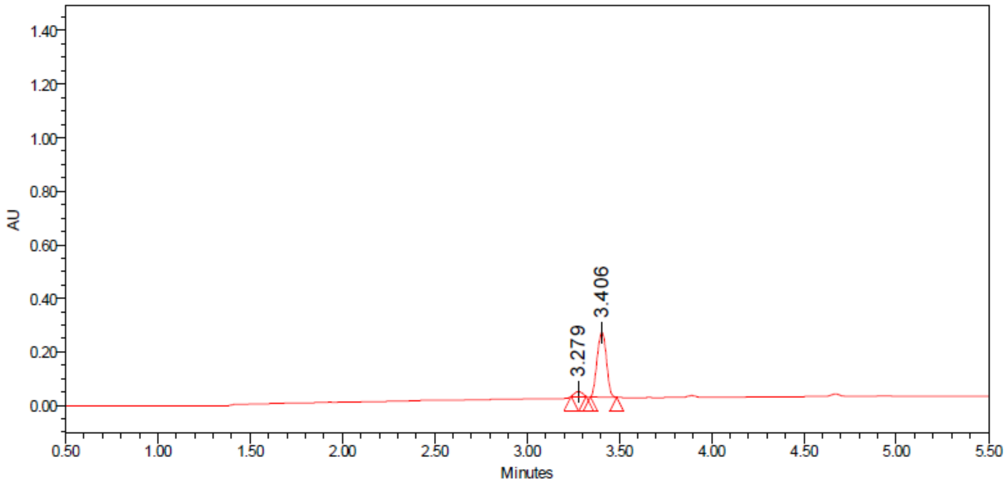

Peak Results

	RT	% Area
1	3.279	5.89
2	3.406	94.11

(3*R*,4*R*,5*S*)-Diethyl 4-(3-cyano-2-oxo-2*H*-chromen-4-yl)-5-(2-hydroxy-5-nitrophenyl)-3-phenylpyrrolidine-2,2-dicarboxylate **3o**

Racemic sample

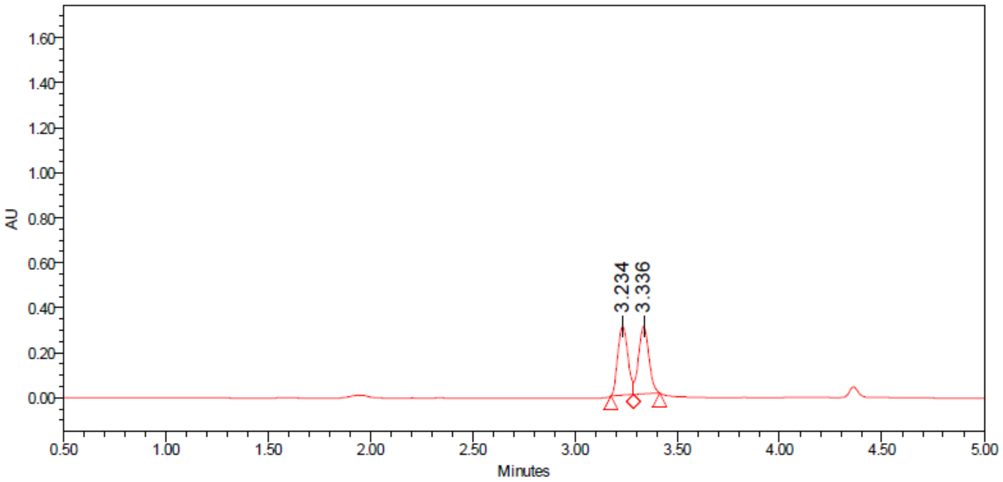

Peak Results

	RT	% Area
1	3.234	50.06
2	3.336	49.94

Enantiomerically enriched sample

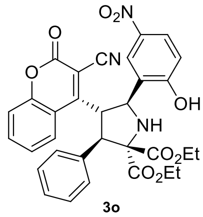

3o

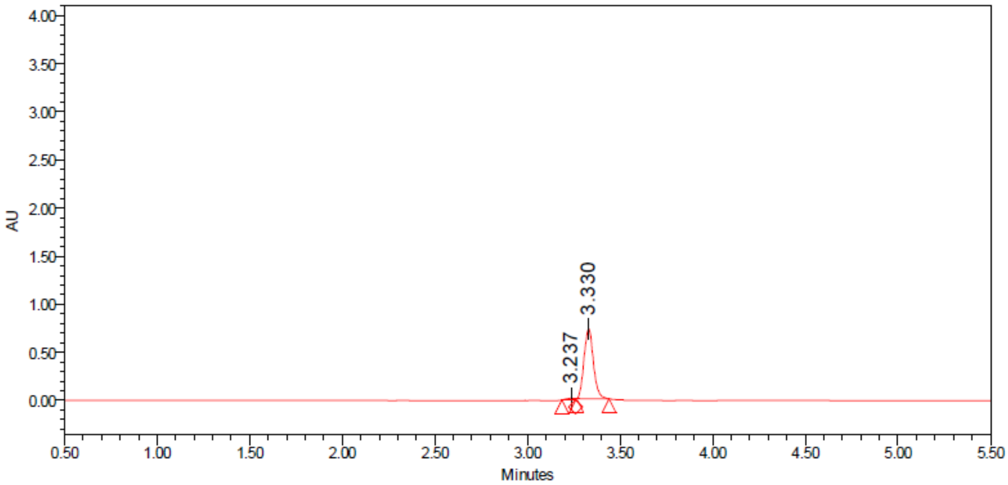

Peak Results

	RT	% Area
1	3.237	2.16
2	3.330	97.84

(3*R*,4*R*,5*S*)-Diethyl 4-(3-cyano-2-oxo-2*H*-chromen-4-yl)-5-(2-hydroxy-5-methylphenyl)-3-phenylpyrrolidine-2,2-dicarboxylate **3p**

Racemic sample

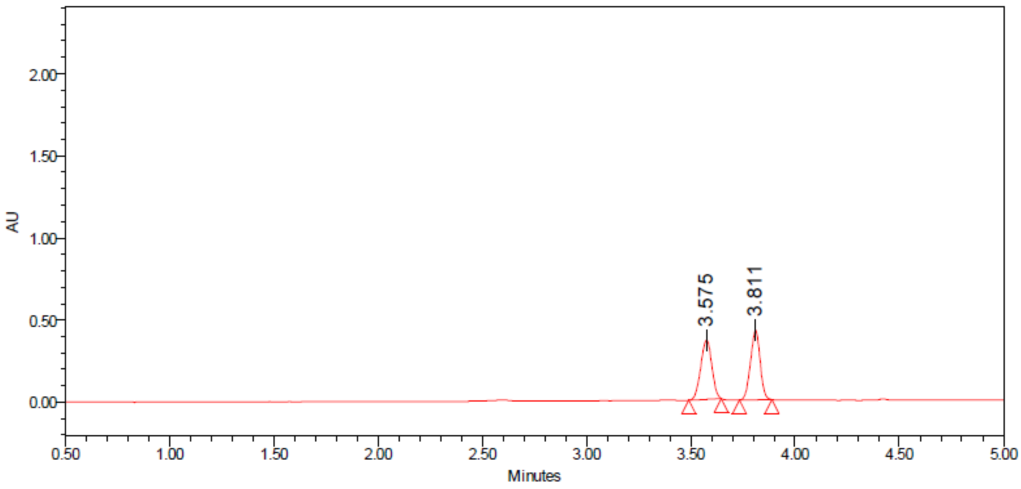

Peak Results

	RT	% Area
1	3.575	49.48
2	3.811	50.52

Enantiomerically enriched sample

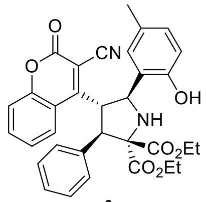

3p

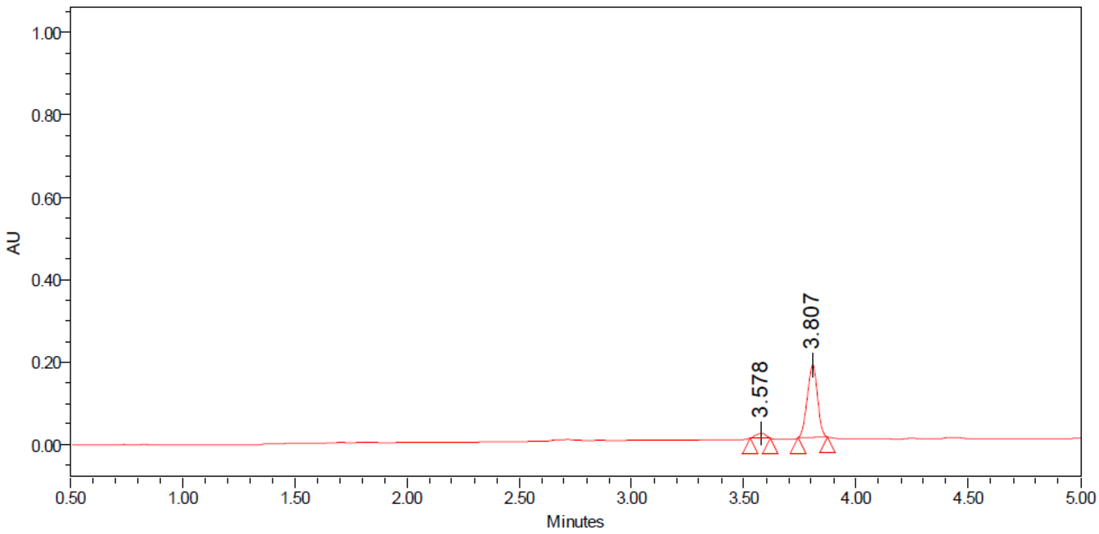

Peak Results

	RT	% Area
1	3.578	6.04
2	3.807	93.96

(3*R*,4*R*,5*S*)-Diethyl 5-(5-(*tert*-butyl)-2-hydroxyphenyl)-4-(3-cyano-2-oxo-2*H*-chromen-4-yl)-3-phenylpyrrolidine-2,2-dicarboxylate **3r**

Racemic sample

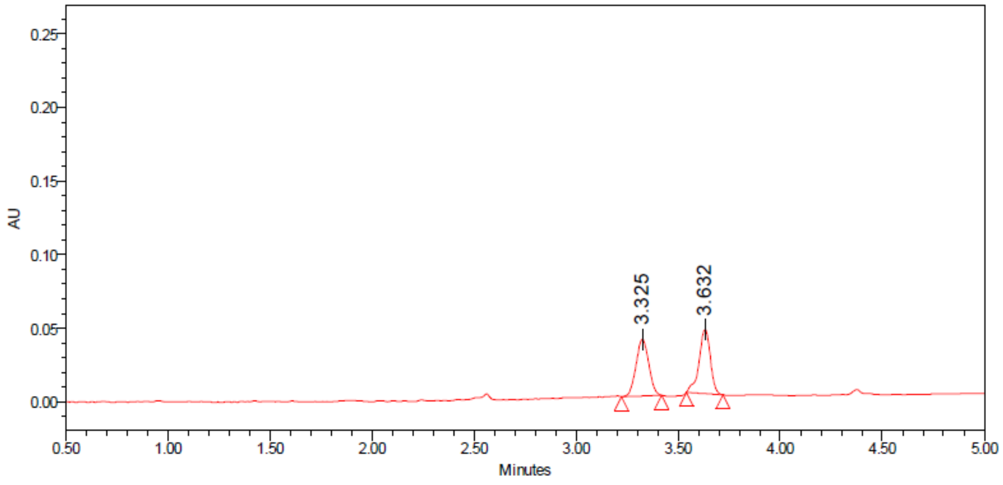

Peak Results

	RT	% Area
1	3.325	49.87
2	3.632	50.13

Enantiomerically enriched sample

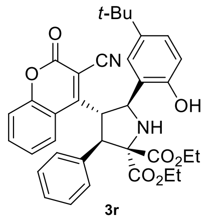

3r

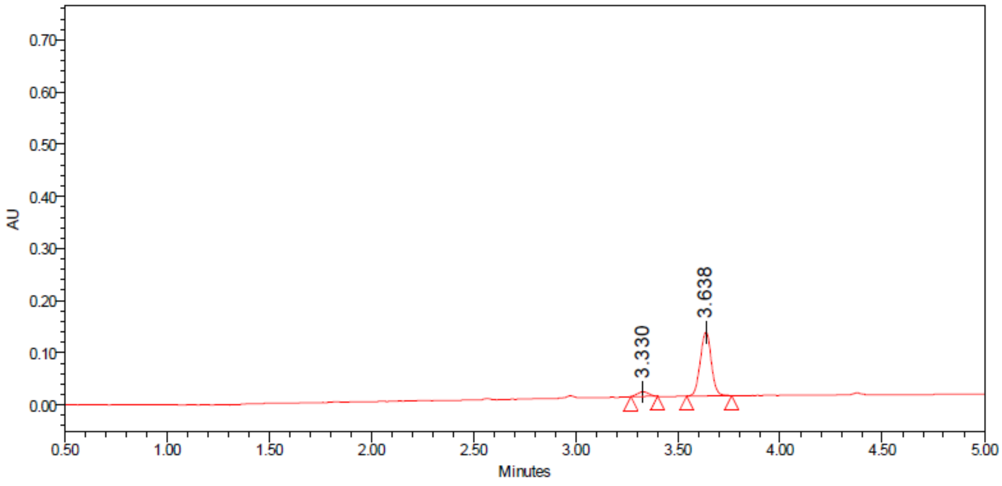

Peak Results

	RT	% Area
1	3.330	7.05
2	3.638	92.95

(3*R*,4*R*,5*S*)-Diethyl 4-(3-cyano-2-oxo-2*H*-chromen-4-yl)-5-(2-hydroxy-4,6-dimethoxyphenyl)-3-phenylpyrrolidine-2,2-dicarboxylate **3s**

Racemic sample

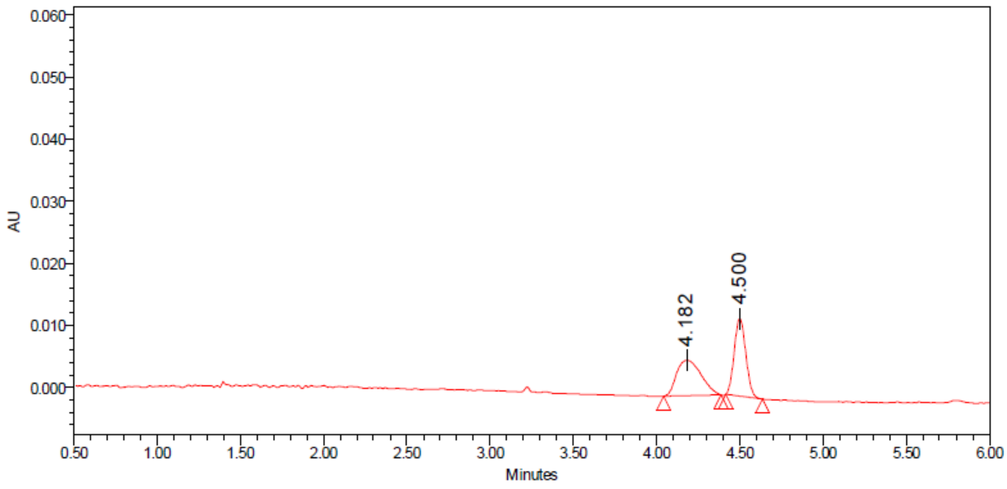

Peak Results

	RT	% Area
1	4.182	49.12
2	4.500	50.88

Enantiomerically enriched sample

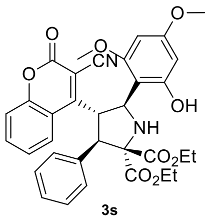

3s

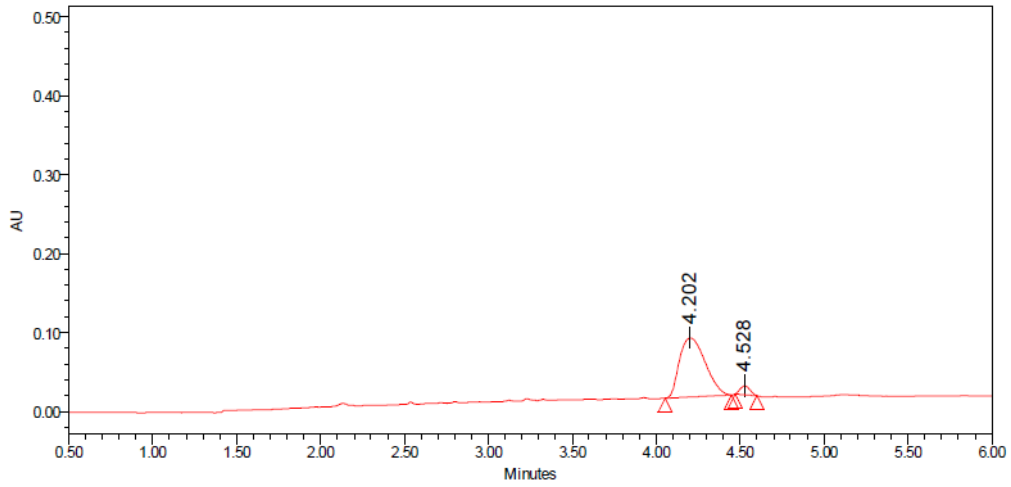

Peak Results

	RT	% Area
1	4.202	94.53
2	4.528	5.47

(1*R*,2*R*,10b*S*)-Diethyl 1-(3-cyano-2-oxo-2*H*-chromen-4-yl)-5-oxo-2-phenyl-5,10*b*-dihydro-1*H*-benzo-[*e*]pyrrolo[1,2-*c*][1,3]oxazine-3,3(2*H*)-dicarboxylate **5a**

Racemic sample

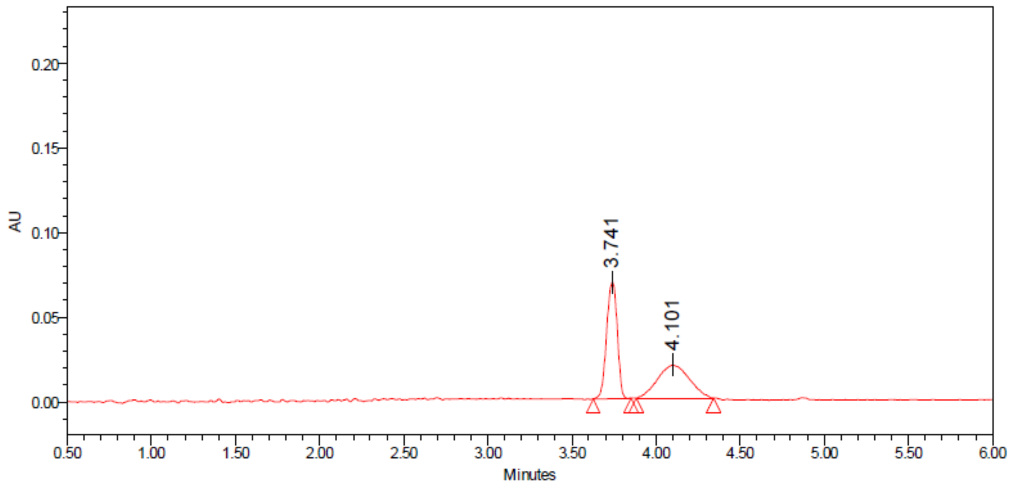

Peak Results

	RT	% Area
1	3.741	54.16
2	4.101	45.84

Enantiomerically enriched sample

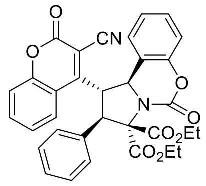

5a

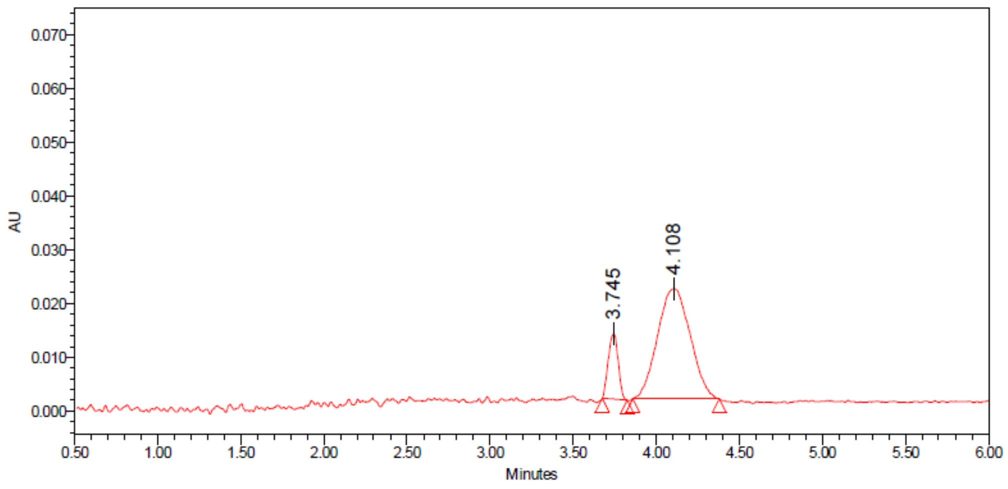

Peak Results

	RT	% Area
1	3.745	15.22
2	4.108	84.78

(1*R*,2*R*,10b*S*)-Diethyl 2-(4-chlorophenyl)-1-(3-cyano-2-oxo-2*H*-chromen-4-yl)-5-oxo-5,10*b*-dihydro-1*H*-benzo[e]-pyrrolo[1,2-*c*][1,3]oxazine-3,3(2*H*)-dicarboxylate **5b**

Racemic sample

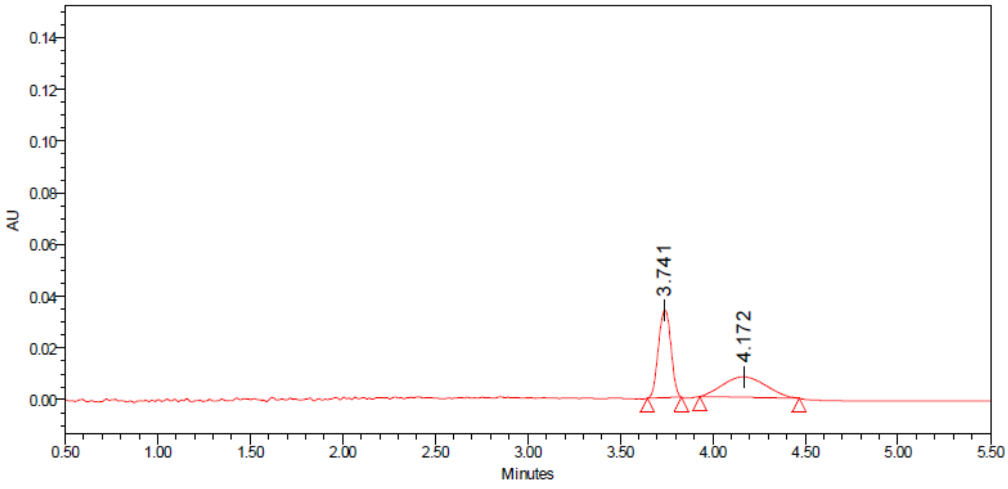

Peak Results

	RT	% Area
1	3.741	55.36
2	4.172	44.64

Enantiomerically enriched sample

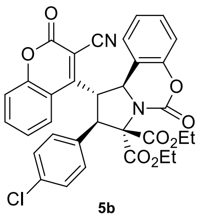

5b

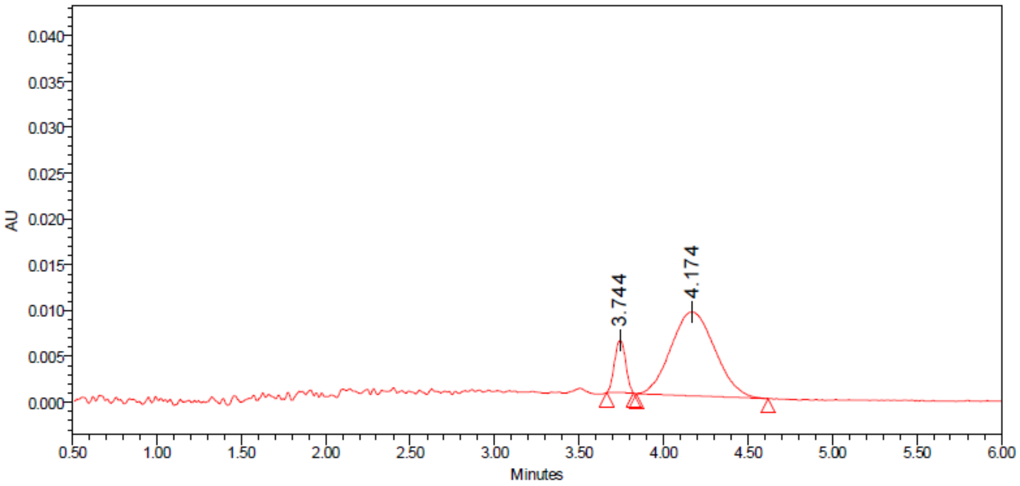

Peak Results

	RT	% Area
1	3.744	13.56
2	4.174	86.44

(1*R*,2*R*,10b*S*)-Diethyl 7-bromo-1-(3-cyano-2-oxo-2*H*-chromen-4-yl)-5-oxo-2-phenyl-5,10*b*-dihydro-1*H*-benzo[*e*]pyr-rolo[1,2-*c*][1,3]oxazine-3,3(2*H*)-dicarboxylate **5c**

Racemic sample

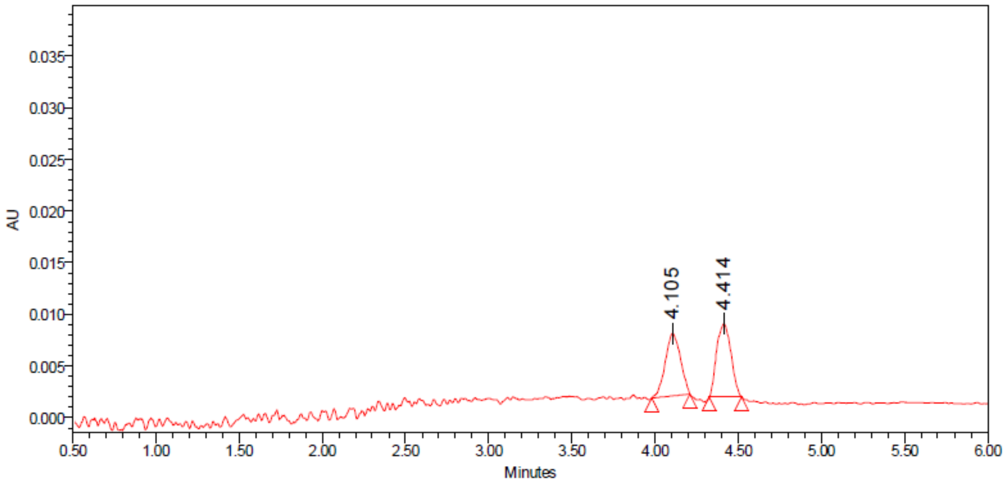

Peak Results

	RT	% Area
1	4.105	47.55
2	4.414	52.45

Enantiomerically enriched sample

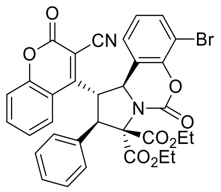

5c

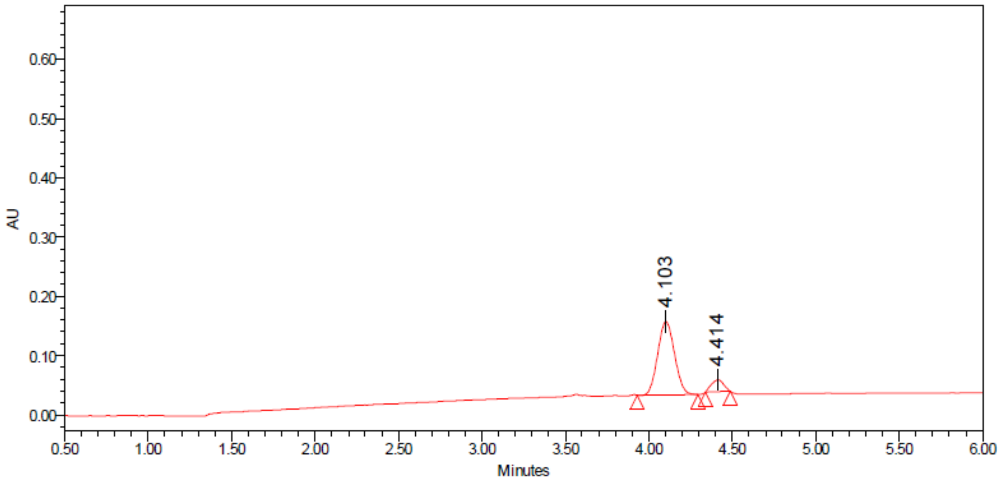

Peak Results

	RT	% Area
1	4.103	89.62
2	4.414	10.38
